# Evaluating Triazole-Substituted Pyrrolopyrimidines as CSF1R Inhibitors

**DOI:** 10.3390/molecules30122641

**Published:** 2025-06-18

**Authors:** Srinivasulu Cherukupalli, Jan Eickhoff, Carsten Degenhart, Peter Habenberger, Anke Unger, Bård Helge Hoff, Eirik Sundby

**Affiliations:** 1 Department of Chemistry, Norwegian University of Science and Technology (NTNU), Høgskoleringen 5, NO-7491 Trondheim, Norway; srinivasulu.cherukupalli@ntnu.no; 2 Lead Discovery Center GmbH (LDC), Otto-Hahn-Strasse 15, 44227 Dortmund, Germany; eickhoff@lead-discovery.de (J.E.); degenhart@lead-discovery.de (C.D.); habenberger@lead-discovery.de (P.H.); unger@lead-discovery.de (A.U.); 3 Department of Material Science, Norwegian University of Science and Technology (NTNU), NO-7491 Trondheim, Norway

**Keywords:** CSF1R, triazole, copper-catalyzed azide–alkyne cycloaddition, pyrrolopyrimidine

## Abstract

6-Aryl-7*H*-pyrrolo[2,3-*d*]pyrimidin-4-amines have promising properties as colony-stimulating factor 1 receptor (CSF1R) inhibitors. Inspired by these antagonists, two series of 1,2,3-triazole analogues (28 compounds) were synthesized and evaluated as CSF1R inhibitors. Enzymatic IC_50_ profiling showed that 27 of the 28 derivatives had lower IC_50_ than the reference drug PLX-3397. Three derivatives displayed CSF1R Ba/F3 cellular IC_50_ well below 1 µM. Profiling of the most promising triazole analogue (compound **27a**) toward a panel of kinases reveals a high selectivity for CSF1R with respect to its family kinases, but **27a** also inhibits ABL, SRC, and YES kinases. Molecular docking of **27a** toward two CSF1R X-ray structures identified two different ligand-inverted binding poses, which triggers interest for further investigations.

## 1. Introduction

**Figure 1:**
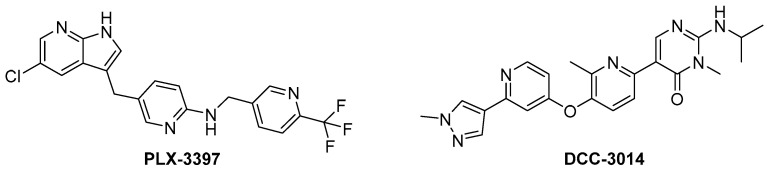
Structure of the FDA-approved CSF1R inhibitors PLX-3397 and DCC-3014.

Colony-stimulating factor 1 receptor (CSF1R) is a membrane-bound tyrosine kinase primarily expressed on the cell surface of monocyte/macrophage lineages, bone-resorbing osteoclasts, and microglial cells [1,2]. Upon binding of its cytokine ligands, colony-stimulating factor 1 (CSF1), and interleukin 34 (IL-34), CSF1R mediates signaling via downstream kinase pathways [3,4] necessary for the differentiation, proliferation, and normal function of these immune cells. However, in some diseases, the overexpression of CSF1 and/or elevated activity of CSF1R cause a misbalance of the immune cell phenotypes [5], leading to aggressive disease and poor prognosis [6,7]. Taking macrophages as examples, the simplified view is that they can have a tumoricidal (often called M1) phenotype, or a phenotype that promotes tumor growth, angiogenesis, invasion, and metastasis (often denoted as M2). Thus, finding ways of correcting the misbalance in the population of immune cells is of interest. The use of CSF1R inhibitors is one option with prospects in the treatment of cancers [8], inflammation-driven diseases [9], CNS diseases [10,11,12], and bone diseases [4,13]. There are currently two FDA-approved CSF1R inhibitors, pexidartinib (PLX-3397) and vimseltinib (DCC-3014), both used as a treatment for tenosynovial giant-cell tumors. The structures are shown in Figure 1. Clinical trials are also ongoing with other small molecular inhibitors and monoclonal antibodies [14,15]. CSF1R activity is also prominent in a few other FDA-approved kinase inhibitors such as dasatinib (multi-kinase inhibitor), imatinib (ABL-BCR), and surufatinib [15] (fibroblast growth factor receptor 1 inhibitor).

The kinase selectivity among small molecular CSF1R inhibitors varies, with some of the more selective inhibitors being DCC-3014) [16], sotuletinib (BLZ945) [17], and edicotinib (JNJ-40346527) [18,19]. Our CSF1R drug discovery efforts have focused on pyrrolopyrimidines [20,21,22] and purines [23]. Despite showing high enzymatic inhibition and excellent kinase selectivity, none of these derivatives have reached animal efficacy studies due to low metabolic stability in mice or low permeability [20]. These inhibitors feature two carbon-based aromatic substituents. While the presence of multiple carboaromatic rings in drug candidates is often associated with suboptimal physicochemical properties [24], these limitations can be mitigated by incorporating heterocycles such as triazoles [25,26]. Triazoles are widely utilized in medicinal chemistry [27,28,29] and are frequently found in kinase inhibitors [30,31,32,33,34,35,36]. In this study, we report the design, synthesis, and biological evaluation of a series of pyrrolopyrimidine-based CSF1R inhibitors bearing two distinct amines at the C4 position and eighteen different 1,2,3-triazole moieties at C6. The compounds were synthesized via Sonogashira cross-coupling followed by copper-catalyzed azide–alkyne cycloaddition (CuAAC). All derivatives were assessed in both enzymatic and cellular assays, and selected compounds were further evaluated for metabolic stability, permeability, and kinase selectivity. Additionally, molecular modeling was employed to explore the binding modes of the inhibitors.

## 2. Results and Discussion

### 2.1. Designs of the Inhibitors

**Figure 2:**
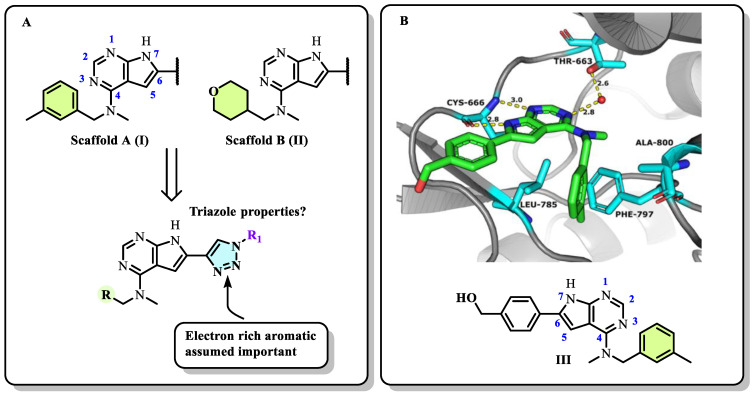
(**A**) Design concept for the triazole-based CSF1R inhibitors. (**B**) X-ray co-crystal structure of CSF1R (PDB 8CGC). The inhibitor is shown in green and hydrogen bonds to the protein is shown with dashed yellow lines. The structure of inhibitor **III** is shown with numbering system.

In our previous series of pyrrolopyrimidine-based CSF1R inhibitors, we identified two privileged substructures (Scaffold A (**I**) and Scaffold B (**II**), Figure 2A). An X-ray co-crystal structure of inhibitor **III** (Figure 2B) with CSF1R attributed its high activity to the hydrogen bonding of N1 and HN7 to Cys-666 in the hinge region, a water bridging interaction involving N3 and Thr-663, and lipophilic interactions for the *N*-methyl-*N*-(3-methylbenzyl) group at C4 with Leu-785 and Ala-800 (Figure 2B). It was assumed that the high selectivity was due to the placement of the *meta*-methyl group in a cavity in the front binding cleft [20]. Substructure **II**, which leads to even more potent inhibitors, is assumed to have more intimate contact with the protein alongside a hydrogen bond interaction from the tetrahydropyran (THP) oxygen to Arg-801 [22]. The benefit of inhibitors based on substructure **II** is higher solubility, though kinase selectivity seems to be somewhat reduced [22]. Aromatic groups at C6 also increase potency. Further, an *N*-methyl group at the 4-position is necessary to suppress epidermal growth factor receptor kinase’s (EGFR) inhibitory activity. When the aromatic structure at C6 was decorated with electron-withdrawing groups, a drop in activity was seen [22], possibly weakening cation-π interactions with Lys-586. Thus, we wanted to see how the replacement of carbo-aromatics with triazoles affected CSF1R inhibitory activity and molecular properties (Figure 2A).

### 2.2. Chemistry

**Scheme 1:**
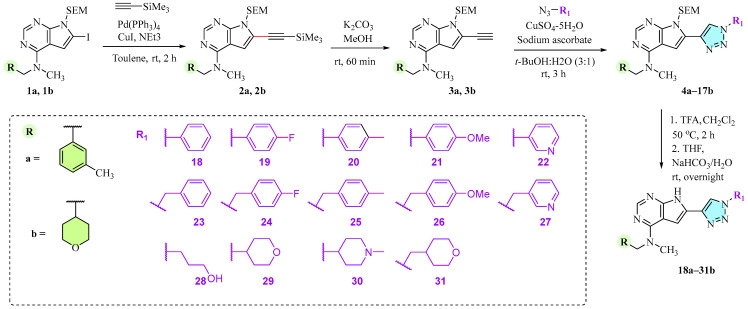
Synthesis of 1,2,3-triazole containing pyrrolo[2,3-*d*]pyrimidines **18a**–**31b**.

The chemistry of these triazoles is shown in Scheme 1. First, the 2-(trimethylsilyl)ethoxymethyl (SEM)-protected building blocks **1a** [20] and **1b** [22] were reacted with ethynyltrimethylsilane at C6 in a Sonogashira cross-coupling reaction using Pd(PPh_3_)_4_ in combination with CuI in 87% and 73% isolated yields, respectively.

Deprotection of the trimethylsilyl (TMS) group in **2a** and **2b** with K_2_CO_3_/MeOH produced the key intermediates **3a** and **3b** in 84% and 85% yields. These alkynes were then submitted to CuAAC with 14 different azides in *t*-BuOH/water (3:1) to give the triazoles **4a**–**17b** in 40–86% yields. We also attempted CuAAC on the non-protected pyrrolopyrimidines using the same conditions. However, possibly due to Cu-coordination to the heterocycles, no product was formed. Finally, the SEM-protected triazoles **4a**–**17b** were deprotected with trifluoroacetic acid (TFA) followed by treatment with aqueous NaHCO_3_ in THF to give 28 triazoles (**18a**–**31b)** in 62–98% isolated yields.

### 2.3. Enzymatic and Cellular Characterisation

The initial biological profiling (Table 1 and Table 2) was performed with enzymatic inhibition using a time-resolved Förster resonance energy transfer (TR-FRET) assay at an ATP concentration of 25 µM.

This was followed by cellular profiling with CSF1R Ba/F3 cells and in an IL-3 dependent Ba/F3 cell line (CSF1R independent) to indicate cellular toxicity. Table 1 summarizes the calculated logP (cLogP), the calculated polar surface area (PSA), and the measure kinetic solubility, enzymatic IC_50_ values, and the Ba/F3 cellular IC_50_ for the series A of triazoles (**18a**–**31a**). The data for series B of triazoles are shown in Table 2.

Profiling of **18a**–**31a** in enzymatic studies showed that except for the *para*-methyl analogue **20a**, the compounds have lower IC_50_ than the reference drug PLX-3397. For triazoles having directly attached aryl groups (comp. **18a**–**22a**), *para*-substitution lowers the enzymatic potency. The same trend is also seen in the benzylic series of triazoles (comp. **23a**–**27a**), while compounds having groups with higher polarity (comp **28a**–**31a**) were all extremely potent in the enzymatic assay. However, although clogP was below 4 for all compounds, they had limited kinetic solubility. The low solubility prevents the identification of meaningful cellular IC_50_ for derivatives **18a**–**21a** and **23a**–**25a**. Of the more soluble compounds, the 3-pyridyl analogue **27a** appeared most promising with a cellular IC_50_ of 0.3 µM. None of the compounds showed any major effect in the IL-3-dependent Ba/F3 (CSF1R-independent) assay, which indicates low general cellular toxicity. On average, higher solubility and slightly more potent derivatives were seen for the THP-containing inhibitors (series B) than for series A. The enzymatic IC_50_ ranged from 1–9 nM. As in the A-series, *para*-substitution of the aryl group (comp. **19b**–**21b**) lowers potency and, again, the *para*-methyl analogue (comp. **20b)** was the least active. Triazoles substituted with benzylic type substituents (comp. **23b**–**27b**) and polar substituents (**28b**–**31b**) were all highly active in the enzymatic assay. The cell activity of the series B triazoles correlates poorly with the enzymatic data. These studies indicated the *para*-methoxy **21b** (IC_50_ = 0.6 µM) and the *para*-methylbenzyl **25b** (IC_50_ = 1.0 µM) as most interesting for further evaluation. Both these compounds show higher cell potency than seen for their series A-analogues (Table 1, comp. **21a** and **35a**). No measurable cell activity was seen for **18b**, **19b,** and **29b,** in line with that seen in series A. More surprisingly, the propanol-substituted **28b**, in contrast to that seen for **28a** (Table 1, IC_50_ = 1.0 µM), was also inactive.

Overall, 27 of the 28 derivatives exhibited lower enzymatic IC_50_ values than PLX-3397, but none achieved the same high level of cellular potency as the reference compound. While this can partly be attributed to the physicochemical properties of the inhibitors, differences in off-target profiles cannot be entirely ruled out. For instance, PLX-3397 has also been reported to inhibit KIT, CFK19, KDR, LCK, FLT1, and TRKC [37]. Another possible explanation involves the different activation forms or conformations adopted by CSF1R [38,39]. It is possible that PLX-3397 and the triazoles preferentially bind to different activation forms, with the form targeted by PLX-3397 being more prevalent in cellular environments. If this is the case, the truncated enzymatic model used in the assay may be a suboptimal mimic of the membrane-bound enzyme.

### 2.4. Initial ADME and Kinase Off-Targets

**Figure 3:**
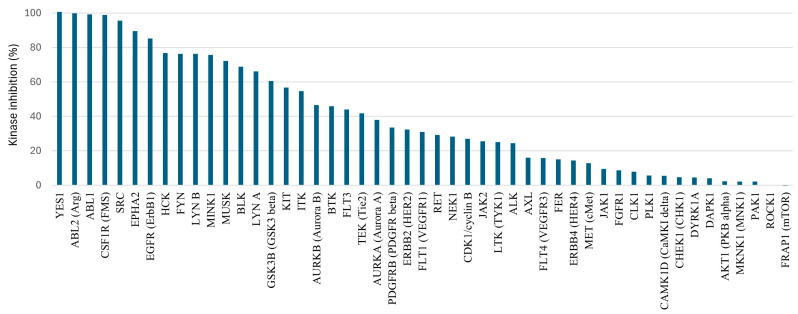
Profiling of compound **27a** toward a panel of kinases at 1000 nM using ATP level equal to K_M_.

Assuming that the cellular Ba/F3 CSF1R assay is a better model of in vivo conditions than the enzymatic assay, the *para*-methoxyphenyl derivative **21b**, the *para*-methylbenzyl **25b,** and the 3-pyridylmethyl **27a** were selected for further profiling (Table 3). The data are compared with two previously identified inhibitors **III** and **IV** [22], and PLX-3397. Firstly, the high enzymatic potency was confirmed by IC_50_ measurement using an alternative protocol (Z-LYTE) [38]. All compounds were also highly potent using this method (IC_50_ 0.4–1.3 nM). A typical off-target for CSF1R inhibitors is the structurally related KIT kinase. KIT enzymatic inhibition (*K*_D_) was measured using the TR-FRET acceptor dye, kinase tracer 236, and an anti-GST antibody containing a TR-FRET donor dye, which binds to the GST tag at the N-terminus of KIT. The binding of an inhibitor to the kinase displaces the tracer resulting in a loss of FRET. Cellular proliferation for KIT activity was performed using the KIT-positive cell line GIST-T1 [39]. The enzymatic profiling compiled in Table 3 shows that the triazoles have low activity toward KIT (*K*_D_ > 100 nM), in contrast to that of PLX-3397 (*K*_D_ = 28 nM µM). The reference compounds **III** and **IV** also have low KIT activity, but KIT profiling in the case of **III** was performed by an alternative assay [22]. Cellular profiling in the KIT-positive cell line confirmed the data from the enzymatic assay. The 3-pyridyl analogue **27a** was also profiled toward a panel of 49 kinases at a 1000 nM test concentration (Figure 3). The selection of kinases includes the structurally related kinases KIT, FLT3, and PDGFRB, some linked to cardiac toxicity [40], some previously identified off-targets for pyrrolopyrimidines [41,42], and common oncogenic targets [43]. In line with the KIT assay, lower activity was noted for the PDGFR family of kinases (FLT3, KIT, and PDGFRB). However, in contrast to the previously identified inhibitors with carbon-based aromatics at C6, **27a** appears to be a multi-kinase inhibitor with especially high activity toward YES, ABL1, ABL2, SRC, and EPHA2 kinases. Calculation of the selectivity score (S-score) [43] using 50% and 30% inhibition as thresholds gave S-scores of 0.35 and 0.51, respectively. We had previously assumed that the *meta*-methyl at the *N*-methyl-*N*-(3-methylbenzyl) group at C4 was a major contributor to high kinase selectivity [20], but in light of the current investigation, this appears to be wrong. Pyrrolopyrimidines substituted with triazoles at C6 demonstrate potential as multi-kinase inhibitors rather than selective CSF1R inhibitors. Notably, the primary off-target kinases identified (ABL, SRC, YES, and EPHA2) are all of significant interest in oncology. ABL inhibitors are clinically established in the treatment of leukemia [44], while the inhibition of SRC and YES can mitigate the acquired resistance mechanism in cancer therapy [45,46,47]. Although no EPHA2 inhibitors have yet received regulatory approval, EPHA2 is recognized as a key regulator of tumorigenesis and disease progression across multiple cancer types [48].

To indicate the potential of **21b**, **25b**, and **27a** for further development as multi-kinase inhibitors, a limited set of in vitro ADME assays was performed (see Table 3). Unfortunately, all three inhibitors had high metabolic clearance in human liver microsome (HML) and mouse liver microsome (MLM) assays. The triazoles were especially unstable in the MLM assay, with an intrinsic clearance of 288–1980 µL/min/mg compared to PLX-3397, with structures **III** and **IV** having a clearance in the range of 22–41 µL/min/mg. Permeability, as measured by the MDCK assay, was appropriate for **21b** (Ratio P*_app_*: 1.3), while for **25b** and **27a**, a higher rate in the B-A direction was observed, indicating active transport. The protein binding was mediocre for **21b** (76%) and high for **25b** (97.5%) and **27b** (>99%). Overall, these assays did not motivate more advanced animal studies. Obviously, in this compound series, the metabolic soft spot must be identified, and appropriate measures must be taken to block degradation.

### 2.5. Molecular Modelling

To understand the binding interactions of the triazole series, compounds **21b**, **25b**, and **27a**, structure **III** (Figure 2), and PLX-3397 were docked using Glide (Schrödinger Release 2025-1) [49] into two different X-ray structures of PDB 8CGC [20], originally obtained as a co-crystal with structure **III** (Figure 2), and PDB 4R7H [37] co-crystallized with PLX-3397. The docking scores are shown in Table 4. Structure **III** and PLX-3397 naturally docked with high docking scores toward their respective X-ray structures but with much lower affinity toward the other CSF1R X-ray structure. Based on this, we assumed that all triazoles would follow the pattern displayed by structure **III** and show better binding to 8CGC than to 4R7H. This was also the case for **21b** and **25b**.

**Figure 4:**
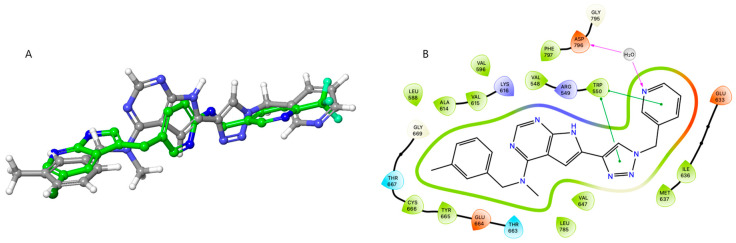
(**A**) Superposition of docked **27a** on CSF1R-PLX-3397 cocrystal structure 4R7H. (**B**) Suggested binding pose of **27a** using crystal structure 4R7H.

However, surprisingly, inhibitor **27a** was found to bind almost equally well to both X-ray constructs. Using our previously obtained X-ray co-crystal structure (8CGC), **27a** aligned well with the co-crystallized inhibitor **I**. In this pose, the *N*-methyl-*N*-(3-methylbenzyl) group at C-4 is directed inwards and the substituted triazole unit points toward the solvent-exposed region (Supplementary Materials, Figures S85 and S86). In docking with 4R7H, the PLX-3397-derived co-crystal, compound **27a** occupies approximately the same space as PLX-3397. Figure 4A shows the docking pose of **27a** superimposed by the X-ray pose of PLX-3397. In contrast to that seen when docking with 8CGC, the substituted triazole is now pointing inwards, and the *N*-methyl-*N*-(3-methylbenzyl) group at C-4 is directed toward the solvent-exposed area (see Figure 4B). In the alternative binding pose, compound **27a** is predicted to engage in a water-mediated hydrogen bond between the pyridyl nitrogen and Asp-796, a residue within the DFG motif. Additionally, π–π stacking interactions are observed between both the triazole and pyridine moieties of **27a** and the aromatic side chain of Trp-550. The nitrogen atoms N1 and N7 are positioned near the positively charged Arg-548, suggesting potential electrostatic interactions. Furthermore, the *N*-methyl-*N*-(3-methylbenzyl) substituent at the C4 position participates in hydrophobic interactions with the surrounding nonpolar residues.

In follow-up simulations, we performed docking of **27a** toward 8CGC and 4R7H with an induced fit protocol using Glide. The experiment with 8CGC identified 14 similar poses corresponding to that seen in Figure 2. Induced-fit docking with the construct 4R7H resulted in 7 “inverted” poses, as seen in Figure 4, with small variations in the ligand position and docking score (−13.195 to −12.310 Kcal/mol). This is despite a relatively large variation in the position of Phe-797 in the DFG motif (see Supplementary Materials, Figure S90). Additionally, one pose with apparent low binding (docking score: −7.807 Kcal/mol) was found to correspond to the binding mode shown in Figure 2. We cannot firmly conclude the validity of these two different poses. However, it is known that CSF1R is a dynamic protein existing in different conformations [50,51], and it is likely that CSF1R structure 8CGC [20] is a representative model of the autoinhibited form of the kinase, while 4R7H might be a better model for the non-autoinhibited form, in line with previous in vitro enzymatic assays [20]. In living cells, there will be an equilibrium of activation states, and **27a** is possibly able to inhibit several of these states.

## 3. Materials and Methods

### 3.1. Chemicals and Analysis

The solvents were purchased from Merck and VWR. The pyrrolopyrimidine intermediates **1a** and **1b** were made as previously described [20,22]. Ethynyltrimethylsilane, azidobenzene, 1-azido-4-fluorobenzene, 1-azido-4-methylbenzene, 1-azido-4-methoxybenzene, and benzyl azide were from Merck (Sigma-Aldrich), while the rest of the azides were from SynthonX. Nuclear magnetic resonance spectra were obtained with a Bruker Avance III HD 400 or 600 MHz instrument with DMSO-*d*_6_ as the solvent. High-resolution mass spectroscopy was performed on a Synapt G2-Q-TOF instrument from Waters (Milford, MA, USA) in positive mode using an electrospray (ES) probe. Processing was performed using Waters Software Masslynx V4.1 SCN871. The HPLC purity of the inhibitor candidates was determined with two methods: Method A [22]: Waters Acquity UPLC system, a Waters Acquity BEH C18 (50 × 2.1 mm, 1.7 μm) column, flow rate: 0.5 mL/min, column temperature: 60 °C; gradient elution with MeCN/H_2_O was performed as follows: 5% MeCN for 0.5 min, then a linear gradient up to 95% MeCN over 7.5 min, and finally 95% MeCN for 1.5 min; Method B: Agilent 1200 series instrument with an Agilent ObenLab ChemStation software version C.01.07(27). Column: Kinetex^®^ 5 µM C18 100 Å, 150 × 4.6 mm, eluent: H_2_O/CH_3_CN (10:90) to 100% CH_3_CN over 15 min, 5 min hold time at 100% CH_3_CN. Molecular modeling was performed as previously described [22].

### 3.2. General Procedures

#### 3.2.1. General Procedure A: Synthesis of Triazole Intermediates

To a round-bottom flask containing t-BuOH/H2O (3:1 by vol, 8 mL), the ethynyl compound (**3a** or **3b**, 0.25 mmol), azide (1.3 equiv.), CuSO_4_ pentahydrate (0.1 equiv.), and sodium ascorbate (0.2 equiv.) were added. The reaction mixture was stirred at rt overnight. Upon reaction completion, the solvent was removed by evaporation. The residue was diluted with water and the mixture was extracted with CH_2_Cl_2_. The combined organic layers were washed with brine, dried over Na_2_SO_4_, and concentrated. The crude product was purified by silica-gel column chromatography to achieve the desired products as stated for each specific compound.

#### 3.2.2. General Procedure B: SEM-Deprotection

To the SEM-protected triazole intermediate (**4a**–**17b**; 60–120 mg, 1 equiv.), CH_2_Cl_2_ (10 mL) TFA (2 mL) were added and stirred at 50 °C for 2 h. The reaction mixture was then concentrated in vacuo before it was taken up in THF (10 mL) and NaHCO_3_ (20 mL, 25% aqueous) and stirred at rt overnight. For most of the compounds, the solvent was removed by evaporation, resulting in a precipitate. The precipitate was isolated by filtration and washed with water (10 mL) and *n*-pentane (2 × 10 mL). Some compounds, as specified, were purified by silica-gel column chromatography.

### 3.3. Inhibitor Candidates

#### 3.3.1. N-Methyl-N-(3-methylbenzyl)-6-(1-phenyl-1H-1,2,3-triazol-4-yl)-7H-pyrrolo[2,3-d]pyrimidin-4-amine (**18a**)

The reaction was run as described in General Procedure B using **4a** (60 mg, 0.11 mmol). This gave 35 mg (0.08 mmol, 78%) of a white powder, mp. 292–294 °C; HPLC method A: 99%; ^1^H NMR (400 MHz, DMSO-*d*_6_) *δ*: 12.37 (s, 1H), 9.03 (s, 1H), 8.18 (s, 1H), 7.89 (d, *J* = 7.3 Hz, 2H), 7.64 (t, *J* = 7.9 Hz, 2H), 7.55–7.51 (m, 1H), 7.22 (t, *J* = 7.5 Hz, 1H), 7.11–7.02 (m, 4H), 5.02 (s, 2H), 3.35 (s, 3H), 2.27 (s, 3H); ^13^C NMR (101 MHz, DMSO-*d*_6_) *δ*: 156.6, 152.5, 151.4, 141.0, 138.2, 137.6, 136.4, 130.0 (2C), 128.9, 128.4, 127.6, 127.5, 124.5, 124.0, 120.1 (2C), 119.1, 102.7, 99.2, 52.6, 37.1, 21.0; HRMS (ES+, *m*/*z*): found 396.1939, calcd for C_23_H_22_N_7_, [M+H]+, 396.1937.

#### 3.3.2. N-Methyl-6-(1-phenyl-1H-1,2,3-triazol-4-yl)-N-((tetrahydro-2H-pyran-4-yl)methyl)-7H-pyrrolo[2,3-d]pyrimidin-4-amine (**18b**)

The reaction was run as described in General Procedure B using **4b** (80 mg, 0.15 mmol). This gave 50 mg (0.12 mmol, 85%) of a white powder, mp. 294–296 °C; HPLC method A: 97%; ^1^H NMR (600 MHz, DMSO-*d*_6_) *δ*: 12.30 (s, 1H), 9.04 (s, 1H), 8.14 (s, 1H), 7.91 (d, *J* = 7.4 Hz, 2H), 7.67–7.64 (m, 2H), 7.54 (t, *J* = 7.4 Hz, 1H), 7.06 (s, 1H), 3.84 (dd, *J* = 11.5, 2.6 Hz, 2H), 3.69 (d, *J* = 7.4 Hz, 2H), 3.41 (s, 3H), 3.26 (td, *J* = 11.7, 2.1 Hz, 2H), 2.10–2.06 (m, 1H), 1.57–1.54 (m, 2H), 1.34–1.27 (m, 2H); ^13^C NMR (151 MHz, DMSO-*d*_6_) *δ*: 156.4, 152.4, 151.3, 141.1, 136.4, 130.0 (2C), 128.9, 124.2, 120.1 (2C), 119.1, 102.7, 99.4, 66.7 (2C), 55.4, 38.9, 33.9, 30.3 (2C), 25.1; HRMS (ES+, *m*/*z*): found 390.2042, calcd for C_21_H_24_N_7_O, [M+H]+, 390.2042.

#### 3.3.3. 6-(1-(4-Fluorophenyl)-1H-1,2,3-triazol-4-yl)-N-methyl-N-(3-methylbenzyl)-7H-pyrrolo[2,3-d]pyrimidin-4-amine (**19a**)

The reaction was run as described in General Procedure B using **5a** (110 mg, 0.20 mmol). This gave 67 mg (0.16 mmol, 81%) of a white powder, *mp*. 285–287 °C; HPLC method A: 98%; ^1^H NMR (600 MHz, DMSO-*d*_6_) *δ*: 12.36 (s, 1H), 8.99 (s, 1H), 8.18 (s, 1H), 7.95–7.93 (m, 2H), 7.50 (t, *J* = 8.7 Hz, 2H), 7.22 (t, *J* = 7.6 Hz, 1H), 7.10 (s, 1H), 7.07 (d, *J* = 7.4 Hz, 2H), 7.01 (s, 1H), 5.01 (s, 2H), 3.35 (s, 3H), 2.27 (s, 3H); ^13^C NMR (151 MHz, DMSO-*d*_6_) *δ*: 161.7 (d, *J* = 246.1 Hz), 156.6, 152.5, 151.4, 141.0, 138.2, 137.6, 133.0 (d, *J* = 2.8 Hz), 128.4, 127.6, 127.5, 124.4, 124.0, 122.60 (d, *J* = 8.8 Hz, 2C), 119.5, 116.9 (d, *J* = 23.3 Hz, 2C), 102.7, 99.3, 52.6, 37.1, 21.0.; HRMS (ES+, *m*/*z*): found 414.1845, calcd for C_23_H_21_FN_7_, [M+H]+, 414.1842.

#### 3.3.4. 6-(1-(4-Fluorophenyl)-1H-1,2,3-triazol-4-yl)-N-methyl-N-((tetrahydro-2H-pyran-4-yl)methyl)-7H-pyrrolo[2,3-d]pyrimidin-4-amine (**19b**)

The reaction was run as described in General Procedure B using **5b** (115 mg, 0.21 mmol). This gave 76 mg (0.18 mmol, 87%) of a white powder, mp. >300 °C; HPLC method A: 99%; ^1^H NMR (600 MHz, DMSO-*d*_6_) *δ*: 12.31 (s, 1H), 9.01 (s, 1H), 8.14 (s, 1H), 7.96–7.94 (m, 2H), 7.51 (t, *J* = 8.8 Hz, 2H), 7.05 (s, 1H), 3.84 (dd, *J* = 11.5, 2.6 Hz, 2H), 3.69 (d, *J* = 7.4 Hz, 2H), 3.40 (s, 3H), 3.25 (td, *J* = 11.7, 2.1 Hz, 2H), 2.10–2.05 (m, 1H), 1.57–1.54 (m, 2H), 1.34–1.27 (m, 2H); ^13^C NMR (151 MHz, DMSO-*d*_6_) *δ*: 161.7 (d, *J* = 246.0 Hz), 156.4, 152.4, 151.3, 141.1, 133.0 (d, *J* = 3.0 Hz), 124.1, 122.6 (d, *J* = 8.8 Hz, 2C), 119.4, 116.9 (d, *J* = 23.4 Hz, 2C), 102.7, 99.4, 66.7 (2C), 55.4, 38.8, 33.9, 30.3 (2C); HRMS (ES+, *m*/*z*): found 408.1947, calcd for C_21_H_23_FN_7_O, [M+H]+, 408.1948.

#### 3.3.5. N-Methyl-N-(3-methylbenzyl)-6-(1-(p-tolyl)-1H-1,2,3-triazol-4-yl)-7H-pyrrolo[2,3-d]pyrimidin-4-amine (**20a**)

The reaction was run as described in General Procedure B using **6a** (76 mg, 0.14 mmol). This gave 38 mg (0.09 mmol, 67%) of a white solid, mp. 250–252 °C; HPLC method A: 99%; ^1^H NMR (400 MHz, DMSO-*d*_6_) *δ*: 12.32 (s, 1H), 8.96 (s, 1H), 8.17 (s, 1H), 7.76 (d, *J* = 8.5 Hz, 2H), 7.43 (d, *J* = 8.2 Hz, 2H), 7.22 (t, *J* = 7.5 Hz, 1H), 7.11–7.06 (m, 3H), 7.00 (s, 1H), 5.01 (s, 2H), 3.35 (s, 3H), 2.40 (s, 3H), 2.27 (3H); ^13^C NMR (101 MHz, DMSO-*d*_6_) *δ*: 156.5, 152.8, 151.2, 141.3, 138.5, 138.3, 137.6, 134.2, 130.3 (2C), 128.4, 127.6, 127.5, 125.1, 124.0, 120.0 (2C), 119.0, 102.8, 98.9, 52.6, 37.1, 21.0, 20.5; HRMS (ES+, *m*/*z*): found 410.2097, calcd for C_24_H_24_N_7_, [M+H]+, 410.2093.

#### 3.3.6. N-Methyl-N-((tetrahydro-2H-pyran-4-yl)methyl)-6-(1-(p-tolyl)-1H-1,2,3-triazol-4-yl)-7H-pyrrolo[2,3-d]pyrimidin-4-amine (**20b**)

The reaction was run as described in General Procedure B using **6b** (80 mg, 0.15 mmol). This gave 40 mg (0.09 mmol, 67%) of a white powder, mp. > 300 °C; HPLC method A: 99%; ^1^H NMR (400 MHz, DMSO-*d*_6_) *δ*: 12.27 (s, 1H), 8.99 (s, 1H), 8.12 (s, 1H), 7.78 (d, *J* = 8.3 Hz, 2H), 7.45 (d, *J* = 8.1 Hz, 2H), 7.04 (s, 1H), 3.86–3.83 (m, 2H), 3.69 (d, *J* = 7.4 Hz, 2H), 3.40 (s, 3H), 3.28–3.23 (m, 2H), 2.41 (s, 3H), 2.08 (m, 1H), 1.56 (d, *J* = 12.9 Hz, 2H), 1.35–1.25 (m, 2H); ^13^C NMR (101 MHz, DMSO-*d*_6_) *δ*: 156.4, 152.6, 151.2, 141.2, 138.5, 134.2, 130.3 (2C), 124.6, 120.0 (2C), 119.0, 102.8, 99.1, 66.7 (2C), 55.4, 38.8, 33.9, 30.3 (2C), 20.6; HRMS (ES+, *m*/*z*): found 404.2197, calcd for C_22_H_26_N_7_O, [M+H]+, 404.2199.

#### 3.3.7. 6-(1-(4-Methoxyphenyl)-1H-1,2,3-triazol-4-yl)-N-methyl-N-(3-methylbenzyl)-7H-pyrrolo[2,3-d]pyrimidin-4-amine (**21a**)

The reaction was run as described in General Procedure B using **7a** (60 mg, 0.11 mmol). This gave 32 mg (0.074 mmol, 69%), mp. 248–250 °C; HPLC method A: 99%; ^1^H NMR (600 MHz, DMSO-*d*_6_) *δ*: 12.32 (s, 1H), 8.91 (s, 1H), 8.18 (s, 1H), 7.79 (d, *J* = 9.0 Hz, 2H), 7.22 (t, *J* = 7.6 Hz, 1H), 7.18 (d, *J* = 9.0 Hz, 2H), 7.10 (s, 1H), 7.07 (d, *J* = 7.6 Hz, 2H), 7.00 (s, 1H), 5.01 (s, 2H), 3.84 (s, 3H), 3.35 (s, 3H), 2.28 (s, 3H); ^13^C NMR (151 MHz, DMSO-*d*_6_) *δ*: 159.4, 156.5, 152.5, 151.3, 140.8, 138.2, 137.6, 129.8, 128.4, 127.6, 127.5, 124.6, 124.0, 121.8 (2C), 119.2, 115.0 (2C), 102.7, 99.0, 55.6, 52.6, 37.1, 21.0; HRMS (ES+, *m*/*z*): found 426.2040, calcd for C_24_H_24_N_7_O, [M+H]+, 426.2042.

#### 3.3.8. 6-(1-(4-Methoxyphenyl)-1H-1,2,3-triazol-4-yl)-N-methyl-N-((tetrahydro-2H-pyran-4-yl)methyl)-7H-pyrrolo[2,3-d]pyrimidin-4-amine (**21b**)

The reaction was run as described in General Procedure B using **7b** (65 mg, 0.16 mmol). This gave 40 mg (0.09 mmol, 81%) of a white powder, mp. > 300 °C; HPLC method A: 97%; HPLC method B: 99%; ^1^H NMR (600 MHz, DMSO-*d*_6_) *δ*: 12.28 (s, 1H), 8.93 (s, 1H), 8.14 (s, 1H), 7.81 (d, *J* = 9.0 Hz, 2H), 7.18 (d, *J* = 8.9 Hz, 2H), 7.04 (s, 1H), 3.85 (s, 3H), 3.84–3.83 (m, 2H), 3.69 (d, *J* = 7.4 Hz, 2H), 3.40 (s, 3H), 3.25 (td, *J* = 11.7, 2.0 Hz, 2H), 2.10–2.05 (m, 1H), 1.56–1.54 (m, 2H), 1.31–1.29 (m, 2H); ^13^C NMR (151 MHz, DMSO-*d*_6_) *δ*: 159.4, 156.4, 152.3, 151.2, 140.9, 129.8, 124.3, 121.8 (2C), 119.2, 115.0 (2C), 102.7, 99.2, 66.7 (2C), 55.6 (2C), 38.8, 33.9, 30.3 (2C); HRMS (ES+, *m*/*z*): found 420.2150, calcd for C_22_H_26_N_7_O_2_, [M+H]+, 420.2148.

#### 3.3.9. N-Methyl-N-(3-methylbenzyl)-6-(1-(pyridin-3-yl)-1H-1,2,3-triazol-4-yl)-7H-pyrrolo[2,3-d]pyrimidin-4-amine (**22a**)

The reaction was run as described in General Procedure B using **8a** (110 mg, 0.21 mmol). This gave 70 mg (0.17 mmol, 85%) of a white powder, mp. 275–277 °C; HPLC method A: 95%; ^1^H NMR (600 MHz, DMSO-*d*_6_) *δ*: 12.41 (s, 1H), 9.14 (d, *J* = 2.6 Hz, 1H), 9.10 (s, 1H), 8.73 (dd, *J* = 4.8, 1.4 Hz, 1H), 8.34–8.32 (m, 1H), 8.19 (s, 1H), 7.71–7.68 (s, 1H), 7.22 (t, *J* = 7.6 Hz, 1H), 7.11–7.03 (m, 4H), 5.01 (s, 2H), 3.35 (s, 3H), 2.27 (s, 3H); ^13^C NMR (151 MHz, DMSO-*d*_6_) *δ*: 156.6, 152.5, 151.5, 149.9, 141.3, 141.2, 138.2, 137.6, 133.1, 128.5, 128.1, 127.6, 127.5, 124.7, 124.2, 124.0, 119.5, 102.7, 99.5, 52.6, 37.2, 21.0; HRMS (ES+, *m*/*z*): found 397.1888, calcd for C_22_H_21_N_8_, [M+H]+, 397.1889.

#### 3.3.10. N-Methyl-6-(1-(pyridin-3-yl)-1H-1,2,3-triazol-4-yl)-N-((tetrahydro-2H-pyran-4-yl)methyl)-7H-pyrrolo[2,3-d]pyrimidin-4-amine (**22b**)

The reaction was run as described in General Procedure B using **8b** (100 mg, 0.22 mmol). This gave 60 mg (0.15 mmol, 81%) of a white powder, mp. > 300 °C; HPLC method A: 96%; ^1^H NMR (600 MHz, DMSO-*d*_6_) *δ*: 12.36 (s, 1H), 9.15 (d, *J* = 2.7 Hz, 1H), 9.12 (s, 1H), 8.74 (dd, *J* = 4.8, 1.4 Hz, 1H), 8.36–8.33 (m, 1H), 8.14 (s, 1H), 7.72–7.70 (m, 1H), 7.06 (s, 1H), 3.84 (dd, *J* = 11.5, 2.6 Hz, 2H), 3.69 (d, *J* = 7.4 Hz, 2H), 3.41 (s, 3H), 3.26 (td, *J* = 11.7, 2.1 Hz, 2H), 2.10–2.06 (m, 1H), 1.57–1.54 (m, 2H), 1.34–1.27 (m, 2H); ^13^C NMR (151 MHz, DMSO-*d*_6_) *δ*: 156.5, 152.4, 151.4, 149.9, 141.4, 141.3, 133.1, 128.1, 124.7, 123.9, 119.5, 102.7, 99.6, 66.7 (2C), 55.4, 38.9, 33.9, 30.3 (2C); HRMS (ES+, *m*/*z*): found 391.1995, calcd for C_20_H_23_N_8_O, [M+H]+, 391.1995.

#### 3.3.11. 6-(1-Benzyl-1H-1,2,3-triazol-4-yl)-N-methyl-N-(3-methylbenzyl)-7H-pyrrolo[2,3-d]pyrimidin-4-amine (**23a**)

The reaction was run as described in General Procedure B using **9a** (75 mg, 0.14 mmol). This gave 51 mg (0.12 mmol, 91%) of a white solid; mp. 288–290 °C; HPLC method A: 99%; ^1^H NMR (400 MHz, DMSO-*d*_6_) *δ*: 12.20 (s, 1H), 8.40 (s, 1H), 8.14 (s, 1H), 7.42–7.33 (m, 5H), 7.21 (t, *J* = 7.5 Hz, 1H), 7.08–7.03 (m, 3H), 6.92 (s, 1H), 5.67 (s, 2H), 4.99 (s, 2H), 3.32 (s, 3H), 2.26 (s, 3H); ^13^C NMR (101 MHz, DMSO-*d*_6_) *δ*: 156.5, 152.4, 151.1, 140.4, 138.2, 137.6, 135.8, 128.8 (2C), 128.4, 128.2, 128.0 (2C), 127.6, 127.5, 125.1, 124.0, 121.0, 102.6, 98.5, 52.9, 52.6, 37.1, 21.0; HRMS (ES+, *m*/*z*): found 410.2093, calcd for C_24_H_24_N_7_, [M+H]+, 410.2093.

#### 3.3.12. 6-(1-Benzyl-1H-1,2,3-triazol-4-yl)-N-methyl-N-((tetrahydro-2H-pyran-4-yl)methyl)-7H-pyrrolo[2,3-d]pyrimidin-4-amine (**23b**)

The reaction was run as described in General Procedure B using **9b** (70 mg, 0.13 mmol). This gave 38 mg (0.09 mmol, 78%) of a white solid, mp. 298–300 °C; HPLC method A: 99%; ^1^H NMR (400 MHz, DMSO-*d*_6_) *δ*: 12.24 (s, 1H), 8.41 (s, 1H), 8.09 (s, 1H), 7.43–7.34 (m, 5H), 6.97 (s, 1H), 5.69 (s, 2H), 3.83 (dd, *J* = 11.8, 3.5 Hz, 2H), 3.66 (d, *J* = 7.4 Hz, 2H), 3.37 (s, 3H), 3.24 (td, *J* = 11.7, 2.1 Hz, 2H), 2.08–2.02 (m, 1H), 1.55–1.51 (m, 2H), 1.33–1.23 (m, 2H); ^13^C NMR (101 MHz, DMSO-*d*_6_) *δ*: 156.4, 152.2, 151.1, 140.4, 135.8, 128.8 (2C), 128.2, 128.0 (2C), 124.7, 120.9, 102.6, 98.7, 66.7 (2C), 55.3, 52.9, 33.9, 30.2 (2C); The N-Me group is within the DMSO-*d*_5_ signal; HRMS (ES+, *m*/*z*): found 404.2196, calcd for C_22_H_26_N_7_O, [M+H]+, 404.2199.

#### 3.3.13. 6-(1-(4-Fluorobenzyl)-1H-1,2,3-triazol-4-yl)-N-methyl-N-(3-methylbenzyl)-7H-pyrrolo[2,3-d]pyrimidin-4-amine (**24a**)

The reaction was run as described in General Procedure B using **10a** (110 mg, 0.20 mmol). This gave 72 mg (0.16 mmol, 86%), mp. 274–276 °C; HPLC method A: 99%; ^1^H NMR (600 MHz, DMSO-*d*_6_) *δ*: 12.21 (s, 1H), 8.37 (s, 1H), 8.14 (s, 1H), 7.44–7.41 (m, 2H), 7.25–7.19 (m, 3H), 7.08–7.04 (m, 3H), 6.92 (s, 1H), 5.67 (s, 2H), 4.99 (s, 2H), 3.32 (s, 3H), 2.26 (s, 3H); ^13^C NMR (151 MHz, DMSO-*d*_6_) *δ*: 161.9 (d, *J* = 244.5 Hz), 156.5, 152.4, 151.2, 140.3, 138.2, 137.6, 132.0 (d, *J* = 3.0 Hz), 130.4 (d, *J* = 8.4 Hz, 2C), 128.4, 127.6, 127.5, 125.0, 124.0, 120.9, 115.7 (d, *J* = 21.7 Hz, 2C), 102.6, 98.6, 52.6, 52.1, 37.1, 21.0; HRMS (ES+, *m*/*z*): found 428.2002, calcd for C_24_H_23_FN_7_, [M+H]+, 428.1999.

#### 3.3.14. 6-(1-(4-Fluorobenzyl)-1H-1,2,3-triazol-4-yl)-N-methyl-N-((tetrahydro-2H-pyran-4-yl)methyl)-7H-pyrrolo[2,3-d]pyrimidin-4-amine (**24b**)

The reaction was run as described in General Procedure **B** using **11b** (120 mg, 0.22 mmol). This gave 76 mg (0.18 mmol, 83%) of a white powder, mp. 281–283 °C; HPLC method A: 98%; ^1^H NMR (600 MHz, DMSO-*d*_6_) *δ*: 12.15 (s, 1H), 8.40 (s, 1H), 8.10 (s, 1H), 7.45–7.43 (m, 2H), 7.25 (t, *J* = 8.9 Hz, 2H), 6.97 (s, 1H), 5.68 (s, 2H), 3.83 (dd, *J* = 11.5, 2.6 Hz, 2H), 3.66 (d, *J* = 7.4 Hz, 2H), 3.37 (s, 3H), 3.24 (td, *J* = 11.7, 2.1 Hz, 2H), 2.07–2.03 (m, 1H), 1.54–1.52 (m, 2H), 1.32–1.25 (m, 2H); ^13^C NMR (151 MHz, DMSO-*d*_6_) *δ*: 161.9 (d, *J* = 244.4 Hz), 156.4, 152.2, 151.1, 140.4, 132.0 (d, *J* = 3.2 Hz), 130.4 (d, *J* = 8.5 Hz, 2C), 124.7, 120.9, 115.7 (d, *J* = 21.6 Hz, 2C), 102.7, 98.7, 66.7 (2C), 55.3, 52.1, 38.8, 33.9, 30.2 (2C); HRMS (ES+, *m*/*z*): found 422.2102, calcd for C_22_H_25_FN_7_O, [M+H]+, 422.2105.

#### 3.3.15. N-Methyl-N-(3-methylbenzyl)-6-(1-(4-methylbenzyl)-1H-1,2,3-triazol-4-yl)-7H-pyrrolo[2,3-d]pyrimidin-4-amine (**25a**)

The reaction was run as described in General Procedure B using **11a** (120 mg, 0.22 mmol). This gave 90 mg (0.21 mmol, 99%) of a white powder, mp. 253–255 °C; HPLC method A: 98%; ^1^H NMR (600 MHz, DMSO-*d*_6_) *δ*: 12.15 (s, 1H), 8.31 (s, 1H), 8.10 (s, 1H), 7.21 (d, *J* = 8.1 Hz, 2H), 7.18–7.16 (m, 3H), 7.04 (s, 1H), 7.03–7.00 (m, 2H), 6.88 (s, 1H), 5.57 (s, 2H), 4.95 (s, 2H), 3.28 (s, 3H), 2.25 (s, 3H), 2.23 (s, 3H); ^13^C NMR (151 MHz, DMSO-*d*_6_) *δ*: 156.5, 152.4, 151.1, 140.3, 138.2, 137.6, 132.7, 129.3 (3C), 128.4, 128.1 (32), 127.6, 127.5, 125.1, 124.0, 120.8, 102.6, 98.5, 52.8, 52.6, 37.1, 21.0, 20.7; HRMS (ES+, *m*/*z*): found 424.2249, calcd for C_25_H_26_N_7_, [M+H]+, 424.2250.

#### 3.3.16. N-Methyl-6-(1-(4-methylbenzyl)-1H-1,2,3-triazol-4-yl)-N-((tetrahydro-2H-pyran-4-yl)methyl)-7H-pyrrolo[2,3-d]pyrimidin-4-amine (**25b**)

The reaction was run as described in General Procedure B using **11b** (105 mg, 0.19 mmol). This gave 67 mg (0.16 mmol, 84%) of a white powder, mp. 279–281 °C; HPLC method A: 98%; HPLC method B > 99%; ^1^H NMR (600 MHz, DMSO-*d*_6_) *δ*: 12.13 (s, 1H), 8.37 (s, 1H), 8.09 (s, 1H), 7.26 (d, *J* = 8.1 Hz, 2H), 7.21 (d, *J* = 7.7 Hz, 2H), 6.96 (s, 1H), 5.62 (s, 2H), 3.83 (dd, *J* = 11.5, 2.6 Hz, 2H), 3.66 (d, *J* = 7.4 Hz, 2H), 3.37 (s, 3H), 3.24 (td, *J* = 11.7, 2.1 Hz, 2H), 2.29 (s, 3H), 2.07–2.03 (m, 1H), 1.54–1.52 (m, 2H), 1.32–1.25 (m, 2H); ^13^C NMR (151 MHz, DMSO-*d*_6_) *δ*: 156.4, 152.2, 151.1, 140.4, 137.6, 132.7, 129.3 (2C), 128.1 (2C), 124.8, 120.8, 102.7, 98.6, 66.7 (2C), 55.3, 52.8, 38.8, 33.9, 30.2 (2C), 20.7. HRMS (ES+, *m*/*z*): found 418.2351, calcd for C_23_H_28_N_7_O, [M+H]+, 418.2355.

#### 3.3.17. 6-(1-(4-Methoxybenzyl)-1H-1,2,3-triazol-4-yl)-N-methyl-N-(3-methylbenzyl)-7H-pyrrolo[2,3-d]pyrimidin-4-amine (**26a**)

The reaction was run as described in General Procedure B using **12a** (132 mg, 0.23 mmol). This gave 70 mg (0.15 mmol, 69%) of a white solid, mp. 240–242 °C; HPLC method A: 82%; ^1^H NMR (600 MHz, DMSO-*d*_6_) *δ*: 12.18 (s, 1H), 8.33 (s, 1H), 8.14 (s, 1H), 7.33 (d, *J* = 8.6 Hz, 2H), 7.21 (t, *J* = 7.6 Hz, 1H), 7.08–7.03 (m, 3H), 6.96 (d, *J* = 8.6 Hz, 2H), 6.91 (s, 1H), 5.58 (s, 2H), 4.98 (s, 2H), 3.74 (s, 3H), 3.31 (s, 3H), 2.26 (s, 3H); ^13^C NMR (151 MHz, DMSO-*d*_6_) *δ*: 159.2, 156.5, 152.3, 151.2, 140.2, 138.2, 137.6, 129.7 (3C), 128.4, 127.6, 127.5, 125.1, 124.0, 120.7, 114.2 (2C), 102.6, 98.5, 55.1, 52.6, 52.5, 37.1, 21.0; HRMS (ES+, *m*/*z*): found 440.2197, calcd for C_25_H_26_N_7_O, [M+H]+, 440.2199.

#### 3.3.18. 6-(1-(4-Methoxybenzyl)-1H-1,2,3-triazol-4-yl)-N-methyl-N-((tetrahydro-2H-pyran-4-yl)methyl)-7H-pyrrolo[2,3-d]pyrimidin-4-amine (**26b**)

The reaction was run as described in General Procedure B using **12b** (132 mg, 0.23 mmol). This gave 89 mg (0.20 mmol, 88%) of a white solid, mp. 279–281 °C; HPLC method A: 94%; ^1^H NMR (600 MHz, DMSO-*d*_6_) *δ*: 12.13 (s, 1H), 8.35 (s, 1H), 8.09 (s, 1H), 7.34 (d, *J* = 8.7 Hz, 2H), 6.97–6.96 (m, 3H), 5.59 (s, 2H), 3.83 (dd, *J* = 11.4, 2.7 Hz, 2H), 3.75 (s, 3H), 3.66 (d, *J* = 7.4 Hz, 2H), 3.36 (s, 3H), 3.24 (td, *J* = 11.7, 2.0 Hz, 2H), 2.07–2.03 (m, 1H), 1.54–1.51 (m, 2H), 1.31–1.25 (m, 2H); ^13^C NMR (151 MHz, DMSO-*d*_6_) *δ*: 159.2, 156.4, 152.2, 151.1, 140.3, 129.7 (2C), 127.6, 124.8, 120.6, 114.2 (2C), 102.7, 98.6, 66.7 (2C), 55.1 (2C), 52.5, 38.8, 33.9, 30.2 (2C); HRMS (ES+, *m*/*z*): found 434.2310, calcd for C_23_H_28_N_7_O_2_, [M+H]+, 434.2304.

#### 3.3.19. N-Methyl-N-(3-methylbenzyl)-6-(1-(pyridin-3-ylmethyl)-1H-1,2,3-triazol-4-yl)-7H-pyrrolo[2,3-d]pyrimidin-4-amine (**27a**)

The reaction was run as described in General Procedure B using **13a** (114 mg, 0.21 mmol). This gave 60 mg (0.14 mmol, 70%) of a white solid, mp. 273–275 °C; HPLC method A: 98%; HPLC method B > 98%; ^1^H NMR (400 MHz, DMSO-*d*_6_) *δ*: 12.20 (s, 1H), 8.65 (s, 1H), 8.57 (d, *J* = 4.8 Hz, 1H), 8.43 (s, 1H), 8.14 (s, 1H), 7.76 (d, *J* = 7.9 Hz, 1H), 7.45–7.41 (m, 1H), 7.20 (t, *J* = 7.5 Hz, 1H), 7.08–7.03 (m, 3H), 6.91 (s, 1H), 5.74 (s, 2H), 4.99 (s, 2H), 3.31 (s, 3H), 2.26 (s, 3H); ^13^C NMR (101 MHz, DMSO-*d*_6_) *δ*: 156.5, 152.5, 151.1, 149.5, 149.3, 140.5, 138.2, 137.6, 135.9, 131.5, 128.4, 127.6, 127.4, 125.1, 124.0, 123.9, 121.1, 102.7, 98.6, 52.6, 50.5, 37.1, 25.1, 21.0; HRMS (ES+, *m*/*z*): found 411.2049, calcd for C_23_H_23_N_8_, [M+H]+, 411.2046.

#### 3.3.20. N-Methyl-6-(1-(pyridin-3-ylmethyl)-1H-1,2,3-triazol-4-yl)-N-((tetrahydro-2H-pyran-4-yl)methyl)-7H-pyrrolo[2,3-d]pyrimidin-4-amine (**27b**)

The reaction was run as described in General Procedure B using **13b** (100 mg, 0.19 mmol). This gave 60 mg (0.14 mmol, 80%) of a white powder, mp. 293–295 °C; HPLC method A: 98%; ^1^H NMR (400 MHz, DMSO-*d*_6_) *δ*: 12.46 (s, 1H), 8.66 (d, *J* = 2.3 Hz, 1H), 8.58 (dd, *J* = 4.8, 1.7 Hz, 1H), 8.45 (s, 1H), 8.10 (s, 1H), 7.79–7.76 (m, 1H), 7.46–7.42 (m, 1H), 6.97 (s, 1H), 5.76 (s, 2H), 3.83 (dd, *J* = 11.6, 3.1 Hz, 2H), 3.66 (d, *J* = 7.4 Hz, 2H), 3.37 (s, 3H), 3.24 (td, *J* = 11.7, 2.1 Hz, 2H), 2.08–2.02 (m, 1H), 1.55–1.52 (m, 2H), 1.33–1.23 (m, 2H); ^13^C NMR (101 MHz, DMSO-*d*_6_) *δ*: 156.4, 152.2, 151.1, 149.5, 149.3, 140.5, 135.9, 131.5, 124.6, 123.9, 121.0, 102.6, 98.8, 66.7 (2C), 55.3, 50.5, 38.8, 33.9, 30.2 (2C); HRMS (ES+, *m*/*z*): found 405.2157, calcd for C_21_H_25_N_8_O, [M+H]+, 405.2151.

#### 3.3.21. 3-(4-(4-(Methyl(3-methylbenzyl)amino)-7H-pyrrolo[2,3-d]pyrimidin-6-yl)-1H-1,2,3-triazol-1-yl)propan-1-ol (**28a**)

The reaction was run as described in General Procedure B using **14a** (100 mg, 0.20 mmol). The solvent was removed by evaporation, and the remaining residue was added to water (10 mL) and extracted with CH_2_Cl_2_ (4 × 10 mL). Purification by silica-gel flash column chromatography (CH_2_Cl_2_/MeOH 9:1, R_f_ = 0.38) gave 47 mg (0.12 mmol, 64%) of a white powder, mp. 250–252 °C; HPLC method A: 99%; ^1^H NMR (600 MHz, DMSO-*d*_6_) *δ*: 12.23 (s, 1H), 8.36 (s, 1H), 8.15 (s, 1H), 7.21 (t, *J* = 7.6 Hz, 1H), 7.09 (s, 1H), 7.06 (t, *J* = 7.6 Hz, 2H), 6.90 (s, 1H), 4.99 (s, 2H), 4.69 (t, *J* = 5.0 Hz, 1H), 4.47 (t, *J* = 7.1 Hz, 2H), 3.43 (q, *J* = 6.1 Hz, 2H), 3.32 (s, 3H), 2.27 (s, 3H), 1.99 (p, *J* = 6.5 Hz, 2H); ^13^C NMR (151 MHz, DMSO-*d*_6_) *δ*: 156.5, 152.3, 151.1, 139.9, 138.2, 137.6, 128.4, 127.6, 127.5, 125.2, 124.0, 121.0, 102.6, 98.4, 57.3, 52.6, 46.7, 37.1, 32.8, 21.0; HRMS (ES+, *m*/*z*): found 378.2044, calcd for C_20_H_24_N_7_O, [M+H]+, 378.2042.

#### 3.3.22. 3-(4-(4-(Methyl((tetrahydro-2H-pyran-4-yl)methyl)amino)-7H-pyrrolo[2,3-d]pyrimidin-6-yl)-1H-1,2,3-triazol-1-yl)propan-1-ol (**28b**)

The reaction was run as described in General Procedure B using **14b** (80 mg, 0.16 mmol). The solvent was removed by evaporation, and the remaining residue was added to water (10 mL) and extracted with CH_2_Cl_2_ (4 × 10 mL). Purification by silica-gel flash column chromatography (CH_2_Cl_2_/MeOH, 9:1, R_f_ = 0.32) gave 40 mg (0.10 mmol, 68%) of a white powder, mp. 291–293 °C; HPLC method A: 99%; ^1^H NMR (600 MHz, DMSO-*d*_6_) *δ*: 12.19 (s, 1H), 8.39 (s, 1H), 8.11 (s, 1H), 6.95 (s, 1H), 4.70 (t, *J* = 5.0 Hz, 1H), 4.48 (t, *J* = 7.1 Hz, 2H), 3.83 (dd, *J* = 11.5, 2.6 Hz, 2H), 3.67 (d, *J* = 7.4 Hz, 2H), 3.44 (q, *J* = 5.8 Hz, 2H), 3.37 (s, 3H), 3.24 (td, *J* = 11.7, 2.1 Hz, 2H), 2.08–2.04 (m, 1H), 2.00 (p, *J* = 6.6 Hz, 2H), 1.55–1.52 (m, 2H), 1.32–1.25 (m, 2H); ^13^C NMR (151 MHz, DMSO-*d*_6_) *δ*: 156.4, 152.2, 151.0, 140.0, 124.9, 121.0, 102.7, 98.5, 66.7 (2C), 57.4, 55.3, 46.8, 38.8, 33.9, 32.9, 30.3 (2C); HRMS (ES+, *m*/*z*): found 372.2147, calcd for C_18_H_26_N_7_O_2_, [M+H]+, 372.2148.

#### 3.3.23. N-Methyl-N-(3-methylbenzyl)-6-(1-(tetrahydro-2H-pyran-4-yl)-1H-1,2,3-triazol-4-yl)-7H-pyrrolo[2,3-d]pyrimidin-4-amine (**29a**)

The reaction was run as described in General Procedure B using **15a** (120 mg, 0.23 mmol). This gave 65 mg (0.16 mmol, 72%) of a white powder, mp. 273–275 °C; HPLC method A: 87%; ^1^H NMR (600 MHz, DMSO-*d*_6_) *δ*: 12.23 (s, 1H), 8.47 (s, 1H), 8.15 (s, 1H), 7.21 (t, *J* = 7.6 Hz, 1H), 7.09 (s, 1H), 7.06 (t, *J* = 7.3 Hz, 2H), 6.90 (s, 1H), 4.99 (s, 2H), 4.84–4.79 (m, 1H), 4.00–3.98 (m, 2H), 3.52 (td, *J* = 11.8, 2.1 Hz, 2H), 3.33 (s, 3H), 2.27 (s, 3H), 2.12–2.09 (m, 2H), 2.02–1.95 (m, 2H); ^13^C NMR (151 MHz, DMSO-*d*_6_) *δ*: 156.5, 152.3, 151.1, 139.8, 138.2, 137.6, 128.4, 127.6, 127.5, 125.2, 124.0, 119.3, 102.7, 98.4, 65.6 (2C), 56.2, 52.6, 37.1, 32.8 (2C), 21.0; HRMS (ES+, *m*/*z*): found 404.2197, calcd for C_22_H_26_N_7_O, [M+H]+, 404.2199.

#### 3.3.24. N-Methyl-6-(1-(tetrahydro-2H-pyran-4-yl)-1H-1,2,3-triazol-4-yl)-N-((tetrahydro-2H-pyran-4-yl)methyl)-7H-pyrrolo[2,3-d]pyrimidin-4-amine (**29b**)

The reaction was run as described in General Procedure B using **15b** (140 mg, 0.27 mmol). The solvent was removed by evaporation, and the remaining residue was added to water (10 mL) and extracted with CH_2_Cl_2_ (4 × 10 mL). Purification by silica-gel flash column chromatography (CH_2_Cl_2_/MeOH 9:1) gave 85 mg (0.21 mmol, 81%) of a white powder, mp. > 300 °C; HPLC method A: 97%; ^1^H NMR (600 MHz, DMSO-*d*_6_) *δ*: 12.18 (s, 1H), 8.50 (s, 1H), 8.11 (s, 1H), 6.95 (s, 1H), 4.85–4.80 (m, 1H), 4.00 (dd, *J* = 10.4, 4.4 Hz, 2H), 3.84 (dd, *J* = 11.5, 2.7 Hz, 2H), 3.67 (d, *J* = 7.4 Hz, 2H), 3.53 (td, *J* = 11.8, 2.1 Hz, 2H), 3.38 (s, 3H), 3.24 (td, *J* = 11.8, 2.1 Hz, 2H), 2.12 (dd, *J* = 12.4, 2.4 Hz, 2H), 2.08–2.05 (m, 1H), 2.04–1.97 (m, 2H), 1.55–1.52 (m, 2H), 1.32–1.26 (m, 2H); ^13^C NMR (151 MHz, DMSO-*d*_6_) *δ*: 156.4, 152.2, 151.1, 139.9, 124.9, 119.2, 102.7, 98.5, 66.7 (2C), 65.6 (2C), 56.2, 55.3, 38.8, 33.9, 32.8 (2C), 30.2 (2C); HRMS (ES+, *m*/*z*): found 398.2303, calcd for C_20_H_28_N_7_O_2_, [M+H]+, 398.2304.

#### 3.3.25. N-Methyl-N-(3-methylbenzyl)-6-(1-(1-methylpiperidin-4-yl)-1H-1,2,3-triazol-4-yl)-7H-pyrrolo[2,3-d]pyrimidin-4-amine (**30a**)

The reaction was run as described in General Procedure B using **16a** (120 mg, 0.22 mmol). The solvent was removed by evaporation, and the remaining residue was added to water (10 mL) and extracted with CH_2_Cl_2_ (4 × 10 mL). Purification by silica-gel flash column chromatography (CH_2_Cl_2_/MeOH 4:1, R_f_ = 0.43) gave 60 mg (0.14 mmol, 66%) of a white powder, mp. 245–247 °C; HPLC method A: 99%; ^1^H NMR (600 MHz, DMSO-*d*_6_) *δ*: 12.22 (s, 1H), 8.45 (s, 1H), 8.15 (s, 1H), 7.21 (t, *J* = 7.6 Hz, 1H), 7.09 (s, 1H), 7.06 (t, *J* = 7.5 Hz, 2H), 6.90 (s, 1H), 4.99 (s, 2H), 4.54–4.49 (m, 1H), 3.32 (s, 3H), 2.87 (d, *J* = 11.9 Hz, 2H), 2.27 (s, 3H), 2.22 (s, 3H), 2.13–2.09 (m, 4H), 2.02–1.96 (m, 2H); ^13^C NMR (151 MHz, DMSO-*d*_6_) *δ*: 157.0, 152.8, 151.6, 140.2, 138.7, 138.1, 128.9, 128.1, 128.0, 125.7, 124.5, 119.7, 103.1, 98.8, 57.5, 54.1 (2C), 53.1, 46.1, 37.6, 32.4 (2C), 21.5. HRMS (ES+, *m*/*z*): found 417.2516, calcd for C_23_H_29_N_8_, [M+H]+, 417.2515.

#### 3.3.26. N-Methyl-6-(1-(1-methylpiperidin-4-yl)-1H-1,2,3-triazol-4-yl)-N-((tetrahydro-2H-pyran-4-yl)methyl)-7H-pyrrolo[2,3-d]pyrimidin-4-amine (**30b**)

The reaction was run as described in General Procedure B using **16b** (120 mg, 0.22 mmol). The solvent was removed by evaporation, and the remaining residue was added to water (10 mL) and extracted with CH_2_Cl_2_ (4 × 10 mL). Purification by silica-gel flash column chromatography (CH_2_Cl_2_/MeOH 4:1, R_f_ = 0.36) gave 60 mg (0.14 mmol, 66%) of a white powder, mp. > 300 °C; HPLC method A: 98%; ^1^H NMR (600 MHz, DMSO-*d*_6_) *δ*: 12.25 (s, 1H), 8.47 (s, 1H), 8.10 (s, 1H), 6.94 (s, 1H), 4.54–4.50 (s, 1H), 3.84 (dd, *J* = 11.4, 2.7 Hz, 2H), 3.67 (d, *J* = 7.4 Hz, 2H), 3.38 (s, 3H), 3.25 (td, *J* = 11.7, 2.1 Hz, 2H), 2.87 (d, *J* = 12.7 Hz, 2H), 2.22 (s, 3H), 2.13–2.09 (m, 4H), 2.08–2.04 (m, 1H), 2.03–1.97 (2H), 1.55–1.53 (m, 2H), 1.33–1.26 (m, 2H); ^13^C NMR (151 MHz, DMSO-*d*_6_) *δ*: 156.4, 152.2, 151.0, 139.8, 125.0, 119.2, 102.7, 98.5, 66.7 (2C), 57.1, 55.3, 53.7 (2C), 45.6, 38.8, 33.9, 32.0 (2C), 30.2 (2C); HRMS (ES+, *m*/*z*): found 411.2616, calcd for C_21_H_31_N_8_O, [M+H]+, 411.2621.

#### 3.3.27. N-Methyl-N-(3-methylbenzyl)-6-(1-((tetrahydro-2H-pyran-4-yl)methyl)-1H-1,2,3-triazol-4-yl)-7H-pyrrolo[2,3-d]pyrimidin-4-amine (**31a**)

The reaction was run as described in General Procedure B using **17a** (90 mg, 0.16 mmol). This gave 55 mg (0.13 mmol, 81%) of a white powder, mp. 295–297 °C; HPLC method A: 98%; ^1^H NMR (600 MHz, DMSO-*d*_6_) *δ*: 12.24 (s, 1H), 8.35 (s, 1H), 8.15 (s, 1H), 7.21 (t, *J* = 7.6 Hz, 1H), 7.09 (s, 1H), 7.06 (t, *J* = 7.3 Hz, 2H), 6.90 (s, 1H), 4.99 (s, 2H), 4.34 (d, *J* = 7.1 Hz, 2H), 3.84 (dd, *J* = 11.6, 2.7 Hz, 2H), 3.33 (s, 3H), 3.26 (td, *J* = 11.6, 2.1 Hz, 2H), 2.27 (s, 3H), 2.10–2.06 (m, 1H), 1.44 (d, *J* = 12.9 Hz, 2H), 1.31–1.24 (m, 2H); ^13^C NMR (151 MHz, DMSO-*d*_6_) *δ*: 156.5, 152.4, 151.2, 139.9, 138.2, 137.6, 128.4, 127.6, 127.4, 125.1, 124.0, 121.4, 102.6, 98.4, 66.4 (2C), 54.7, 52.6, 37.1, 35.6, 29.7 (2C), 21.0; HRMS (ES+, *m*/*z*): found 404.2197, calcd for C_23_H_28_N_7_O, [M+H]+, 404.2199.

#### 3.3.28. N-Methyl-N-((tetrahydro-2H-pyran-4-yl)methyl)-6-(1-((tetrahydro-2H-pyran-4-yl)methyl)-1H-1,2,3-triazol-4-yl)-7H-pyrrolo[2,3-d]pyrimidin-4-amine (**31b**)

The reaction was run as described in General Procedure B using **17b** (120 mg, 0.22 mmol). This gave 65 mg (0.15 mmol, 71%) of a with powder, mp. 288–290 °C; HPLC method A: 99%; ^1^H NMR (600 MHz, DMSO-*d*_6_) *δ*: 12.18 (s, 1H), 8.38 (s, 1H), 8.11 (s, 1H), 6.95 (s, 1H), 4.36–4.35 (m, 2H), 3.85–3.83 (m, 4H), 3.67 (d, *J* = 7.5 Hz, 2H), 3.38 (s, 3H), 3.29–3.23 (m, 4H), 2.08 (s, 2H), 1.50 (dd, *J* = 5.0, 12.9 Hz, 4H), 1.30–1.27 (m, 4H); ^13^C NMR (151 MHz, DMSO-*d*_6_) *δ*: 156.4, 152.2, 151.1, 140.0, 124.9, 121.3, 102.7, 98.5, 66.7 (2C), 66.4 (2C), 55.3, 54.7, 38.8, 35.6, 33.9, 30.2 (2C), 29.7 (2C); HRMS (ES+, *m*/*z*): found 412.2461, calcd for C_21_H_30_N_7_O_2_, [M+H]+, 412.2461.

### 3.4. Biochemical Assays

The CSF1R enzymatic inhibitory assays, the kinase panel screen, the cell viability assay with Ba/F3 cells, solubility, microsomal stability, plasma protein binding, and the MDCK permeability assay were performed as previously described [22]. The Caco-2 assay was described by Aarhus et al. [20].

#### 3.4.1. KIT Inhibition (K_D_)

The principle behind this time-resolved FRET (TR-FRET) assay is based on the binding of a tracer containing a TR-FRET acceptor dye to the active site of cKIT kinase. It is detected by using an anti-GST antibody containing a TR-FRET donor dye, which binds to the GST tag at the N-terminus of cKIT. The binding of an inhibitor to the kinase displaces the tracer, resulting in a loss of FRET. For every sample, 2 µL of an assay mixture containing cKIT (0.9375 nM, Carna Biosciences, Kobe, Japan) and a buffer (50 mM HEPES pH 7,5, 10 mM MgCl_2_, 1 mM EGTA, 0.01% Brij35) were transferred into an assay plate (e.g., Corning #4513). Compounds were added in a concentration range of 1 µM to 0.00025 µM using an acoustic dispenser (Echo520, BeckmanCoulter, Brea, CA, USA) equipped with Echo Dose Response software version 2.4,15. After that, the kinase anti-GST antibody Eu-conjugate mix (8 µL, diluted 1/400, Revvity, Waltham, MA, USA) was added. After incubation for 30 min at 4 °C in a pre-cooled box containing wet tissue (to prevent edge effects), the reaction was started via the addition of Kinase Tracer 236 (5 µL, 4.5 nM, ThermoFisher) and mixed using a Bioshake 5000 microplate shaker (Q Instruments, Jena, Germany). After 60 min of incubation at 4 °C (pre-cooled box), the FRET signal was measured at 340 nm excitation, 665 nm, and 615 nm emission with a Pherastar FSX microplate reader (BMG Labtech, Ortenberg, Germany) with a 60 µs delay and a 200 µs integration time. *K*_D_ values were determined from the sigmoidal dose–response curves with Scigilian Analyze software version 5.8.5.15 (Scigilian, Montreal, QC, Canada).

#### 3.4.2. GIST-T1 CTG Assay

The CellTiter-Glo Luminescent Cell Viability Assay (Promega, Madison, WI, USA) is a homogeneous method of determining the number of viable cells in a culture. It is based on the quantification of ATP, indicating the presence of metabolically active cells. The GIST-T1 cell line (CosmoBio, Tokyo, Japan, # PMC-GIST01C) was cultivated in GIST-T1 cell culture medium without antibiotics (#GIST01C, CosmoBio) according to the product manual. For CellTiter-Glo Luminescent Cell Viability Assays, 25 µL of the GIST-T1 cell suspension are seeded at a concentration of 400 cells/384well plate on day 1. In previous assays, this cell number was shown to ensure assay linearity and optimal signal intensity. After incubation for 24 h at 37 °C/5% CO_2_, the compounds are added at different concentrations by Echo Liquid Handling Technology. Cells are further incubated in humidified chambers for 72 h at 37 °C and 5% CO_2_. Cells treated with the compound vehicle DMSO are used as positive controls and cells treated with 10 µM Staurosporine serve as negative controls. At day 5, 72 h after compound addition, the CellTiter Glo Reagent is prepared according to the instructions of the kit (Promega Inc.). The reagent is mixed 1:1 with the cell culture medium. Thereafter, the mixture and assay plates are equilibrated at room temperature for 20 min. Equal volumes of the reagent–medium mixture is added to the volume of the culture medium present in each well. The plates are mixed at ~300 rpm for 2 min on an orbital shaker. The microplates are then incubated at room temperature for 10 min for stabilization of the luminescent signal. Following incubation, the luminescence is recorded on a Victor microplate reader (Perkin Elmer) using a 200 ms integration time. The data are then analyzed with Excel using the XLFIT Plugin (dose–response Fit 205) for IC_50_ determination. As a quality control, the Z′-factor is calculated from 16 positive and negative control values. Only assay results showing a Z′-factor ≥ 0.5 are used for further analysis.

## 4. Conclusions

Two series of pyrrolopyrimdines decorated with substituted 1,2,3-triazoles (28 compounds) were synthesized using Sonogashira cross-coupling and copper-catalyzed azide–alkyne cycloaddition in the key steps. The CSF1R enzymatic inhibition studies showed 27 of the derivatives to have lower IC_50_ than the reference drug. Although the calculated logP is <4, several derivatives had low solubility, complicating development. Three derivatives showed promising activity in Ba/F3 cells (IC_50_: 0.3–0.6 µM) and no cellular toxicity. These derivatives have low activity toward the related PDGFR family of kinases, but profiling in a panel of kinases showed the front-runner compound **27a** to also have high activity toward ABL1, ABL2, EPHA2, SRC, and YES. ADME profiling revealed the compounds to be unstable in phase I metabolic studies using both human and liver microsomes. These triazoles can be a starting point for the design of a new multi-kinase inhibitor if measures are taken to identify and block the metabolic labile site.

## Figures and Tables

**Table 1:** Enzymatic and Ba/F3 IC_50_ and solubility of aryl-substituted derivatives (A-series).

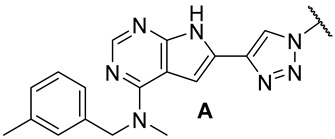							
Group	Comp.	cLogP ^a^	PSA ^b^	Sol. ^c^	Enz. IC_50_ (nM) ^d^	CSF1R Ba/F3 IC_50_ (µM) ^e^	IL-3 Ba/F3 IC_50_ (µM) ^f^
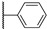	**18a**	3.08	75.5	0.3	1.9 (0.94)	>10	>10
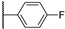	**19a**	3.18	75.5	0.4	7.7 (0.97)	>10	>10
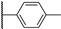	**20a**	3.43	75.5	3	28.2 (0.98)	>10	>10
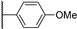	**21a**	3.01	84.8	1.3	5.9 (0.96)	6.0 ± 0.0	>10
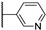	**22a**	2.08	88.4	11	2.3 (0.95)	1.2 ± 0.2	>10
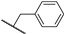	**23a**	3.43	75.5	0.3	2.0 (0.93)	>10	>10
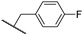	**24a**	3.44	75.5	0.2	2.0 (0.97)	>10	>10
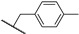	**25a**	3.68	75.5	1.1	6.3 (0.98)	>10	>10
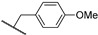	**26a**	3.27	84.8	4.6	5.8 (0.92)	2.8 ± 0.3	>10
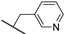	**27a**	2.33	88.4	3.1	1 < (0.98)	0.3 ± 0.0	>10
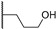	**28a**	1.76	95.8	5.5	1 < (0.85)	1.0 ± 0.1	>10
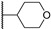	**29a**	2.66	84.8	20	1 < (0.99)	>10	>10
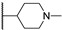	**30a**	2.77	78.8	51	1 < (0.99)	1.1 ± 0.1	>10
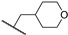	**31a**	2.41	84.8	2.5	1 < (0.98)	2.7 ± 1.0	>10
PLX-3397		3.72	66.5	20	21 (0.97)	0.06 ± 0.1	>10

(a) Calculated logP estimated using DataWarrior, Version 5.5.0. (b) Topological polar surface area (TPSA) was estimated using DataWarrior, Version 5.5.0. (Erlich approach). (c) Kinetic solubility (µM) determined spectrophotometrically at pH 7.4 in HEPES buffer. (d) Enzymatic IC_50_ values obtained by single 8-point titrations using TR-FRET-based LANCE Ultra assay (Perkin Elmer, Shelton, CT, USA), ATP concentration: 25 µM, goodness of fit (R^2^) for the regression is shown in parentheses. (e) Cellular assay of Ba/F3 cells engineered to be dependent on CSF1R, duplicate measurements. (f) Control assay for cellular toxicity Ba/F3 cells dependent on IL-3, duplicate measurements.

**Table 2:** Enzymatic and Ba/F3 IC_50_ and solubility of THP-substituted derivatives (Series B).

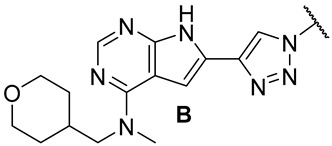							
Group	Comp.	cLogP ^a^	PSA ^b^	Sol. ^c^	Enz. IC_50_ (nM) ^d^	CSF1R Ba/F3 IC_50_ (µM) ^e^	IL-3 Ba/F3 IC_50_ (µM) ^f^
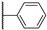	**18b**	1.91	84.8	14	1.1 (0.99)	>10	>10
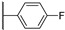	**19b**	2.01	84.8	11	2.1 (0.97)	>10	>10
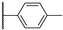	**20b**	2.25	84.8	0.7	9.1 (0.99)	3.2 ± 0.9	>10
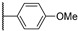	**21b**	1.84	94.0	7.3	3 (0.95)	0.6 ± 0.2	>10
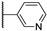	**22b**	0.91	97.6	37	2.1 (0.97)	1.6 ± 0.3	>10
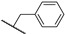	**23b**	2.16	84.8	4.9	1 < (0.95)	3.7 ± 1.9	>10
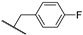	**24b**	2.26	84.8	1.7	1.5 (0.96)	4.6 ± 2.7	>10
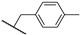	**25b**	2.50	84.8	10.6	1 < (0.96)	0.5 ± 0.0	>10
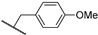	**26b**	2.09	94.0	7.2	1 < (0.98)	1.5 ± 0.5	>10
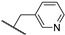	**27b**	1.16	97.6	43	1.8 (0.98)	1.7 ± 0.1	>10
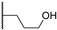	**28b**	0.59	105.0	495	1.1 (0.93)	>10	>10
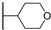	**29b**	1.48	94.0	76	1 < (0.92)	>10	>10
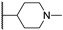	**30b**	1.59	88.0	42	1 < (0.98)	2.4 ± 0.5	>10
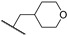	**31b**	1.24	94.0	45	1 < (0.96)	1.0 ± 0.2	>10
PLX-3397		3.72	66.5	20	21 (0.97)	0.06 ± 0.5	>10

(a) Calculated logP estimated using DataWarrior, Version 5.5.0. (b) Topological polar surface area (TPSA) was estimated using DataWarrior, Version 5.5.0. (Erlich approach). (c) Kinetic solubility (µM) determined spectrophotometrically at pH 7.4 in HEPES buffer. (d) Enzymatic IC_50_ values obtained by single 8-point titrations using TR-FRET-based LANCE Ultra assay (Perkin Elmer, Shelton, Connecticut, USA), ATP concentration: 25 µM, goodness of fit (R^2^) for the regression is shown in parentheses. (e) Cellular assay of Ba/F3 cells engineered to be dependent on CSF1R. (f) Control assay for cellular toxicity Ba/F3 cells dependent on IL-3.

**Table 3:** Addition enzymatic, cellular, and ADME profiling of **21b**, **25b,** and **27a** compared with that of structures **III**, **IV,** and PLX-3397.

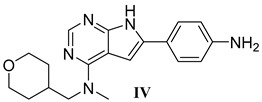						
Assay	21b	25b	27a	III	IV	PLX-3397
CSF1R enz. IC_50_ (nM) ^a^	1.2 ± 0.3	1.3 ± 0.0	0.4 ± 0.2	0.5 ± 0.2	0.3 ± 1	4.7 ± 0.5
KIT enz. *K*_D_ (nM) ^b^	>1000	>100	793 (0.93)	>3000	ND	28 (0.99)
KIT cell IC_50_ (µM) ^c^	>30	>3	>30	ND	ND	0.03
HLM [µL/min/mg] ^d^	74.5	62.4	53.7	15.5	13.6	11.8
MLM [µL/min/mg] ^e^	288	1980	295	40.7	22.0	41.4
P_app_ A > B [10^−6^ cm/s] ^f^	10.7	10.0	1.27	0.69 ^h^	37.9	4.45
P_app_ B > A [10^−6^ cm/s] ^f^	13.6	25.4	3.18	0.19 ^h^	27.3	1.49
Ratio P_app_ B > A/P_app_ A > B ^f^	1.3	2.6	2.5	0.28 ^h^	0.72	0.33
PPB (%) ^g^	76	97.5	>99	94.4	76.4	>99

(a) Enzymatic IC_50_ values obtained by 10-point titrations in duplicates (20 data points) using Z-LYTE assay platform (ThermoFisher, Waltham, MA, USA). ATP concentration is equal to *K*_M_ ca. 10 µM. (b) *K*_D_ was measured by a time-resolved FRET assay using kinase tracer 236 and an anti-GST antibody Eu-conjugate. Goodness of fit (R^2^) for the regression is shown in parentheses for those measurements showing a reasonably low value. (c) Cellular proliferation study using the Kit-positive cell line GIST-T1. (d) HLM*:* In vitro intrinsic clearance of compounds in human liver microsome (µL/min/mg)—Phase I. (e) MLM: in vitro intrinsic clearance of compounds in mouse liver microsome (µL/min/mg)—Phase I. (f) Apparent permeability coefficient (P*_app_*) measured by cell monolayer of Madin Darby canine kidney (MDCK) cells in the A-B and B-A directions. (g) Mouse plasma protein binding, fraction bound. (h) Caco-2 cell assay was used. ND-not detected.

**Table 4:** Comparison of docking scores (Kcal/mol) for **21b**, **25b**, **27a**, structure **III**, and PLX-3397, and docking toward two different CSF1R X-ray structures.

Compound	CSF1R Structure: 8CGC	CSF1R Structure: 4R7H
Structure **III**	−11.569	−9.565
PLX3397	−7.763	−12.482
**21b**	−9.848	−7.879
**25b**	−9.648	−8.982
**27a**	−10.835	−10.774

## Data Availability

Experimental data can be found in the Supplementary Materials.

## Supplementary Materials

The following supporting information can be downloaded at: https://www.mdpi.com/article/10.3390/molecules30122641/s1, This file contains information on synthesis and characterization of intermediates, enzymatic inhibition titration curves, kinase profiling, HPLC trace, NMR and HRMS spectra, and additional illustrations of modeling. Figure S1–S84: ^1^H and ^13^C NMR spectra and HRMS of compounds **18a**-**31b**; Figure S85–S90: graphics illustrating docking of **27a** and PLX-3397; Table S1: Profiling of compound **27a** towards a panel of kinases; Table S2: Examples of IC_50_ curves.
