# Supplementary material for: Evaluating Triazole-Substituted Pyrrolopyrimidines as CSF1R Inhibitors
Source: Molecules. 2025 Jun 18;30(12):2641. doi: 10.3390/molecules30122641 (PMC12196347; doi:10.3390/molecules30122641)
Supplement: Supplementary file 1 [file molecules-30-02641-s001.zip › molecules-3693610-supplementary.pdf]

Supplementary File

Evaluating Triazole-Substituted Pyrrolopyrimidines as CSF1R Inhibitors

Srinivasulu Cherukupalli 1, Jan Eickhoff 2, Carsten Degenhart 2, Peter Habenberger 2, Anke Unger 2,

Bård Helge Hoff 1,* and Eirik Sundby 3,*

1 Department of Chemistry, Norwegian University of Science and Technology (NTNU), Høgskoleringen 5, NO-7491 Trondheim, Norway; srinivasulu.cherukupalli@ntnu.no

2 Lead Discovery Center GmbH (LDC), Otto-Hahn-Strasse 15, 44227 Dortmund, Germany;

eickhoff@lead-discovery.de (J.E.); degenhart@lead-discovery.de (C.D.);

habenberger@lead-discovery.de (P.H.); unger@lead-discovery.de (A.U.)

3 Department of Material Science, Norwegian University of Science and Technology (NTNU),

NO-7491 Trondheim, Norway

* Correspondence: bard.h.hoff@ntnu.no (B.H.H.); eirik.sundby@ntnu.no (E.S.)

Contents

1. Synthesis of intermediates 2
2. Profiling of 27a towards a kinase panel 19
3. IC_{50} curves 21
4. Spectral data for the inhibitor candidates 18a-31b 23
5. Molecular docking 79

1. Synthesis of intermediates

N-Methyl-N-(3-methylbenzyl)-7-((2-(trimethylsilyl)ethoxy)methyl)-6-((trimethylsilyl) ethynyl)-7H-pyrrolo[2,3-d]pyrimidin-4-amine (2a)

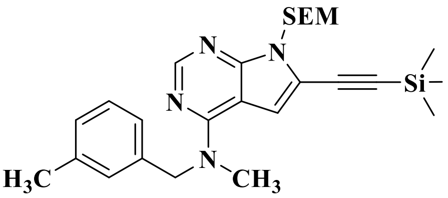

To a solution of toluene (40 mL), was added 6-iodo-*N*-methyl-*N*-(3-methylbenzyl)-7-((2-(trimethylsilyl)ethoxy) methyl)-7*H*-pyrrolo[2,3-*d*]pyrimidin-4-amine (**1a**; 2.00 g, 3.93 mmol), ethynyltrimethylsilane (2.7 mL, 19.7 mmol), copper iodide (300 mg, 1.57 mmol), triethylamine (1.6 mL, 11.8 mmol) and the mixture was degassed for 20 min. Then, $Pd(PPh_3)_4$ (220 mg, 0.20 mmol) was added to the reaction mixture and allowed to stir at rt for 2 h. The solvent was evaporated, and the residue was added to water and extracted with CH_2Cl_2 (3 × 50 mL). The combined organic layers were dried with anhydrous Na_2SO_4 and the crude was purified by silica-gel column chromatography (CH_2Cl_2/EtOAc, 4:1) to give 1.64 g (3.42 mmol, 87%) as brown viscous liquid. ^{1}H NMR (400 MHz, DMSO-d_6) δ: 8.24 (s, 1H), 7.20 (t, J = 7.7 Hz, 1H), 7.06 (d, J = 7.8 Hz, 2H), 7.01 (d, J = 7.5 Hz, 2H), 5.52 (s, 2H), 4.97 (s, 2H), 3.57 (t, J = 7.9 Hz, 2H), 3.29 (s, 3H), 2.26 (s, 3H), 0.82 (t, J = 7.8 Hz, 2H), 0.24 (s, 9H), -0.08 (s, 9H); ^{13}C NMR (101 MHz, DMSO-d_6) δ: 156.6, 152.8, 151.5, 137.7, 137.6, 128.4, 127.6, 127.4, 123.9, 116.7, 109.5, 101.4, 101.3, 95.6, 70.8, 65.8, 52.7, 37.4, 21.0, 17.2, -0.3 (3C), -1.3 (3C).

N-Methyl-N-((tetrahydro-2H-pyran-4-yl)methyl)-7-((2-(trimethylsilyl)ethoxy)methyl)-6-((trimethylsilyl)ethynyl)-7H-pyrrolo[2,3-d]pyrimidin-4-amine (2b)

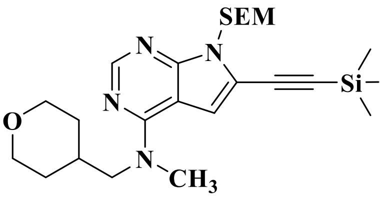

Compound **2b** was prepared as described for **2a**, but starting with 6-iodo-*N*-methyl-*N*-((tetrahydro-2*H*-pyran-4-yl)methyl)-7-((2-(trimethylsilyl)ethoxy)methyl)-7*H*-pyrrolo[2,3-*d*]pyrimidin-4-amine (**1b**, 2.00 g, 3.98 mmol). Purification by silica-gel column chromatography (CH_2Cl_2)/EtOAc, 3:2) gave 1.37 g (2.89 mmol, 73%) as a brown viscous liquid; ^{1}H NMR (400 MHz, DMSO-d_6) δ: 8.19 (s, 1H), 7.11 (s, 1H), 5.50 (s, 2H), 3.82 (dd, J = 11.7, 3.4 Hz, 2H), 3.66 (d, J = 7.4 Hz, 2H), 3.56 (t, J = 7.9 Hz, 2H), 3.33 (s, 3H), 3.23 (td, J = 11.6, 2.0 Hz, 2H), 2.05-1.99 (m, 1H), 1.49 (d, J = 11.5 Hz, 2H), 1.28 (tt, J = 12.1, 6.1 Hz, 2H), 0.81 (t, J = 7.8 Hz, 2H), 0.26 (s, 9H), -0.08 (s, 9H); ^{13}C NMR (101 MHz,

DMSO-d_6) δ: 156.5, 152.6, 151.4, 116.5, 109.7, 101.4, 101.1, 95.8, 70.7, 66.7 (3C), 65.7, 55.4, 38.9, 33.7, 30.1 (2C), 17.2, -0.3 (3C), -1.3 (3C).

6-Ethynyl-*N*-methyl-*N*-(3-methylbenzyl)-7-((2-(trimethylsilyl)ethoxy)methyl)-7*H*-pyrrolo[2,3-*d*]pyrimidin-4-amine (3a)

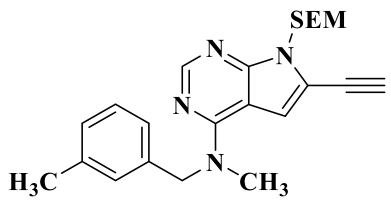

To reaction flask containing MeOH (30 mL), *N*-methyl-*N*-(3-methylbenzyl)-7-((2-(trimethylsilyl) ethoxy)methyl)-6-((trimethylsilyl)ethynyl)-7*H*-pyrrolo[2,3-*d*]pyrimidin-4-amine (**2a**, 1.64 g, 3.43 mmol) and K_2CO_3 (1.42 g, 10.28 mmol) were added, and the mixture was stirred at rt for 60 min. The solvent was evaporated, and the residue was diluted with water (30 mL) and extracted with CH_2Cl_2 (3 × 30 mL). The combined organic layers were dried with anhydrous Na_2SO_4 and concentrated to give 1.17 g (2.87 mmol, 84%) of the product as brown viscous liquid; ^{1}H NMR (400 MHz, DMSO-d_6) δ: 8.25 (s, 1H), 7.20 (t, *J* = 7.5 Hz, 1H), 7.06 (d, *J* = 11.2 Hz, 2H), 7.01 (d, *J* = 7.6 Hz, 2H), 5.53 (s, 2H), 4.98 (s, 2H), 4.71 (s, 1H), 3.56 (t, *J* = 5.2 Hz, 2H), 3.31 (s, 3H), 2.26 (s, 3H), 0.82 (t, *J* = 5.3 Hz, 2H), -0.09 (s, 9H); ^{13}C NMR (101 MHz, DMSO-d_6) δ: 171.9, 156.5, 152.7, 151.5, 137.8, 137.6, 128.4, 127.6, 127.4, 123.9, 116.2, 109.6, 101.2, 87.1, 74.5, 70.5, 65.7, 37.4, 21.0, 17.1, -1.4 (3C).

6-Ethynyl-*N*-methyl-*N*-((tetrahydro-2*H*-pyran-4-yl)methyl)-7-((2-(trimethylsilyl) ethoxy) methyl)-7*H*-pyrrolo[2,3-*d*]pyrimidin-4-amine (3b)

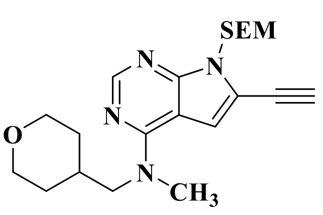

Compound **3b** was prepared as described for **3a** but starting with N-methyl-*N*-((tetrahydro-2*H*-pyran-4-yl)methyl)-7-((2-(trimethylsilyl)ethoxy)methyl)-6-((trimethylsilyl)ethynyl)-7H-pyrrolo[2,3-d]pyrimidin-4-amine (**2b**; 1.37 g, 2.90 mmol). Purification by silica-gel column chromatography (CH_2Cl_2/EtOAc, 3:2) gave 0.99 g (2.47 mmol, 85%); ^{1}H NMR (400 MHz, DMSO-d_6) δ: 8.20 (s, 1H), 7.09 (s, 1H), 5.52 (s, 2H), 4.72 (s, 1H), 3.84-3.80 (m, 2H), 3.66 (d, *J* = 7.4 Hz, 2H), 3.55 (t, *J* = 7.9 Hz, 2H), 3.34 (s, 3H), 3.26-3.20 (m, 2H), 2.05-2.00 (m, 1H), 1.52-1.48 (m, 2H), 1.32-1.22 (m, 2H), 0.81 (t, *J* = 7.8 Hz, 2H), -0.10 (s, 9H); ^{13}C NMR (101 MHz, DMSO-d_6) δ: 156.4, 152.6, 151.4, 116.0, 109.7, 101.3, 87.0, 74.7, 70.5, 66.7 (2C), 65.6, 55.4, 39.0, 33.7, 30.1 (2C), 17.1, -1.4 (3C).

General procedure A: Synthesis of triazole intermediates

To a round bottom flask containing *t*-BuOH/H_2O (3:1 by vol, 8 mL) was added the ethynyl compound (**3a** or **3b**, 0.25 mmol), the azide (1.3 equiv.), $CuSO_4$ pentahydrate (0.1 equiv.) and sodium ascorbate (0.2 equiv.). The reaction mixture was stirred at rt overnight. Upon reaction completion, the solvent was removed by evaporation. The residue diluted with water and the mixture was extracted with CH_2Cl_2. The combined organic layers were washed with brine, dried over Na_2SO_4, and concentrated. The crude product was purified by silica-gel column chromatography to achieve the desired products as stated for each specific compound.

***N*-Methyl-*N*-(3-methylbenzyl)-6-(1-phenyl-1*H*-1,2,3-triazol-4-yl)-7-((2-(trimethylsilyl) ethoxy)methyl)-7*H*-pyrrolo[2,3-*d*]pyrimidin-4-amine (4a)**

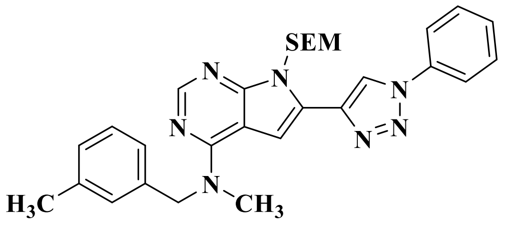

The compound was prepared as described in General Procedure A starting with **3a** (100 mg, 0.246 mmol) and azidobenzene (0.5 M in *tert*-butyl methyl ether, 0.64 mL, 0.32 mmol). Purification by silica-gel flash column chromatography (EtOAc/CH_2Cl_2/*n*-pentane, 2:1:7) gave 79 mg (0.150 mmol, 61%) of a pale-yellow viscous liquid. ^{1}H NMR (400 MHz, DMSO-*d*$_6$) δ: 9.09 (s, 1H), 8.27 (s, 1H), 7.94 (d, J = 7.3 Hz, 2H), 7.65 (t, J = 7.9 Hz, 2H), 7.53 (t, J = 7.4 Hz, 1H), 7.22 (t, J = 7.5 Hz, 1H), 7.16 (s, 1H), 7.10-7.05 (m, 3H), 5.98 (s, 2H), 5.03 (s, 2H), 3.48 (t, J = 7.9 Hz, 2H), 3.37 (s, 3H), 2.27 (s, 3H), 0.77 (t, J = 7.9 Hz, 2H), -0.19 (s, 9H); ^{13}C NMR (101 MHz, DMSO-*d*$_6$) δ: 156.6, 153.0, 151.6, 140.0, 138.0, 137.6, 136.3, 130.0 (2C), 128.9, 128.4, 127.6, 127.5, 125.6, 124.0, 120.8, 120.2 (2C), 102.5, 101.9, 70.3, 65.2, 52.7, 37.2, 21.0, 17.0, -1.5 (3C).

***N*-Methyl-6-(1-phenyl-1*H*-1,2,3-triazol-4-yl)-*N*-((tetrahydro-2*H*-pyran-4-yl)methyl)-7-((2-(trimethylsilyl)ethoxy)methyl)-7*H*-pyrrolo[2,3-*d*]pyrimidin-4-amine (4b)**

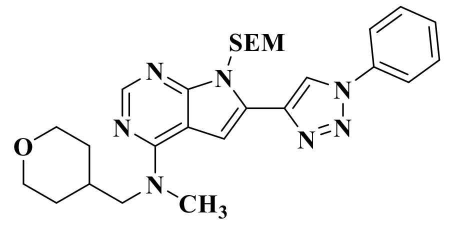

The compound was synthesized according to General Procedure A using **3b** (100 mg, 0.246 mmol) and azidobenzene (0.5 M in *tert*-butyl methyl ether 0.64 mL, 0.32 mmol). Purification by silica-gel flash column chromatography (EtOAc/*n*-pentane, 1:1) gave 104 mg

(0.200 mmol, 81%) of a brown viscous liquid. ^{1}H NMR (600 MHz, DMSO-d_6) δ: 9.11 (s, 1H), 8.22 (s, 1H), 7.95 (d, J = 7.4 Hz, 2H), 7.67-7.64 (m, 2H), 7.56-7.53 (m, 1H), 7.18 (s, 1H), 5.97 (s, 2H), 3.85-3.82 (m, 2H), 3.71 (d, J = 7.4 Hz, 2H), 3.47 (t, J = 8.0 Hz, 2H), 3.42 (s, 3H), 3.27-3.23 (m, 2H), 2.10-2.06 (m, 1H), 1.54 (d, J = 12.7 Hz, 2H), 1.34-1.27 (m, 2H), 0.76 (t, J = 8.0 Hz, 2H), -0.19 (s, 9H); ^{13}C NMR (151 MHz, DMSO-d_6) δ: 156.5, 152.9, 151.5, 140.1, 136.4, 130.0 (2C), 128.9, 125.3, 120.8, 120.2 (2C), 102.7, 101.9, 70.2, 66.7 (2C), 65.1, 55.4, 38.9, 33.8, 30.2 (2C), 17.0, -1.5 (3C).

6-(1-(4-Fluorophenyl)-1*H*-1,2,3-triazol-4-yl)-*N*-methyl-*N*-(3-methylbenzyl)-7-((2-(trimethylsilyl)ethoxy)methyl)-7*H*-pyrrolo[2,3-*d*]pyrimidin-4-amine (5a)

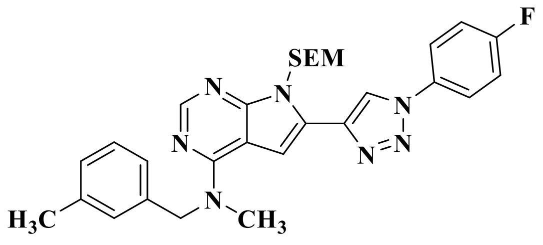

The compound was synthesized according to General Procedure A using **3a** (150 mg, 0.369 mmol) and 1-azido-4-fluorobenzene (0.5 M *tert*-butyl methyl ether, 0.96 mL, 0.48 mmol). Purification by silica-gel flash column chromatography (EtOAc/*n*-pentane, 3:7) gave 138 mg (0.254 mmol, 69%) of a brown solid. ^{1}H NMR (600 MHz, DMSO-d_6) δ: 9.07 (s, 1H), 8.26 (s, 1H), 8.00-7.97 (m, 2H), 7.50 (t, J = 8.8 Hz, 2H), 7.21 (t, J = 7.6 Hz, 1H), 7.14 (s, 1H), 7.09 (s, 1H), 7.06 (t, J = 6.9 Hz, 2H), 5.97 (s, 2H), 5.03 (s, 2H), 3.48 (t, J = 8.0 Hz, 2H), 3.36 (s, 3H), 2.26 (s, 3H), 0.77 (t, J = 8.0 Hz, 2H), -0.19 (s, 9H); ^{13}C NMR (151 MHz, DMSO-d6) δ: 161.7 (d, J = 246.2 Hz), 156.6, 153.0, 151.6, 140.0, 137.9, 137.6, 132.9 (d, J = 2.8 Hz), 128.4, 127.6, 127.5, 125.5, 124.0, 122.5 (d, J = 8.8 Hz, 2C), 121.1, 116.8 (d, J = 23.3 Hz, 2C), 102.5, 101.9, 70.2, 65.2, 52.7, 37.2, 21.0, 17.0, -1.55 (3C).

6-(1-(4-Fluorophenyl)-1*H*-1,2,3-triazol-4-yl)-*N*-methyl-*N*-((tetrahydro-2*H*-pyran-4-yl)methyl)-7-((2-(trimethylsilyl)ethoxy)methyl)-7*H*-pyrrolo[2,3-*d*]pyrimidin-4-amine (5b)

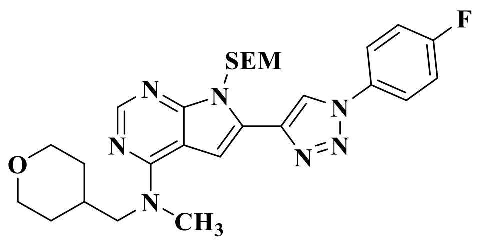

The compound was synthesized according to General Procedure A using **3b** (150 mg, 0.374 mmol) and 1-azido-4-fluorobenzene (in 0.5 M in *tert*-butyl methyl ether, 0.98 mL, 0.49 mmol). Purification by silica-gel flash column chromatography (EtOAc/*n*-pentane, 7:3) gave 138 mg (0.257 mmol, 69%) of a brown viscous liquid. ^{1}H NMR (600 MHz, DMSO-d_6) δ: 9.09 (s, 1H), 8.22 (s, 1H), 8.01-7.99 (m, 2H), 7.51 (t, J = 8.7 Hz, 2H), 7.17 (s, 1H), 5.96 (s, 2H), 3.85-3.82 (m, 2H), 3.71 (d, J = 7.5 Hz, 2H), 3.46 (t, J = 8.0 Hz, 2H), 3.41 (s, 3H), 3.25 (t, J = 10.8 Hz, 2H), 2.10-2.05 (m, 1H), 1.54 (d, J = 13.4 Hz, 2H), 1.33-1.28 (m, 2H), 0.75 (t, J = 8.0 Hz, 2H), -0.20 (s, 9H); ^{13}C NMR (151 MHz, DMSO-d_6) δ: 161.8 (d, J = 246.6 Hz), 156.5, 152.9, 151.5, 140.1, 132.9, (d, J = 3.1 Hz), 125.3, 122.6, (d, J = 8.8 Hz, 2C), 121.1, 116.8 (d, J = 23.1 Hz, 2C), 102.7, 101.9, 70.2, 66.7 (2C), 65.1, 55.4, 38.9, 33.8, 30.2 (2C), 17.0, -1.5 (3C).

***N*-Methyl-*N*-(3-methylbenzyl)-6-(1-(p-tolyl)-1*H*-1,2,3-triazol-4-yl)-7-((2-(trimethylsilyl) ethoxy)methyl)-7*H*-pyrrolo[2,3-*d*]pyrimidin-4-amine (6a)**

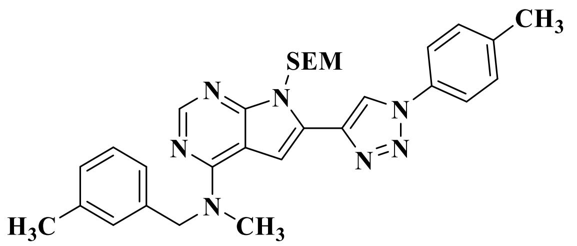

The compound was prepared as described in General Procedure A starting with **3a** (150 mg, 0.369 mmol) and 1-azido-4-methylbenzene (64 mg, 0.48 mmol). Purification by silica-gel flash column chromatography (EtOAc/*n*-pentane, 3:7) gave 123 mg, (0.228 mmol, 62%) of a brown solid. ^{1}H NMR (400 MHz, DMSO-d_6) δ: 9.04 (s, 1H), 8.26 (s, 1H), 7.81 (d, J = 8.4 Hz, 2H), 7.44 (d, J = 7.8 Hz, 2H), 7.22 (t, J = 7.5 Hz, 1H), 7.14 (s, 1H), 7.09-7.05 (m, 3H), 5.98 (s, 2H), 5.03 (s, 2H), 3.48 (t, J = 7.9 Hz, 2H), 3.37 (s, 3H), 2.40 (s, 3H), 2.27 (s, 3H), 0.77 (t, J = 8.0 Hz, 2H), -0.19 (s, 9H); ^{13}C NMR (101 MHz, DMSO-d_6) δ: 156.6, 153.0, 151.6, 139.8, 138.6, 138.0, 137.6, 134.1, 130.3 (2C), 128.4, 127.6, 127.5, 125.7, 124.0, 120.7, 120.0 (2C), 102.5, 101.9, 70.2, 65.2, 52.7, 37.2, 21.0, 20.5, 17.0, -1.5 (3C).

_N_-Methyl-_N_-((tetrahydro-2_H_-pyran-4-yl)methyl)-6-(1-(p-tolyl)-1_H_-1,2,3-triazol-4-yl)-7-((2-(trimethylsilyl)ethoxy)methyl)-7_H_-pyrrolo[2,3-_d_]pyrimidin-4-amine (6b)

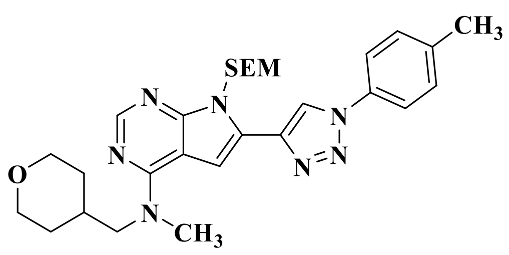

The compound was prepared as described in General Procedure A starting with **3b** (150 mg, 0.374 mmol) and 1-azido-4-methylbenzene (64 mg, 0.48 mmol). Purification by silica-gel flash column chromatography (EtOAc/*n*-pentane, 7:3) gave 118 mg, (0.221 mmol, 59%) of a brown viscous liquid. ^{1}H NMR (600 MHz, DMSO-d_6) δ: 9.07 (s, 1H), 8.22 (s, 1H), 7.83 (d, J = 8.4 Hz, 2H), 7.45 (d, J = 8.1 Hz, 2H), 7.18 (s, 1H), 5.97 (s, 2H), 3.84 (dd, J = 11.4, 2.9 Hz, 2H), 3.71 (d, J = 7.4 Hz, 2H), 3.46 (t, J = 8.0 Hz, 2H), 3.42 (s, 3H), 3.25 (t, J = 10.7 Hz, 2H), 2.41 (s, 3H), 2.10-2.06 (m, 1H), 1.54 (d, J = 10.9 Hz, 2H), 1.34-1.27 (m, 2H), 0.76 (t, J = 8.0 Hz, 2H), -0.19 (s, 9H); ^{13}C NMR (151 MHz, DMSO-d_6) δ: 156.4, 152.8, 151.3, 139.9, 138.6, 134.1, 130.3 (2C), 125.5, 120.7, 120.0 (2C), 102.6, 101.9, 70.2, 66.7 (2C), 65.1, 55.5, 38.9, 33.8, 30.2 (2C), 20.6, 17.0, -1.5 (3C).

6-(1-(4-Methoxyphenyl)-1_H_-1,2,3-triazol-4-yl)-_N_-methyl-_N_-(3-methylbenzyl)-7-((2-(trimethylsilyl)ethoxy)methyl)-7_H_-pyrrolo[2,3-_d_]pyrimidin-4-amine (7a)

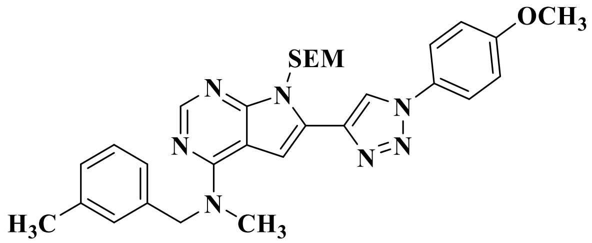

The compound was synthesized according to General Procedure A starting with **3a** (150 mg, 0.369 mmol) and 1-azido-4-methoxybenzene (0.96 mL, 0.48 mmol). Purification by silica-gel flash column chromatography (EtOAc/*n*-pentane, 2:3) gave 85 mg (0.153 mmol, 41%) of a brown viscous liquid. ^{1}H NMR (600 MHz, DMSO-d_6) δ: 8.98 (s, 1H), 8.26 (s, 1H), 7.84 (d, J = 9.0 Hz, 2H), 7.21 (t, J = 7.6 Hz, 1H), 7.18 (d, J = 9.0 Hz, 2H), 7.13 (s, 1H), 7.09 (s, 1H), 7.06 (t, J = 7.5 Hz, 2H), 5.97 (s, 2H), 5.03 (s, 2H), 3.85 (s, 3H), 3.48 (t, J = 7.9 Hz, 2H), 3.36 (s, 3H), 2.27 (s, 3H), 0.77 (t, J = 7.9 Hz, 2H), -0.18 (s, 9H); ^{13}C NMR (151 MHz, DMSO-d_6) δ: 159.4, 156.5, 153.0, 151.6, 139.7, 138.0, 137.6, 129.7, 128.4, 127.6, 127.5, 125.8, 124.0, 121.8 (2C), 120.9, 114.9 (2C), 102.4, 101.9, 70.2, 65.2, 55.6, 52.7, 37.2, 21.0, 17.0, -1.5 (3C).

6-(1-(4-Methoxyphenyl)-1*H*-1,2,3-triazol-4-yl)-*N*-methyl-*N*-((tetrahydro-2*H*-pyran-4-yl)methyl)-7-((2-(trimethylsilyl)ethoxy)methyl)-7*H*-pyrrolo[2,3-*d*]pyrimidin-4-amine (7b)

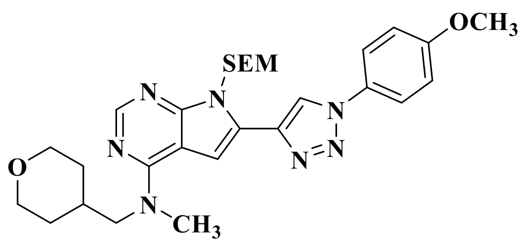

The compound was synthesized according to General Procedure A using **3b** (150 mg, 0.374 mmol) and 1-azido-4-methoxybenzene (0.98 mL, 0.49 mmol). Purification by silica-gel flash column chromatography (EtOAc/*n*-pentane, 4:1) gave 83 mg (0.151 mmol, 40%) of a brown viscous liquid. ^{1}H NMR (600 MHz, DMSO-d_6) δ: 9.0 (s, 1H), 8.22 (s, 1H), 7.85 (d, J = 9.0 Hz, 2H), 7.19 (d, J = 9.0 Hz, 2H), 7.16 (s, 1H), 5.96 (s, 2H), 3.85 (s, 3H), 3.82 (dd, J = 4.3, 1.8 Hz, 2H), 3.70 (d, J = 7.4 Hz, 2H), 3.46 (d, J = 7.9 Hz, 2H), 3.41 (s, 3H), 3.27-3.23 (m, 2H), 2.09-2.03 (m, 1H), 1.54 (d, J = 12.5 Hz, 2H), 1.33-1.26 (m, 2H), 0.75 (t, J = 7.9 Hz, 2H), -0.19 (s, 9H); ^{13}C NMR (151 MHz, DMSO-d_6) δ: 159.4, 156.4, 152.8, 151.4, 139.9, 129.8, 125.5, 121.8 (2C), 120.8, 114.9 (2C), 102.5, 102.0, 70.2, 66.7 (2C), 65.1, 55.6 (2C), 38.8, 33.8, 30.2 (2C), 17.0, -1.5 (3C).

***N*-Methyl-*N*-(3-methylbenzyl)-6-(1-(pyridin-3-yl)-1*H*-1,2,3-triazol-4-yl)-7-((2-(trimethylsilyl)ethoxy)methyl)-7*H*-pyrrolo[2,3-*d*]pyrimidin-4-amine (8a)**

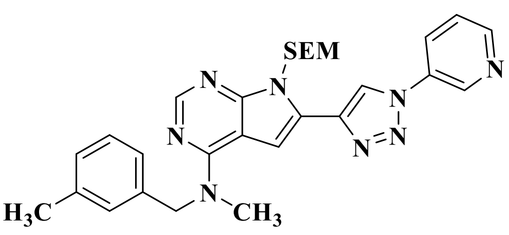

The compound was prepared as described in General Procedure A starting with **3a** (150 mg, 0.369 mmol) and 3-azidopyridine (58 mg, 0.48 mmol). Purification by silica-gel flash column chromatography (EtOAc/*n*-pentane, 4:6) gave 145 mg (0.275 mmol, 75%) of a brown viscous liquid. ^{1}H NMR (600 MHz, DMSO-d_6) δ: 9.18 (d, J = 2.8 Hz, 2H), 8.73 (dd, J = 4.7, 1.4 Hz, 1H), 8.38-8.36 (m, 1H), 8.27 (s, 1H), 7.71-7.68 (m, 1H), 7.21 (t, J = 7.6 Hz, 1H), 7.15 (s, 1H), 7.09 (s, 1H), 7.07-7.05 (m, 2H), 5.97 (s, 2H), 5.03 (s, 2H), 3.48 (t, J = 7.9 Hz, 2H), 3.36 (s, 3H), 2.26 (s, 3H), 0.77 (t, J = 8.0 Hz, 2H), -0.19 (s, 9H); ^{13}C NMR (151 MHz, DMSO-d_6) δ: 156.6, 153.0, 151.7, 149.9, 141.3, 140.2, 137.9, 137.6, 133.0, 128.4, 128.0, 127.6, 127.5, 125.3, 124.6, 124.0, 121.1, 102.7, 101.9, 70.2, 65.2, 52.7, 37.2, 21.0, 17.0, -1.5 (3C).

N-Methyl-6-(1-(pyridin-3-yl)-1H-1,2,3-triazol-4-yl)-N-((tetrahydro-2H-pyran-4-yl) methyl)-7-((2-(trimethylsilyl)ethoxy)methyl)-7H-pyrrolo[2,3-d]pyrimidin-4-amine (8b)

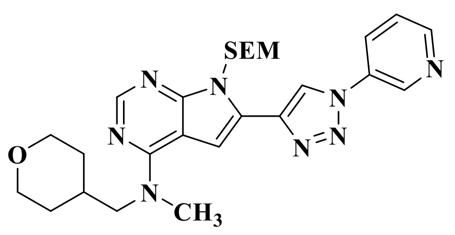

The compound was prepared as described in General Procedure A, starting with **3b** (150 mg, 0.374 mmol) and 3-azidopyridine (58 mg, 0.48 mmol). Purification by silica-gel flash column chromatography (EtOAc/n-pentane, 2:3) gave 120 mg, (0.230 mmol, 62%) of a brown viscous liquid. ^{1}H NMR (600 MHz, DMSO-d_6) δ: 9.21 (s, 1H), 9.20 (s, 1H), 8.74 (dd, J = 4.8, 1.4 Hz, 1H), 8.40-8.38 (m, 1H), 8.23 (s, 1H), 7.72-7.70 (m, 1H), 7.18 (s, 1H), 5.96 (s, 2H), 3.84 (dd, J = 11.6, 2.6 Hz, 2H), 3.71 (d, J = 7.4 Hz, 2H), 3.47 (t, J = 8.0 Hz, 2H), 3.41 (s, 3H), 3.25 (t, J = 10.7 Hz, 2H), 2.10-2.06 (s, 1H), 1.54 (d, J = 10.7 Hz, 2H), 1.33-1.27 (m, 2H), 0.76 (t, J = 7.9 Hz, 2H), -0.19 (s, 9H); ^{13}C NMR (151 MHz, DMSO-d_6) δ: 156.5, 152.9, 151.6, 149.9, 141.3, 140.3, 133.0, 128.0, 125.1, 124.6, 121.1, 102.9, 101.9, 70.2, 66.7 (2C), 65.1, 55.4, 38.9, 33.8, 30.2 (2C), 17.0, -1.5 (3C).

6-(1-Benzyl-1H-1,2,3-triazol-4-yl)-N-methyl-N-(3-methylbenzyl)-7-((2-(trimethylsilyl) ethoxy)methyl)-7H-pyrrolo[2,3-d]pyrimidin-4-amine (9a)

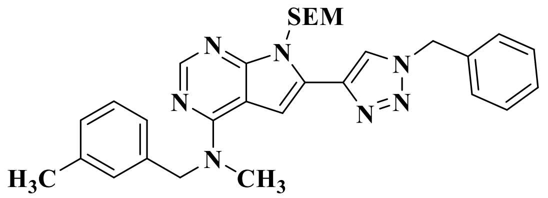

The compound was synthesized according to General Procedure **A** starting with **3a** (100 mg, 0.246 mmol) and benzyl azide (0.5 M in CH_2Cl_2, 0.64 mL, 0.32 mmol). Purification by silica-gel flash column chromatography (CH_2Cl_2/EtOAc, 3:2) gave 92 mg (0.170 mmol, 69%) of a brown viscous liquid. ^{1}H NMR (400 MHz, DMSO-d_6) δ: 8.48 (s, 1H), 8.23 (s, 1H), 7.41-7.33 (m, 5H), 7.20 (t, J = 7.5 Hz, 1H), 7.07-7.02 (m, 4H), 5.85 (s, 2H), 5.69 (s, 2H), 5.00 (s, 2H), 3.46 (t, J = 8.0 Hz, 2H), 3.33 (s, 3H), 2.25 (s, 3H), 0.74 (t, J = 7.9 Hz, 2H), -0.19 (s, 9H); ^{13}C NMR (101 MHz, DMSO-d_6) δ: 156.5, 152.8, 151.4, 139.1, 138.0, 137.6, 135.7, 128.7 (3C), 128.4, 128.2, 127.9 (2C), 127.6, 127.5, 126.2, 124.0, 122.8, 101.8, 70.2, 65.1, 52.9, 52.6, 37.2, 21.0, 17.0, -1.5 (3C).

6-(1-Benzyl-1*H*-1,2,3-triazol-4-yl)-*N*-methyl-*N*-((tetrahydro-2*H*-pyran-4-yl)methyl)-7-((2-(trimethylsilyl)ethoxy)methyl)-7*H*-pyrrolo[2,3-*d*]pyrimidin-4-amine (9b)

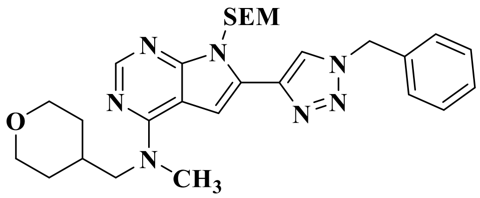

The compound was prepared as described in General Procedure A starting with **3b** (74 mg, 0.185 mmol) and benzyl azide (0.5 M in CH_2Cl_2, 0.48 mL, 0.24 mmol). The reaction time was 3 h. Purification by silica-gel flash column chromatography (EtOAc/*n*-pentane, 3:2) gave 85 mg, (0.159 mmol, 86%) of a brown viscous liquid. ^{1}H NMR (400 MHz, DMSO-d_6) δ: 8.50 (s, 1H), 8.19 (s, 1H), 7.42.7.33 (m, 5H), 7.08 (s, 1H), 5.84 (s, 2H), 5.70 (s, 2H), 3.84-3.80 (m, 2H), 3.68 (d, *J* = 7.3 Hz, 2H), 3.44 (t, *J* = 7.8 Hz, 2H), 3.38 (s, 3H), 3.26-3.20 (m, 2H), 2.08-2.03 (s, 1H), 1.52 (d, *J* = 12.4 Hz, 2H), 1.30-1.23 (m, 2H), 0.73 (t, *J* = 8.0 Hz, 2H), -0.20 (s, 9H); ^{13}C NMR (101 MHz, DMSO-d_6) δ: 156.4, 152.7, 151.3, 139.2, 135.8, 128.7 (2C), 128.2, 127.9 (2C), 125.9, 122.8, 102.0, 101.9, 70.2, 66.7 (2C), 65.1, 55.4, 52.9, 38.9, 33.8, 30.2 (2C), 21.0, 17.0, -1.5 (3C).

6-(1-(4-Fluorobenzyl)-1*H*-1,2,3-triazol-4-yl)-*N*-methyl-*N*-(3-methylbenzyl)-7-((2-(trimethylsilyl)ethoxy)methyl)-7*H*-pyrrolo[2,3-*d*]pyrimidin-4-amine (10a)

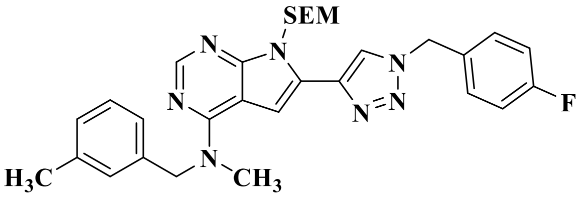

The compound was prepared as described in General Procedure A starting with **3a** (150 mg, 0.369 mmol) and 1-(azidomethyl)-4-fluorobenzene (72 mg, 0.48 mmol). Purification by silica-gel flash column chromatography (EtOAc/*n*-pentane, 3:7) gave 144 mg, (0.258 mmol, 70%) of a brown solid. ^{1}H NMR (600 MHz, DMSO-d_6) δ: 8.46 (s, 1H), 8.23 (s, 1H), 7.45-7.42 (m, 2H), 7.24-7.19 (m, 3H), 7.07-7.03 (m, 4H), 5.84 (s, 2H), 5.68 (s, 2H), 5.00 (s, 2H), 3.45 (t, *J* = 7.9 Hz, 2H), 3.33 (s, 3H), 2.25 (s, 3H), 0.74 (t, *J* = 8.1 Hz, 2H), -0.20 (s, 9H); ^{13}C NMR (151 MHz, DMSO-d_6) δ: 161.9 (d, J = 244.4 Hz), 156.5, 152.8, 151.4, 139.1, 138.0, 137.6, 132.0 (d, J = 3.3 Hz), 130.3 (d, J = 8.3 Hz, 2C), 128.4, 127.6, 127.5, 126.2, 124.0, 122.7, 115.6 (d, J = 21.6 Hz, 2C), 101.8 (2C), 70.2, 65.1, 52.7, 52.1, 37.2, 21.0, 17.0, -1.5 (3C).

6-(1-(4-Fluorobenzyl)-1*H*-1,2,3-triazol-4-yl)-*N*-methyl-*N*-((tetrahydro-2*H*-pyran-4-yl)methyl)-7-((2-(trimethylsilyl)ethoxy)methyl)-7*H*-pyrrolo[2,3-*d*]pyrimidin-4-amine (10b)

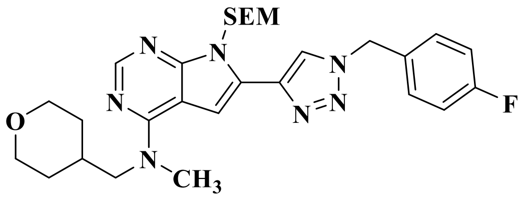

The compound was synthesized according to General Procedure A starting with **3b** (150 mg, 0.374 mmol) and 1-(azidomethyl)-4-fluorobenzene (74 mg, 0.48 mmol). Purification by silica-gel flash column chromatography (EtOAc/*n*-pentane, 7:3) gave 140 mg, (0.254 mmol, 68%) of a brown viscous liquid. ^{1}H NMR (600 MHz, DMSO-d_6) δ: 8.49 (s, 1H), 8.19 (s, 1H), 7.46-7.44 (m, 2H), 7.23 (t, J = 8.9 Hz, 2H), 7.07 (s, 1H), 5.83 (s, 2H), 5.69 (s, 2H), 3.83-3.81 (m, 2H), 3.68 (d, J = 7.4 Hz, 2H), 3.44 (t, J = 8.0 Hz, 2H), 3.38 (s, 3H), 3.25-3.21 (m, 2H), 2.07-2.03 (m, 1H), 1.53-1.50 (m, 2H), 1.31-1.24 (m, 2H), 0.72 (t, J = 8.0 Hz, 2H), -0.21 (s, 9H); ^{13}C NMR (151 MHz, DMSO-d_6) δ: 161.9 (d, J = 244.4 Hz), 156.4, 152.7, 151.3, 139.2, 132.0 (d, J = 3.0 Hz), 130.38 (d, J = 8.4 Hz, 2C), 125.9, 122.7, 115.6 (d, J = 21.6 Hz, 2C), 102.0, 101.9, 70.1, 66.7 (2C), 65.1, 55.4, 52.1, 38.8, 33.8, 30.2 (2C), 17.0, -1.5 (3C).

***N*-Methyl-*N*-(3-methylbenzyl)-6-(1-(4-methylbenzyl)-1*H*-1,2,3-triazol-4-yl)-7-((2-(trimethylsilyl)ethoxy)methyl)-7*H*-pyrrolo[2,3-*d*]pyrimidin-4-amine (11a)**

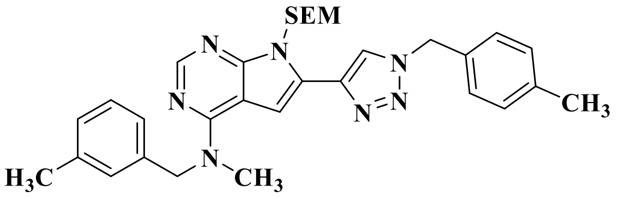

The compound was synthesized according to General Procedure A with **3a** (150 mg, 0.369 mmol) and 1-(azidomethyl)-4-methylbenzene (71 mg, 0.48 mmol). Purification by silica-gel flash column chromatography (EtOAc/*n*-pentane, 3:7) gave 150 mg, (0.271 mmol, 73%) of a brown solid. ^{1}H NMR (400 MHz, DMSO-d_6) δ: 8.43 (s, 1H), 8.23 (s, 1H), 7.26 (d, J = 8.0 Hz, 2H), 7.22-7.17 (m, 3H), 7.07-7.03 (m, 4H), 5.84 (s, 2H), 5.62 (s, 2H), 5.00 (s, 2H), 3.45 (t, J = 7.8 Hz, 2H), 3.33 (s, 3H), 2.28 (s, 3H), 2.25 (s, 3H), 0.74 (t, J = 8.0 Hz, 2H), -0.19 (s, 9H); ^{13}C NMR (101 MHz, DMSO-d_6) δ: 156.5, 152.8, 151.4, 139.1, 138.0, 137.6, 137.5, 132.7, 129.3 (2C), 128.45, 128.0 (2C), 127.6, 127.5, 126.2, 124.0, 122.6, 101.89, 101.85, 70.2, 65.1, 52.7, 52.6, 37.2, 21.0, 20.6, 17.0, -1.5 (3C).

_N_-Methyl-6-(1-(4-methylbenzyl)-1_H_-1,2,3-triazol-4-yl)-_N_-((tetrahydro-2_H_-pyran-4-yl)methyl)-7-((2-(trimethylsilyl)ethoxy)methyl)-7_H_-pyrrolo[2,3-_d_]pyrimidin-4-amine (11b)

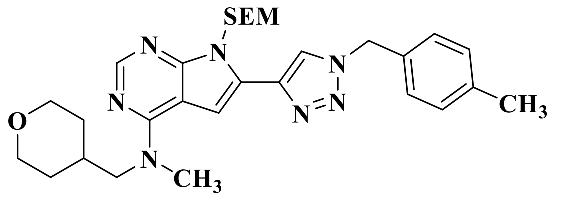

The compound was synthesized according to General Procedure A using **3b** (150 mg, 0.374 mmol) and 1-(azidomethyl)-4-methylbenzene (72 mg, 0.49 mmol). Purification by silica-gel flash column chromatography (EtOAc/_n_-pentane, 1:1) gave 131 mg, (0.239 mmol, 64%) of a brown viscous liquid. ^{1}H NMR (400 MHz, DMSO-d_6) δ: 8.46 (s, 1H), 8.18 (s, 1H), 7.27 (d, J = 8.1 Hz, 2H), 7.19 (d, J = 7.9 Hz, 2H), 7.07 (s, 1H), 5.83 (s, 2H), 5.63 (s, 2H), 3.82 (dd, J = 11.9, 3.3 Hz, 2H), 3.68 (d, J = 7.3 Hz, 2H), 3.44 (t, J = 7.8 Hz, 2H), 3.38 (s, 3H), 3.23 (t, J = 10.7 Hz, 2H), 2.29 (s, 3H), 2.08-2.02 (m, 1H), 1.52 (d, J = 10.4 Hz, 2H), 1.33-1.23 (m, 2H), 0.73 (t, J = 8.0 Hz, 2H), -0.19 (s, 9H); ^{13}C NMR (101 MHz, DMSO-d_6) δ: 156.4, 152.7, 151.3, 139.2, 137.5, 132.8, 129.3 (2C), 128.0 (2C), 126.0, 122.6, 101.98, 101.94, 70.2, 66.7 (2C), 65.1, 55.4, 52.7, 33.8, 30.2 (2C), 20.6, 17.0, -1.55 (3C).

6-(1-(4-Methoxybenzyl)-1_H_-1,2,3-triazol-4-yl)-_N_-methyl-_N_-(3-methylbenzyl)-7-((2-(trimethylsilyl)ethoxy)methyl)-7_H_-pyrrolo[2,3-_d_]pyrimidin-4-amine (12a)

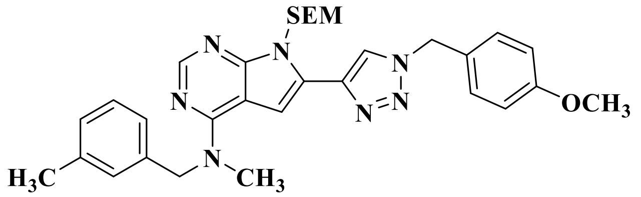

The compound was prepared as described in General Procedure A, starting with **3a** (150 mg, 0.369 mmol) and 1-(azidomethyl)-4-methoxybenzene (78 mg, 0.48 mmol). Purification by silica-gel flash column chromatography (EtOAc/_n_-pentane, 1:1) gave 164 mg, (0.288 mmol, 78%) of a brown viscous liquid. ^{1}H NMR (600 MHz, DMSO-d_6) δ: 8.42 (s, 1H), 8.23 (s, 1H), 7.33 (d, J = 8.7 Hz, 2H), 7.20 (t, J = 7.5 Hz, 1H), 7.07-7.03 (m, 3H), 6.95-6.93 (m, 3H), 5.84 (s, 2H), 5.59 (s, 2H), 5.00 (s, 2H), 3.73 (s, 3H), 3.45 (t, J = 7.8 Hz, 2H), 3.33 (s, 3H), 2.25 (s, 3H), 0.74 (t, J = 8.1 Hz, 2H), -0.19 (s, 9H); ^{13}C NMR (151 MHz, DMSO-d_6) δ: 159.2, 156.5, 152.8, 151.4, 139.1, 138.0, 137.6, 129.6 (3C), 128.4, 127.6, 127.5, 126.3, 124.0, 122.5, 114.1 (3C), 101.9, 101.8, 70.2, 65.1, 55.1, 52.5, 37.2, 21.0, 17.0, -1.5 (3C).

6-(1-(4-Methoxybenzyl)-1*H*-1,2,3-triazol-4-yl)-*N*-methyl-*N*-((tetrahydro-2*H*-pyran-4-yl)methyl)-7-((2-(trimethylsilyl)ethoxy)methyl)-7*H*-pyrrolo[2,3-*d*]pyrimidin-4-amine (12b)

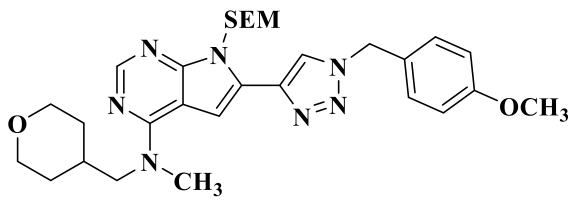

The compound was synthesized according to General Procedure A using **3b** (150 mg, 0.374 mmol) and 1-(azidomethyl)-4-methoxybenzene (80 mg, 0.49 mmol). Purification by silica-gel flash column chromatography (EtOAc/*n*-pentane, 3:2) gave 173 mg, (0.307 mmol, 82%) of a brown viscous liquid. ^{1}H NMR (600 MHz, DMSO-d_6) δ: 8.44 (s, 1H), 8.19 (s, 1H), 7.34 (d, J = 8.7 Hz, 2H), 7.06 (s, 1H), 6.94 (d, J = 8.7 Hz, 2H), 5.83 (s, 2H), 5.60 (s, 2H), 3.82 (dd, J = 11.5, 2.6 Hz, 2H), 3.74 (s, 3H), 3.67 (d, J = 7.4 Hz, 2H), 3.44 (t, J = 7.9 Hz, 2H), 3.37 (s, 3H), 3.25-3.21 (m, 2H), 2.07-2.03 (m, 1H), 1.51 (d, J = 10.7 Hz, 2H), 1.31-1.24 (s, 2H), 0.73 (t, J = 7.9 Hz, 2H), -0.20 (s, 9H); ^{13}C NMR (151 MHz, DMSO-d_6) δ: 159.2, 156.4, 152.7, 151.3, 139.2, 129.6 (2C), 127.6, 126.0, 122.4, 114.1 (2C), 101.9, 70.1, 66.7 (2C), 65.1, 55.4, 55.1 (2C), 52.5, 38.8, 33.8, 30.2 (2C), 17.0, -1.5 (3C).

***N*-Methyl-*N*-(3-methylbenzyl)-6-(1-(pyridin-3-ylmethyl)-1*H*-1,2,3-triazol-4-yl)-7-((2-(trimethylsilyl)ethoxy)methyl)-7*H*-pyrrolo[2,3-*d*]pyrimidin-4-amine (13a)**

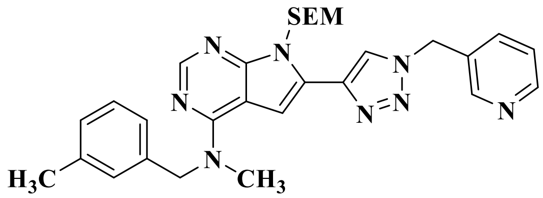

The compound was synthesized according to General Procedure A using **3a** (150 mg, 0.369 mmol) and 3-(azidomethyl)pyridine (64 mg, 0.48 mmol). Purification by silica-gel flash column chromatography (CH_2Cl_2/acetone, 4:1) gave 137 mg, (0.253 mmol, 69%) of a brown viscous liquid. ^{1}H NMR (400 MHz, DMSO-d_6) δ: 8.65 (s, 1H), 8.56 (dd, J = 4.8, 1.6 Hz, 1H), 8.53 (s, 1H), 8.23 (s, 1H), 7.79-7.76 (m, 1H), 7.44-7.40 (m, 1H), 7.20 (t, J = 7.5 Hz, 1H), 7.07-7.03 (m, 4H), 5.86 (s, 2H), 5.75 (s, 2H), 5.00 (s, 2H), 3.45 (t, J = 7.9 Hz, 2H), 3.33 (s, 3H), 2.25 (s, 3H), 0.74 (t, J = 7.9 Hz, 2H), -0.20 (s, 9H); ^{13}C NMR (101 MHz, DMSO-d_6) δ: 156.5, 152.8, 151.4, 149.5, 149.2, 139.2, 138.0, 137.6, 135.9, 131.4, 128.4, 127.6, 127.5, 126.1, 124.0, 123.8, 122.9, 101.9, 101.8, 70.2, 65.1, 52.6, 50.5, 37.2, 21.0, 17.0, -1.5 (3C).

N-Methyl-6-(1-(pyridin-3-ylmethyl)-1H-1,2,3-triazol-4-yl)-N-((tetrahydro-2H-pyran-4-yl)methyl)-7-((2-(trimethylsilyl)ethoxy)methyl)-7H-pyrrolo[2,3-d]pyrimidin-4-amine (13b)

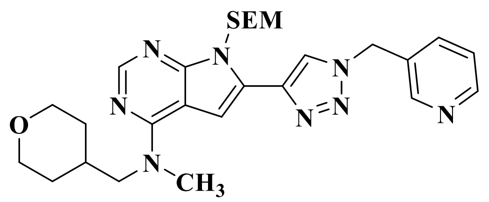

The compound was synthesized according to General Procedure A using **3b** (150 mg, 0.374 mmol) and 3-(azidomethyl)pyridine (65 mg, 0.49 mmol). Purification by silica-gel flash column chromatography (CH_2Cl_2/MeOH, 19:1) gave 137 mg (0.256 mmol, 68%) of a brown viscous liquid. ^{1}H NMR (600 MHz, DMSO-d_6) δ: 8.68 (s, 1H), 8.59 (s, 1H), 8.56 (s, 1H), 8.19 (s, 1H), 7.79 (d, J = 7.9 Hz, 1H), 7.44 (dd, J = 8.0, 4.6 Hz, 1H), 7.08 (s, 1H), 5.85 (s, 2H), 5.77 (s, 2H), 3.82 (dd, J = 11.5, 2.6 Hz, 2H), 3.68 (d, J = 7.4 Hz, 2H), 3.44 (t, J = 7.9 Hz, 2H), 3.38 (s, 3H), 3.26-3.21 (m, 2H), 2.07-2.03 (m, 1H), 1.52 (d, J = 10.7 Hz, 2H), 1.31-1.25 (m, 2H), 0.73 (t, J = 8.0 Hz, 2H), -0.21 (s, 9H); ^{13}C NMR (151 MHz, DMSO-d_6) δ: 156.4, 152.7, 151.3, 149.5, 149.3, 139.3, 135.9, 125.8, 123.9, 122.9, 102.1, 101.9, 70.2, 66.7 (2C), 65.1, 55.4, 54.9, 50.5, 38.8, 33.8, 30.2 (2C), 17.0, -1.5 (3C).

3-(4-(4-(Methyl(3-methylbenzyl)amino)-7-((2-(trimethylsilyl)ethoxy)methyl)-7H-pyrrolo[2,3-d]pyrimidin-6-yl)-1H-1,2,3-triazol-1-yl)propan-1-ol (14a)

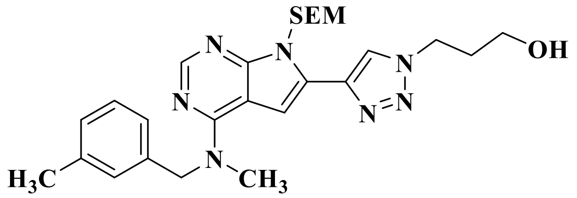

The compound was synthesized according to General Procedure A using **3a** (150 mg, 0.369 mmol) and 3-azidopropan-1-ol (48 mg, 0.48 mmol). Purification by silica-gel flash column chromatography (CH_2Cl_2,(MeOH, 31:9) gave 120 mg, (0.236 mmol, 64%) of a brown viscous liquid. ^{1}H NMR (600 MHz, DMSO-d_6) δ: 8.43 (s, 1H), 8.24 (s, 1H), 7.21 (t, J = 7.5 Hz, 1H), 7.08-7.02 (m, 4H), 5.87 (s, 2H), 5.01 (s, 2H), 4.69 (t, J = 5.1 Hz, 1H), 4.48 (t, J = 7.1 Hz, 2H), 3.49-3.43 (m, 4H), 3.34 (s, 3H), 2.26 (s, 3H), 2.03-1.98 (m, 2H), 0.78 (t, J = 8.0 Hz, 2H), -0.16 (s, 9H); ^{13}C NMR (151 MHz, DMSO-d_6) δ: 156.5, 152.8, 151.4, 138.7, 138.0, 137.6, 128.4, 127.6, 127.5, 126.4, 124.0, 122.8, 101.9, 101.7, 70.2, 65.1, 57.4, 54.9, 46.8, 37.2, 32.9, 21.0, 17.0, -1.5 (3C).

3-(4-(4-(Methyl((tetrahydro-2*H*-pyran-4-yl)methyl)amino)-7-((2-(trimethylsilyl) ethoxy)methyl)-7*H*-pyrrolo[2,3-*d*]pyrimidin-6-yl)-1*H*-1,2,3-triazol-1-yl)propan-1-ol (14b)

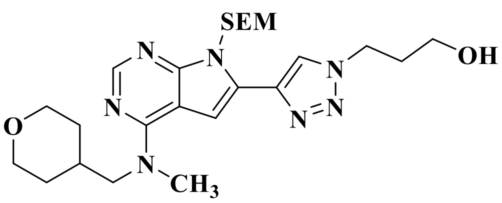

The compound was synthesized according to General Procedure A using **3b** (150 mg, 0.374 mmol) and 3-azidopropan-1-ol (49 mg, 0.48 mmol). Purification by silica-gel flash column chromatography (MeOH/CH_2Cl_2, 1:9) gave 102 mg (0.203 mmol, 54%) of a brown viscous liquid. ^{1}H NMR (600 MHz, DMSO-d_6) δ: 8.45 (s, 1H), 8.19 (s, 1H), 7.06 (s, 1H), 5.86 (s, 2H), 4.70 (t, J = 5.0 Hz, 1H), 4.50 (t, J = 7.1 Hz, 2H), 3.84-3.82 (m, 2H), 3.69 (d, J = 7.4 Hz, 2H), 3.47-3.45 (m, 4H), 3.39 (s, 3H), 3.24 (t, J = 10.7 Hz, 2H), 2.10-2.06 (m, 1H), 2.04-2.00 (m, 2H), 1.53 (d, J = 10.5 Hz, 2H), 1.32-1.25 (m, 2H), 0.76 (t, J = 8.1 Hz, 2H), -0.17 (s, 9H); ^{13}C NMR (151 MHz, DMSO-d_6) δ: 156.4, 152.7, 151.3, 138.8, 126.2, 122.8, 101.9, 101.8, 70.1, 66.7 (2C), 65.1, 57.4, 55.4, 46.8, 38.8, 33.8, 32.9, 30.2 (2C), 17.0, -1.5 (3C).

***N*-Methyl-*N*-(3-methylbenzyl)-6-(1-(tetrahydro-2*H*-pyran-4-yl)-1*H*-1,2,3-triazol-4-yl)-7-((2-(trimethylsilyl)ethoxy)methyl)-7*H*-pyrrolo[2,3-*d*]pyrimidin-4-amine (15a)**

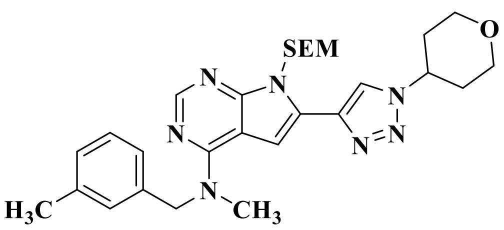

The compound was prepared as described in General Procedure A starting with **3a** (150 mg, 0.369 mmol) and 4-azidotetrahydro-2H-pyran (61 mg, 0.48 mmol). Purification by silica-gel flash column chromatography (EtOAc/*n*-pentane, 1:4) gave 148 mg, (0.27 mmol, 75%) of a brown viscous liquid. ^{1}H NMR (600 MHz, DMSO-d_6) δ: 8.54 (s, 1H), 8.24 (s, 1H), 7.21 (t, J = 7.6 Hz, 1H), 7.08-7.02 (m, 4H), 5.90 (s, 2H), 5.01 (s, 2H), 4.87-4.81 (s, 1H), 4.01-3.98 (m, 2H), 3.52 (td, J = 11.7, 2.2 Hz, 2H), 3.46 (t, J = 7.9 Hz, 2H), 3.34 (s, 3H), 2.26 (s, 3H), 2.11-2.08 (m, 2H), 2.06-1.99 (m, 2H), 0.76 (t, J = 7.9 Hz, 2H), -0.17 (s, 9H); ^{13}C NMR (151 MHz, DMSO-d_6) δ: 156.5, 152.8, 151.4, 138.8, 138.0, 137.6, 128.4, 127.6, 127.5, 126.4, 124.0, 121.0, 101.9, 101.8, 70.2, 65.6 (2C), 65.2, 56.3, 52.6, 37.2, 32.8 (2C), 21.0, 17.0, -1.5 (3C).

N-Methyl-6-(1-(tetrahydro-2H-pyran-4-yl)-1H-1,2,3-triazol-4-yl)-N-((tetrahydro-2H-pyran-4-yl)methyl)-7-((2-(trimethylsilyl)ethoxy)methyl)-7H-pyrrolo[2,3-d]pyrimidin-4-amine (15b)

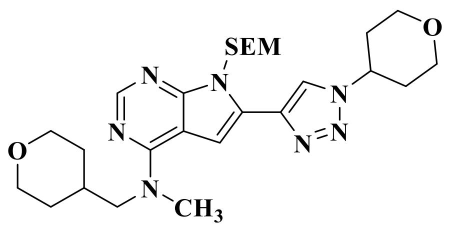

The compound was prepared as described in General Procedure A, starting with **3b** (150 mg, 0.374 mmol) and 4-azidotetrahydro-2H-pyran (62 mg, 0.48 mmol). Purification by silica-gel flash column chromatography (CH_2Cl_2/MeOH, 4:1) gave 170 mg (0.322 mmol, 86%) of a brown viscous liquid. ^{1}H NMR (600 MHz, DMSO-d_6) δ: 8.56 (s, 1H), 8.19 (s, 1H), 7.06 (s, 1H), 5.89 (s, 2H), 4.88-4.83 (m, 1H), 4.02-3.99 (m, 2H), 3.84-3.81 (m, 2H), 3.69 (d, J = 7.4 Hz, 2H), 3.56-3.51 (m, 2H), 3.45 (t, J = 7.8 Hz, 2H), 3.39 (s, 3H), 3.26-3.22 (m, 2H), 2.12-2.10 (m, 2H), 2.08-2.01 (m, 3H), 1.53 (d, J = 12.5 Hz, 2H), 1.32-1.25 (m, 2H), 0.75 (t, J = 7.9 Hz, 2H), -0.18 (s, 9H); ^{13}C NMR (151 MHz, DMSO-d_6) δ: 156.4, 152.7, 151.3, 138.9, 126.1, 121.0, 101.9, 70.2, 66.7 (2C), 65.6 (3C), 65.1, 56.3, 55.4, 38.8, 33.8, 32.8, 31.2, 30.2 (2C), 17.0, -1.5 (3C).

N-Methyl-N-(3-methylbenzyl)-6-(1-(1-methylpiperidin-4-yl)-1H-1,2,3-triazol-4-yl)-7-((2-(trimethylsilyl)ethoxy)methyl)-7H-pyrrolo[2,3-d]pyrimidin-4-amine (16a)

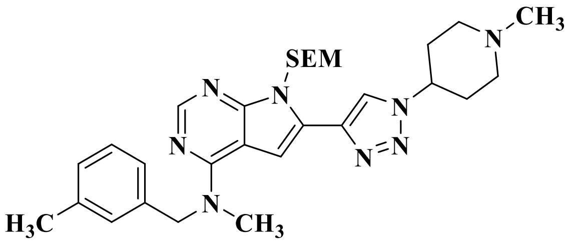

The compound was synthesized according to General Procedure A using **3a** (150 mg, 0.374 mmol) and 4-azido-1-methylpiperidine (67 mg, 0.48 mmol). The crude material was further used for -SEM deprotection without any purification.

N-Methyl-6-(1-(1-methylpiperidin-4-yl)-1H-1,2,3-triazol-4-yl)-N-((tetrahydro-2H-pyran-4-yl)methyl)-7-((2-(trimethylsilyl)ethoxy)methyl)-7H-pyrrolo[2,3-d]pyrimidin-4-amine (16b)

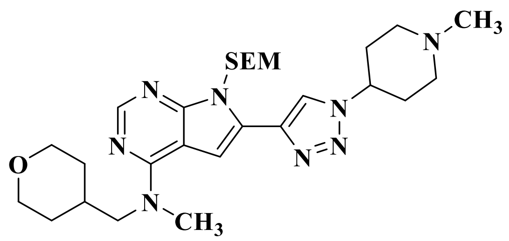

The compound was synthesized according to General Procedure A using starting material **3b** (150 mg, 0.368 mmol) and 4-azido-1-methylpiperidine (67 mg, 0.48 mmol). The crude product was further used for SEM deprotection without any purification.

N-Methyl-N-(3-methylbenzyl)-6-(1-((tetrahydro-2H-pyran-4-yl)methyl)-1H-1,2,3-triazol-4-yl)-7-((2-(trimethylsilyl)ethoxy)methyl)-7H-pyrrolo[2,3-d]pyrimidin-4-amine (17a)

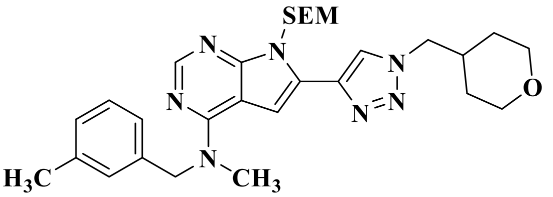

The compound was synthesized according to General Procedure A **3a** (150 mg, 0.374 mmol) and 4-(azidomethyl)tetrahydro-2H-pyran (68 mg, 0.48 mmol). Purification by silica-gel flash column chromatography (EtOAc/n-pentane, 4:1) gave 114 mg (0.208 mmol, 56%) of a brown viscous liquid. ^{1}H NMR (600 MHz, DMSO-d_6) δ: 8.42 (s, 1H), 8.24 (s, 1H), 7.20 (t, J = 7.5 Hz, 1H), 7.08-7.03 (m, 4H), 5.86 (s, 2H), 5.01 (s, 2H), 4.35 (d, J = 7.1 Hz, 2H), 3.84 (dd, J = 11.6, 2.6 Hz, 2H), 3.48 (t, J = 8.1 Hz, 2H), 3.34 (s, 3H), 3.26 (td, J = 11.7, 2.1 Hz, 2H), 2.26 (s, 3H), 2.12-2.09 (m, 1H), 1.45 (dd, J = 12.8, 2.1 Hz, 2H), 1.32-1.25 (m, 2H), 0.77 (t, J = 7.9 Hz, 2H), -0.16 (s, 9H); ^{13}C NMR (151 MHz, DMSO-d_6) δ: 156.5, 152.8, 151.3, 138.7, 138.0, 137.6, 128.4, 127.6, 127.5, 126.4, 124.0, 123.1, 101.9, 101.7, 70.2, 66.4 (2C), 65.2, 54.7, 52.7, 37.2, 35.6, 29.7 (2C), 21.0, 17.1, -1.5 (3C).

***N*-Methyl-*N*-((tetrahydro-2*H*-pyran-4-yl)methyl)-6-(1-((tetrahydro-2*H*-pyran-4-yl)methyl)-1*H*-1,2,3-triazol-4-yl)-7-((2-(trimethylsilyl)ethoxy)methyl)-7*H*-pyrrolo[2,3-*d*]pyrimidin-4-amine (17b)**

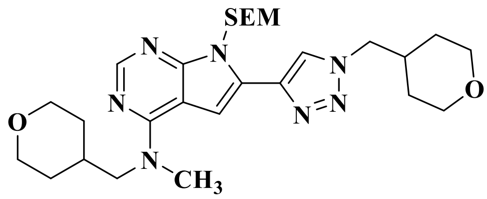

The compound was synthesized according to General Procedure A using **3b** (150 mg, 0.374 mmol) and 4-(azidomethyl)tetrahydro-2*H*-pyran (69 mg, 0.49 mmol). Purification by silica-gel flash column chromatography (MeOH/CH_2Cl_2, 1:9) gave 164 mg, (0.303 mmol, 81%) of a brown viscous liquid. ^{1}H NMR (600 MHz, DMSO-d_6) δ: 8.44 (s, 1H), 8.19 (s, 1H), 7.06 (s, 1H), 5.85 (s, 2H), 4.36 (d, J = 7.1 Hz, 2H), 3.86-3.82 (m, 4H), 3.69 (d, J = 7.4 Hz, 2H), 3.46 (t, J = 7.8 Hz, 2H), 3.39 (s, 3H), 3.29-3.22 (m, 4H), 2.14-2.10 (m, 1H), 2.08-2.04 (m, 1H), 1.53 (d, J = 12.7 Hz, 2H), 1.46 (d, J = 10.7 Hz, 2H), 1.31-1.27 (m, 4H), 0.76 (t, J = 7.9 Hz, 2H), -0.17 (s, 9H); ^{13}C NMR (151 MHz, DMSO-d_6) δ: 156.4, 152.7, 151.3, 138.8, 126.1, 123.1, 101.9, 101.8, 70.2, 66.7 (2C), 66.4 (2C), 65.1, 55.4, 54.7, 38.8, 35.6, 33.8, 30.2 (2C), 29.7 (2C), 17.1, -1.5 (3C).

2. **Profiling of 27a towards a kinase panel**

Table S1. Profiling of compound **27a** towards a panel of 49 kinases at 1000 nM test concentration. The ATP concentration was equal to K_M.

**Kinase**	**Inhibition (%)**	**Inhibition (%)**	**Average inhibition (%)**	**Difference in data points (%)**
ABL1	99	99	99	0
ABL2 (Arg)	100	100	100	0
AKT1 (PKB alpha)	3	1	2	2
ALK	24	25	24	0
AURKA (Aurora A)	35	40	38	5
AURKB (Aurora B)	47	46	47	2
AXL	16	16	16	1
BLK	69	69	69	0
BTK	50	42	46	8
CAMK1D (CaMKI delta)	2	9	5	6
CDK1/cyclin B	24	29	27	5
CHEK1 (CHK1)	0	9	5	9
CLK1	8	8	8	0
CSF1R (FMS)	99	99	99	0
DAPK1	13	-5	4	18
DYRK1A	5	4	5	1
EGFR (ErbB1)	84	87	85	3
EPHA2	88	91	89	3
ERBB2 (HER2)	33	32	32	1
ERBB4 (HER4)	13	15	14	2
FER	16	14	15	2
FGFR1	9	8	9	1
FLT1 (VEGFR1)	32	30	31	1
FLT3	42	45	44	3
FLT4 (VEGFR3)	15	16	16	1
FRAP1 (mTOR)	-4	-2	-3	2
FYN	75	77	76	2
GSK3B (GSK3 beta)	59	62	61	2
HCK	77	76	77	1
ITK	58	51	55	7
JAK1	22	-3	9	24
JAK2	25	26	26	1
KIT	56	58	57	2
LTK (TYK1)	25	25	25	1
LYN A	66	66	66	1
LYN B	74	79	76	5
MET (cMet)	10	16	13	6
MINK1	75	77	76	2
MKNK1 (MNK1)	3	1	2	2
MUSK	73	72	72	1
NEK1	28	28	28	0
PAK1	4	0	2	4

Kinase	Inhibition (%)	Inhibition (%)	Average inhibition (%)	Difference in data points (%)
PDGFRB (PDGFR beta)	32	35	34	2
PLK1	5	6	6	1
RET	28	30	29	2
ROCK1	2	-2	0	3
SRC	96	95	96	0
TEK (Tie2)	44	40	42	3
YES1	102	99	101	3

3. IC_{50} curves

Table S2. Representative IC_{50} curves for CSF1R using Z-LYTE assay platform.

Comp.	Data points	IC_{50} curve
**21b**	20	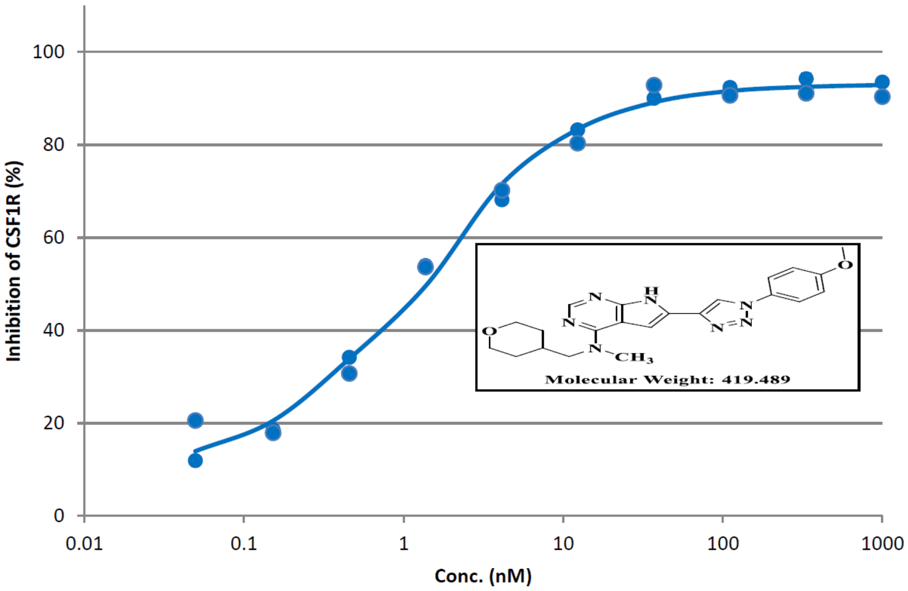 IC_{50} (nM)= 1.2, R^2= 0.997, p-value:0.0001, Hill coefficient: 0.913.
**25b**	20	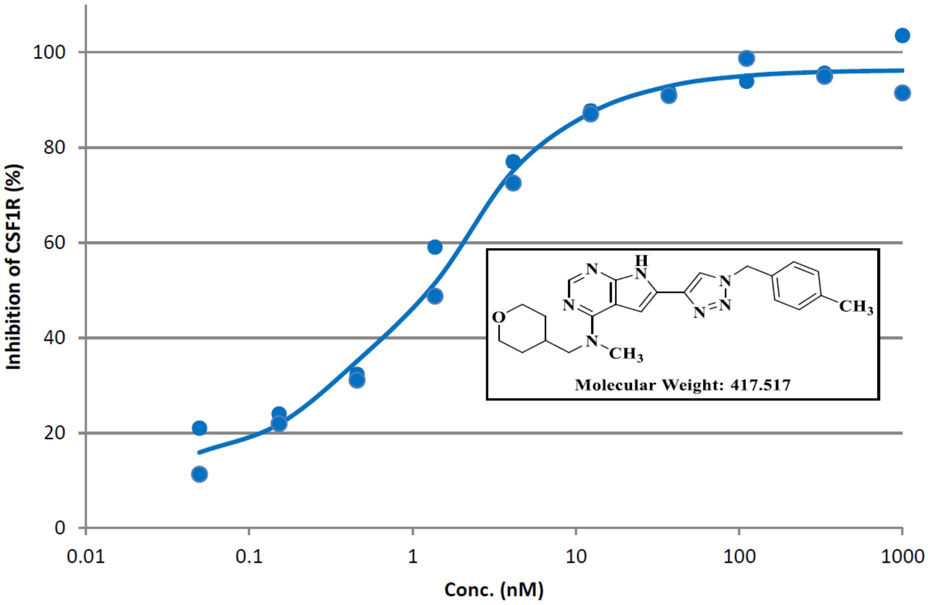 IC_{50} (nM)= 1.3, R^2= 998, p-value: 0.0001, Hill coefficient: 0.981.

Comp.	Data points	IC50 curve
**27a**	20	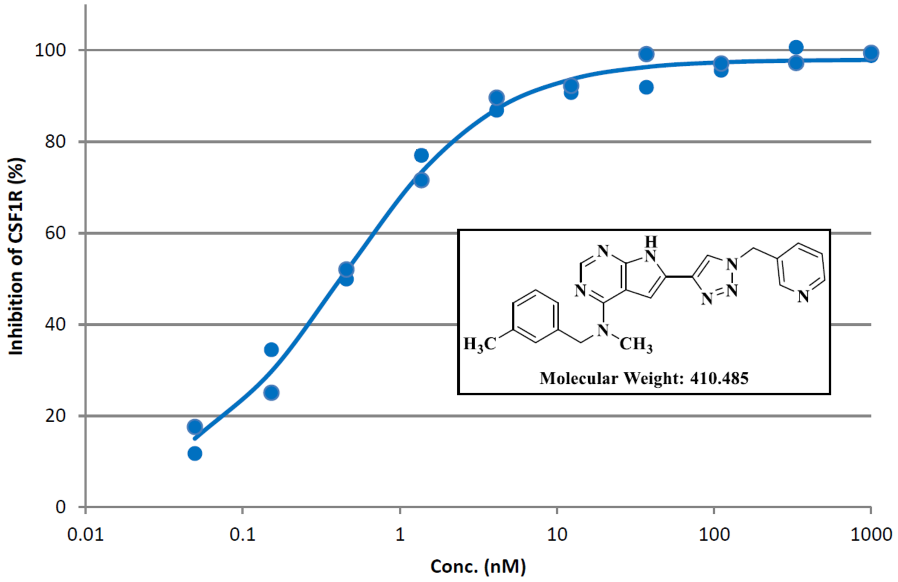 IC50 (nM)= 0.4, R^2= 0.998, p-value:0.0001, Hill coefficient: 0.903.
PLX3397	80	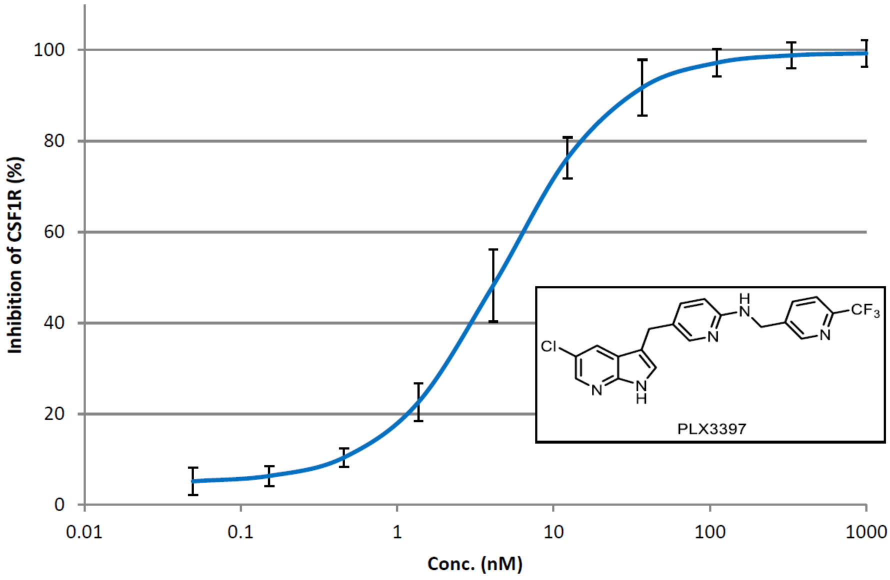 IC50 (nM)= 4.7±2.0, R^2= 0.998, p-value:0.0001, Hill coefficient: 1.18.

4. Spectral data for the inhibitor candidates 18a-31b

Compound 18a

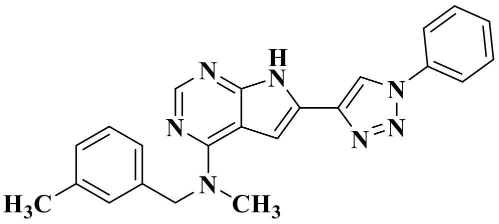

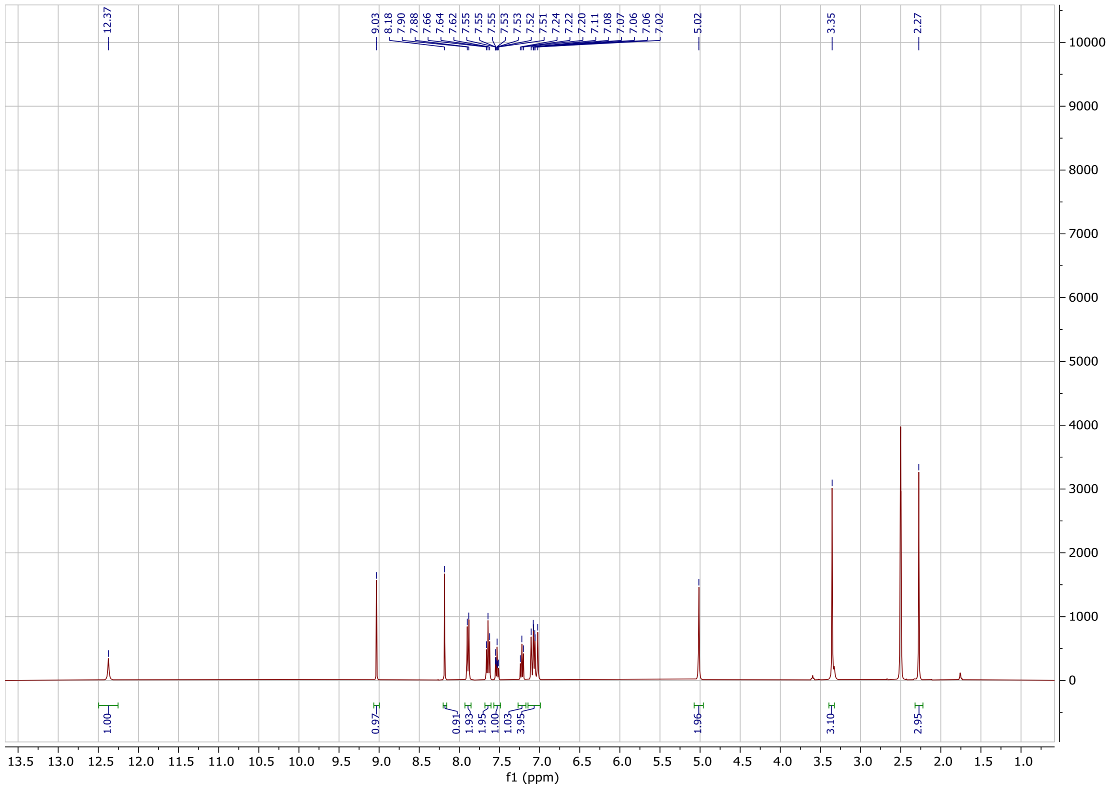

Figure S1. ^{1}H NMR (400 MHz, DMSO-d_6) spectrum of compound **18a**.

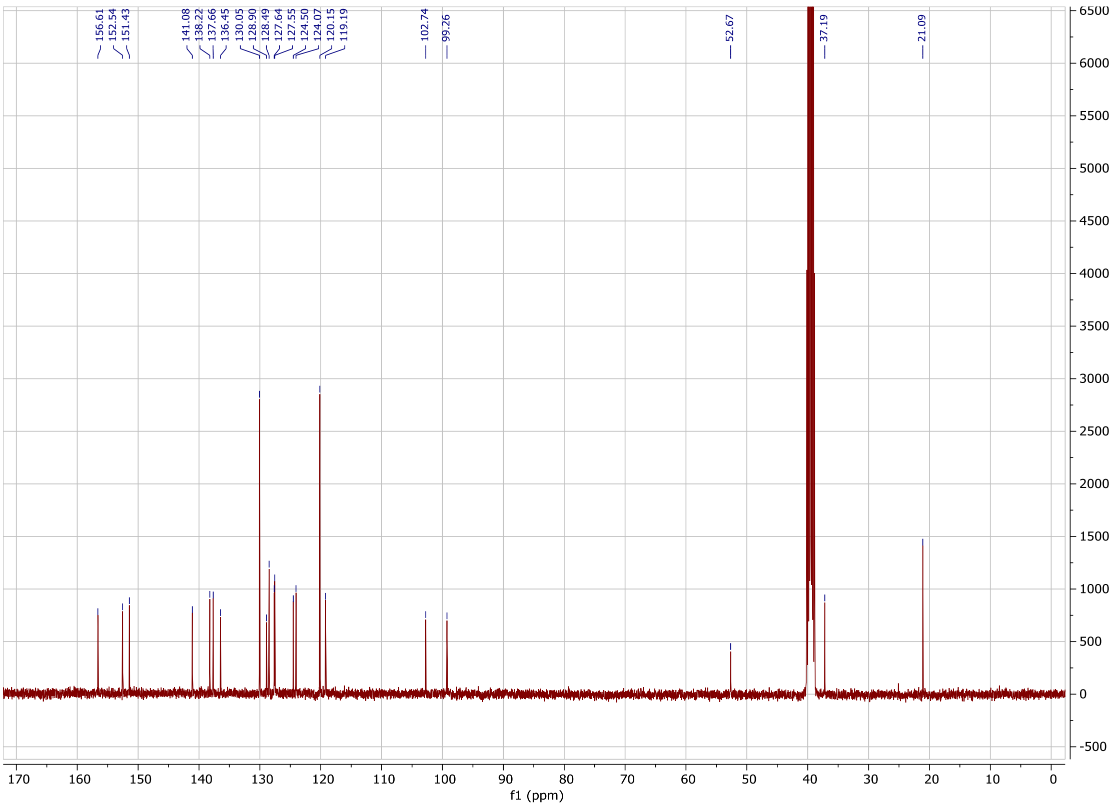

Figure S2. ^{13}C NMR (101 MHz, DMSO-d_6) spectrum of compound **18a**.

Elemental Composition Report **Page 1**

Single Mass Analysis
Tolerance = 2.0 PPM / DBE: min = -1.5, max = 50.0
Element prediction: Off
Number of isotope peaks used for i-FIT = 3

Monoisotopic Mass, Even Electron Ions
746 formula(e) evaluated with 2 results within limits (all results (up to 1000) for each mass)
Elements Used:
C: 1-100 H: 1-150 N: 0-8 O: 0-12 I: 0-1

ReqID3734 59 (0.568) AM2 (Ar,35000.0,0.00,0.00); Cm (58:59)
1: TOF MS ES+

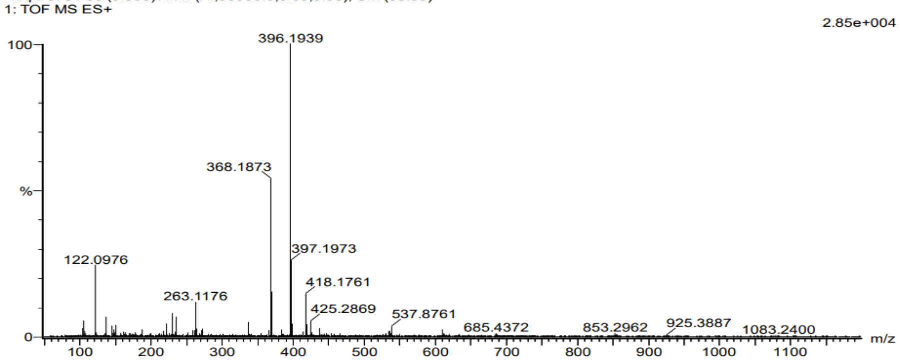

Minimum:				-1.5				
Maximum:		3000.0	2.0	50.0				
Mass	Calc. Mass	mDa	PPM	DBE	i-FIT	Norm	Conf(%)	Formula
396.1939	396.1937	0.2	0.5	16.5	339.9	0.000	99.98	C23 H22 N7
	396.1942	-0.3	-0.8	-1.5	348.4	8.441	0.02	C10 H30 N5 O11

Figure S3. HRMS (ES+, m/z) data of compound **18a**.

Compound 18b

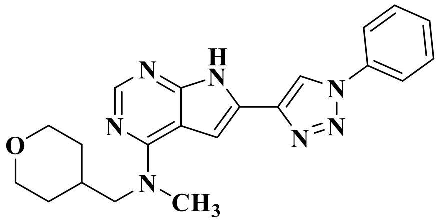

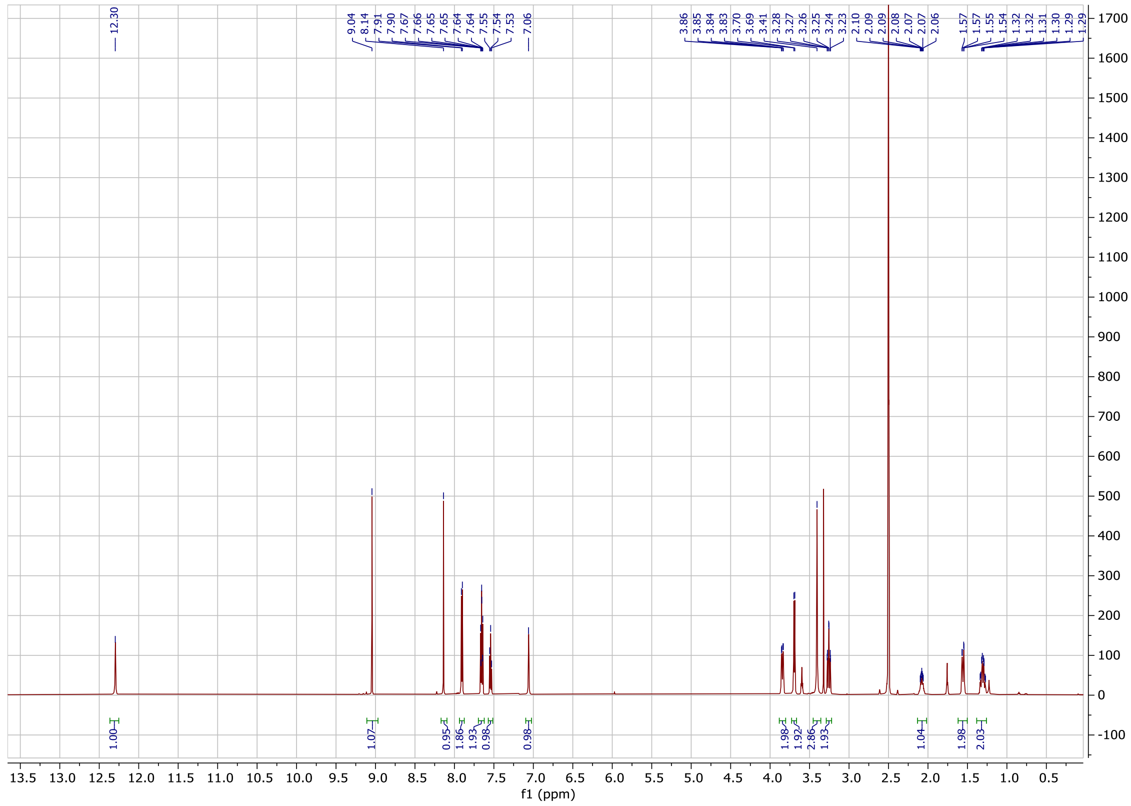

Figure S4. ^{1}H NMR (600 MHz, DMSO-d_6) spectrum of compound **18b.**

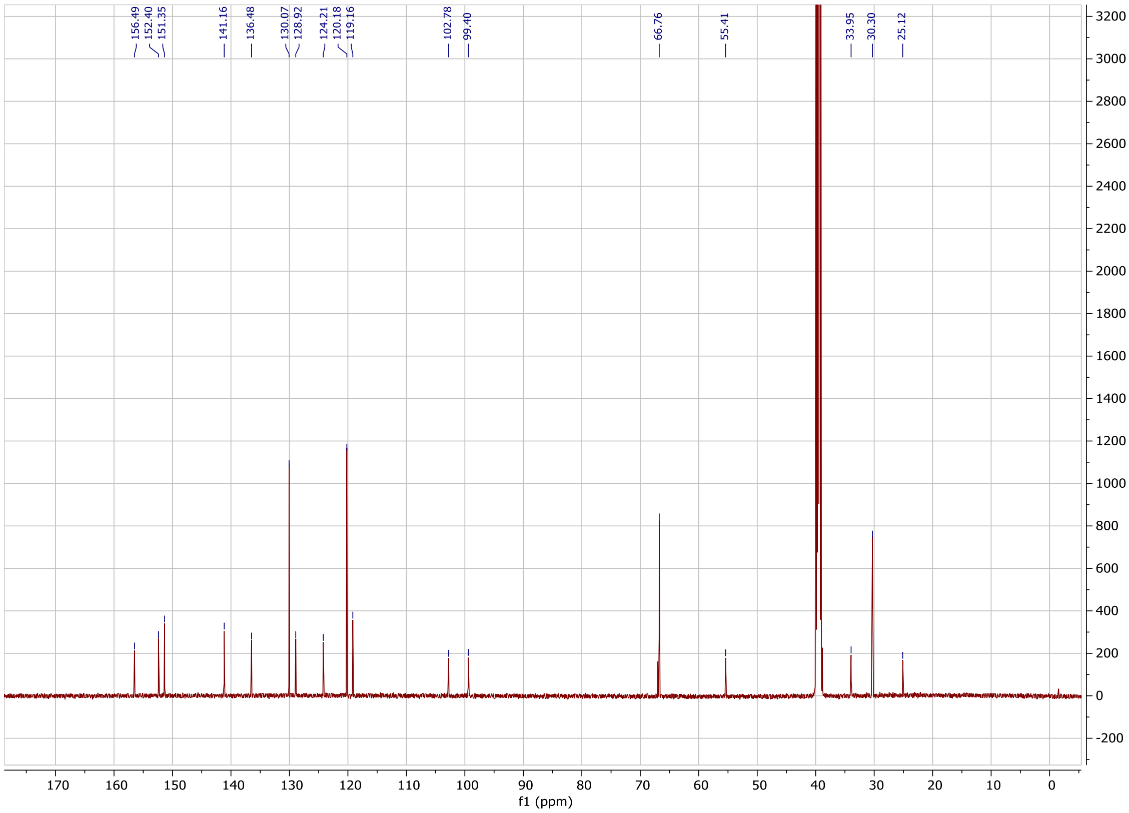

Figure S5. ^{13}C NMR (151 MHz, DMSO-d_6) spectrum of compound **18b**.

Elemental Composition Report **Page 1**

Single Mass Analysis
Tolerance = 2.0 PPM / DBE: min = -1.5, max = 50.0
Element prediction: Off
Number of isotope peaks used for i-FIT = 3

Monoisotopic Mass, Even Electron Ions
1361 formula(e) evaluated with 2 results within limits (all results (up to 1000) for each mass)
Elements Used:
C: 1-100 H: 1-150 N: 0-8 O: 0-12 Na: 0-1 I: 0-1

ReqID3697 57 (0.543) AM2 (Ar,35000.0,0.00,0.00); Cm (54:57)
1: TOF MS ES+

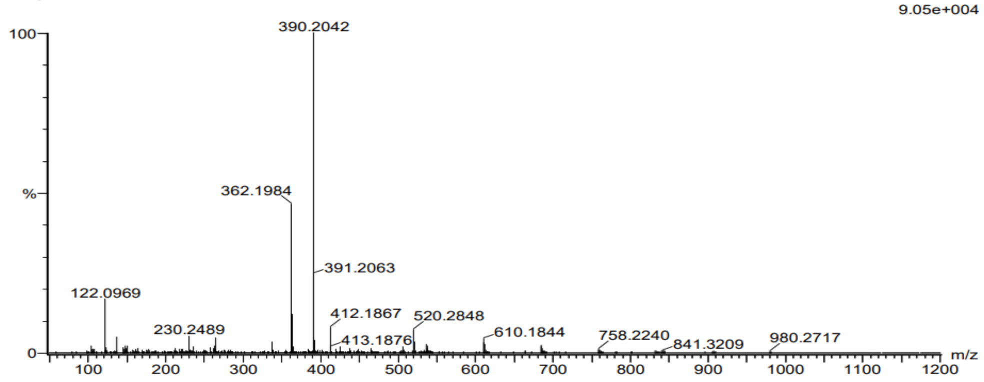

Minimum:				-1.5				
Maximum:		3000.0	2.0	50.0				
Mass	Calc. Mass	mDa	PPM	DBE	i-FIT	Norm	Conf(%)	Formula
390.2042	390.2042	0.0	0.0	13.5	507.2	0.257	77.36	C21 H24 N7 O
	390.2045	-0.3	-0.8	9.5	508.4	1.485	22.64	C23 H29 N O3 Na

Figure S6. HRMS (ES+, m/z) data of compound **18b**.

Compound 19a

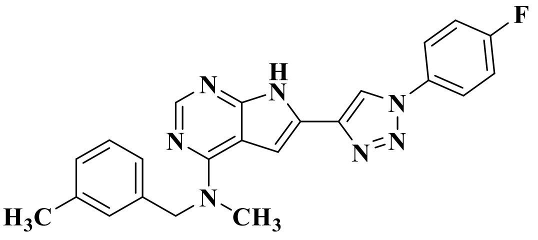

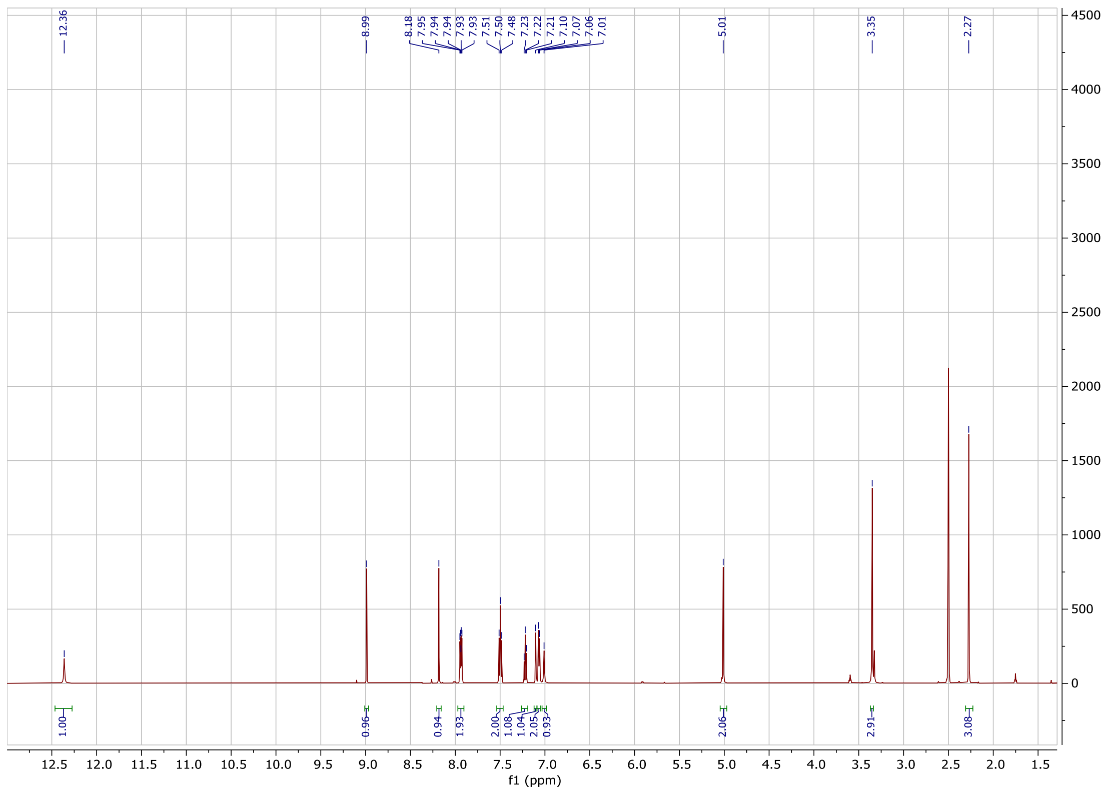

Figure S7. ^{1}H NMR (600 MHz, DMSO-d_6) spectrum of compound **19a**.

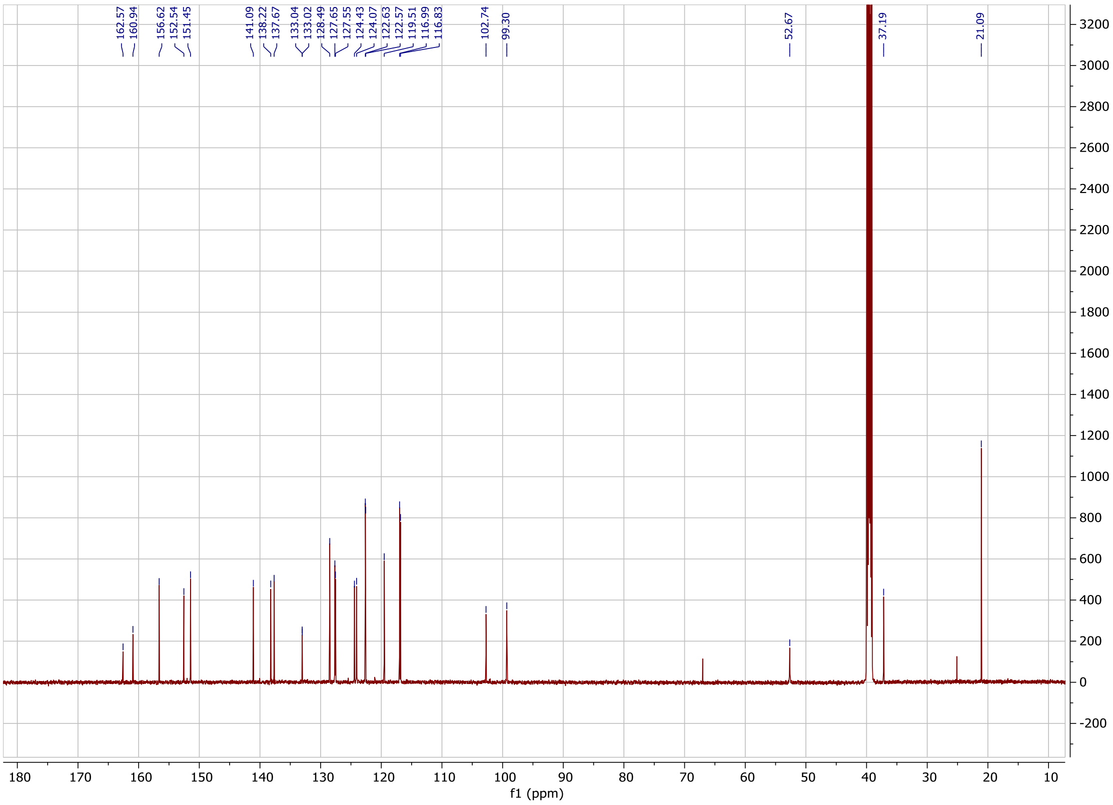

Figure S8. ^{13}C NMR (151 MHz, DMSO-d_6) spectrum of compound **19a**.

Elemental Composition Report **Page 1**

Single Mass Analysis
Tolerance = 2.0 PPM / DBE: min = -1.5, max = 50.0
Element prediction: Off
Number of isotope peaks used for i-FIT = 3

Monoisotopic Mass, Even Electron Ions
1520 formula(e) evaluated with 3 results within limits (all results (up to 1000) for each mass)
Elements Used:
C: 1-100 H: 1-150 N: 0-8 O: 0-12 F: 0-1 I: 0-1
ReqID3703 62 (0.594) AM2 (Ar,35000.0,0.00,0.00); Cm (59:62)
1: TOF MS ES+

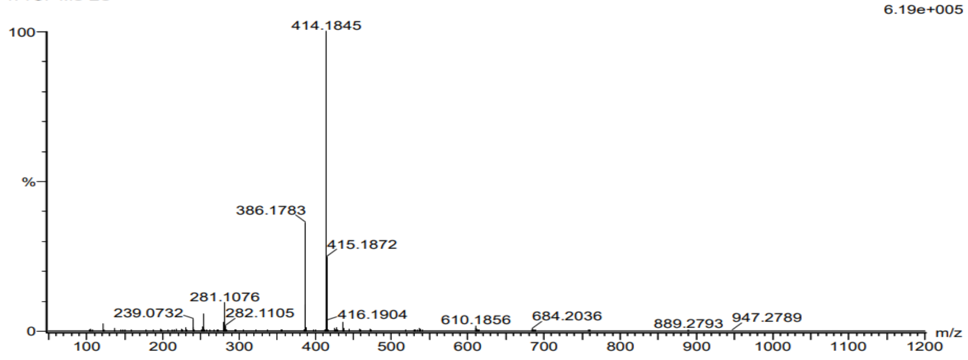

Minimum:				-1.5				
Maximum:		3000.0	2.0	50.0				
Mass	Calc. Mass	mDa	PPM	DBE	i-FIT	Norm	Conf(%)	Formula
414.1845	414.1842	0.3	0.7	16.5	769.5	0.001	99.90	C23 H21 N7 F
	414.1848	-0.3	-0.7	-1.5	778.0	8.508	0.02	C10 H29 N5 O11 F
	414.1842	0.3	0.7	0.5	776.7	7.126	0.08	C13 H33 N7 I

Figure S9. HRMS (ES+, m/z) data of compound **19a**.

Compound 19b

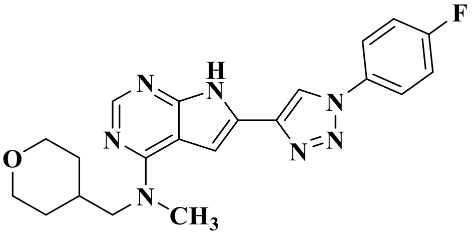

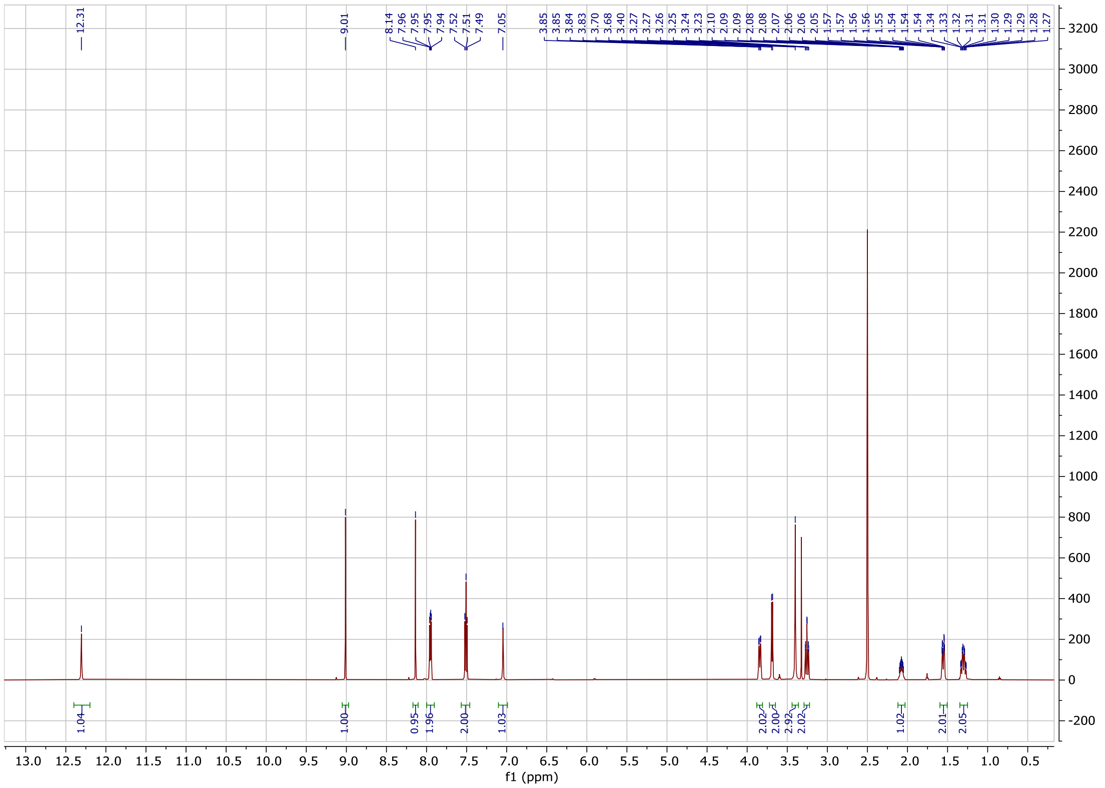

Figure S10. ^{1}H NMR (600 MHz, DMSO-d_6) spectrum of compound **19b**.

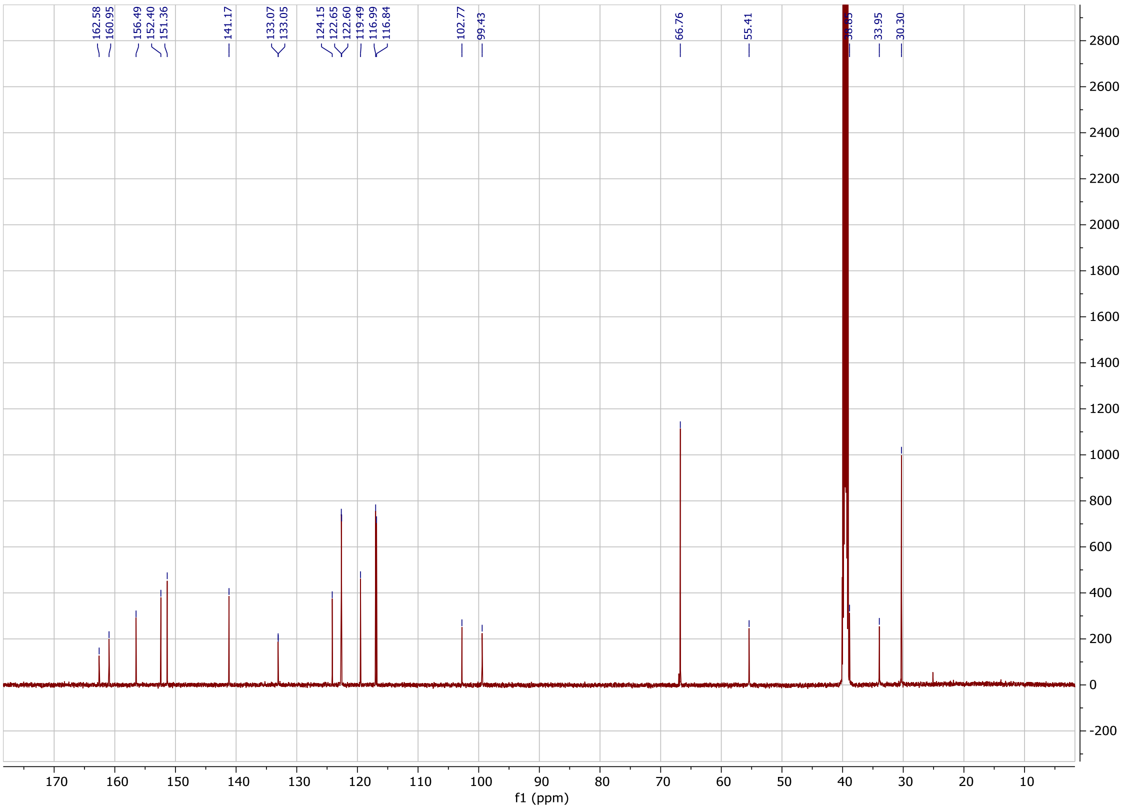

Figure S11. ^{13}C NMR (151 MHz, DMSO-d_6) spectrum of compound **19b**.

Elemental Composition Report **Page 1**

Single Mass Analysis
Tolerance = 2.0 PPM / DBE: min = -1.5, max = 50.0
Element prediction: Off
Number of isotope peaks used for i-FIT = 3

Monoisotopic Mass, Even Electron Ions
2803 formula(e) evaluated with 4 results within limits (all results (up to 1000) for each mass)
Elements Used:
C: 1-100 H: 1-150 N: 0-8 O: 0-12 F: 0-1 Na: 0-1 I: 0-1

ReqID3698 57 (0.543) AM2 (Ar,35000.0,0.00,0.00); Cm (56:57)
1: TOF MS ES+

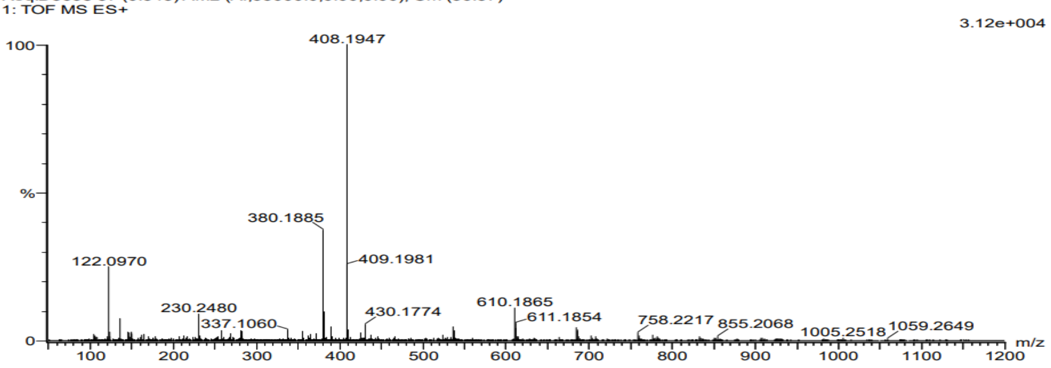

Mass	Calc. Mass	mDa	PPM	DBE	i-FIT	Norm	Conf(%)	Formula
Minimum:				-1.5				
Maximum:		3000.0	2.0	50.0				
408.1947	408.1948	-0.1	-0.2	13.5	369.4	0.119	88.80	C21 H23 N7 O F
	408.1951	-0.4	-1.0	9.5	371.5	2.191	11.18	C23 H28 N O3 F Na
	408.1942	0.5	1.2	-0.5	382.5	13.241	0.00	C11 H30 N5 O11
	408.1939	0.8	2.0	13.5	378.0	8.675	0.02	C26 H27 N O2 Na

Figure S12. HRMS (ES+, m/z) data of compound **19b**.

Compound 20a

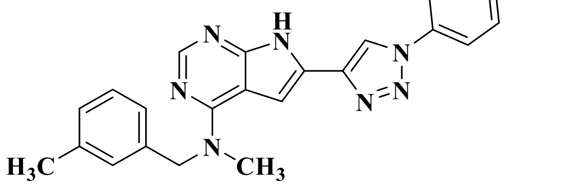

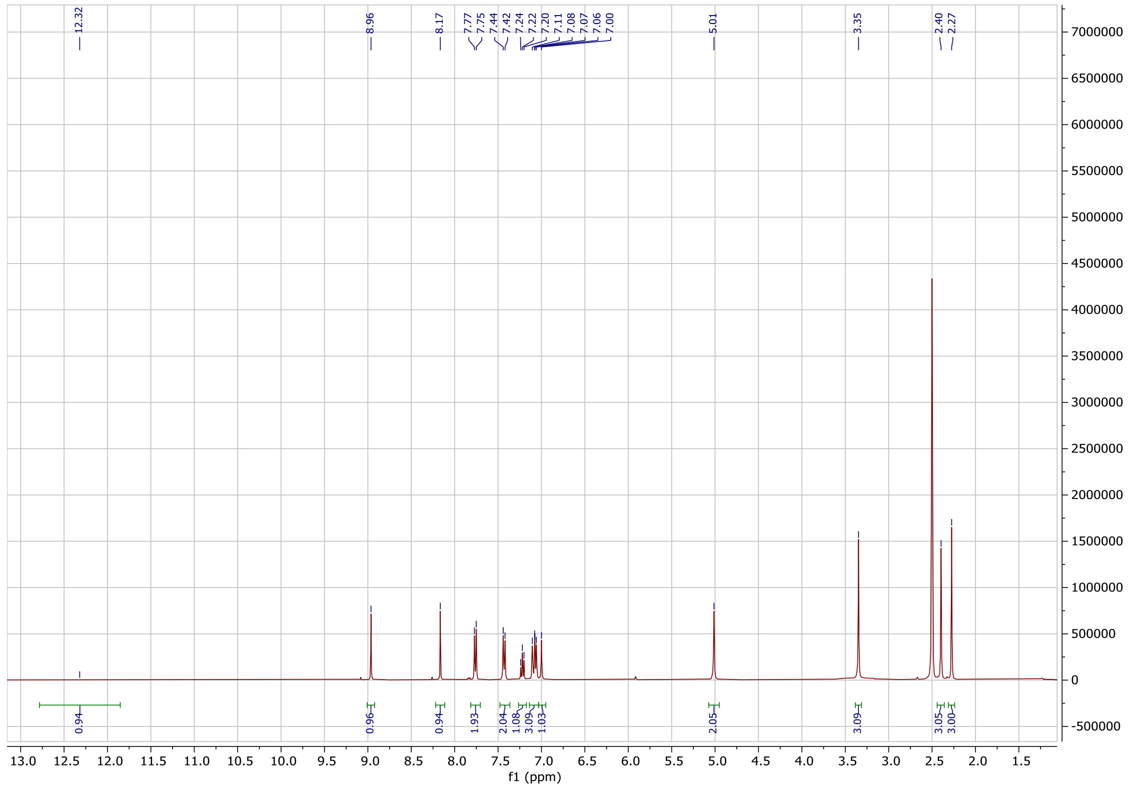

Figure S13. ^{1}H NMR (400 MHz, DMSO-d_6) spectrum of compound **20a**.

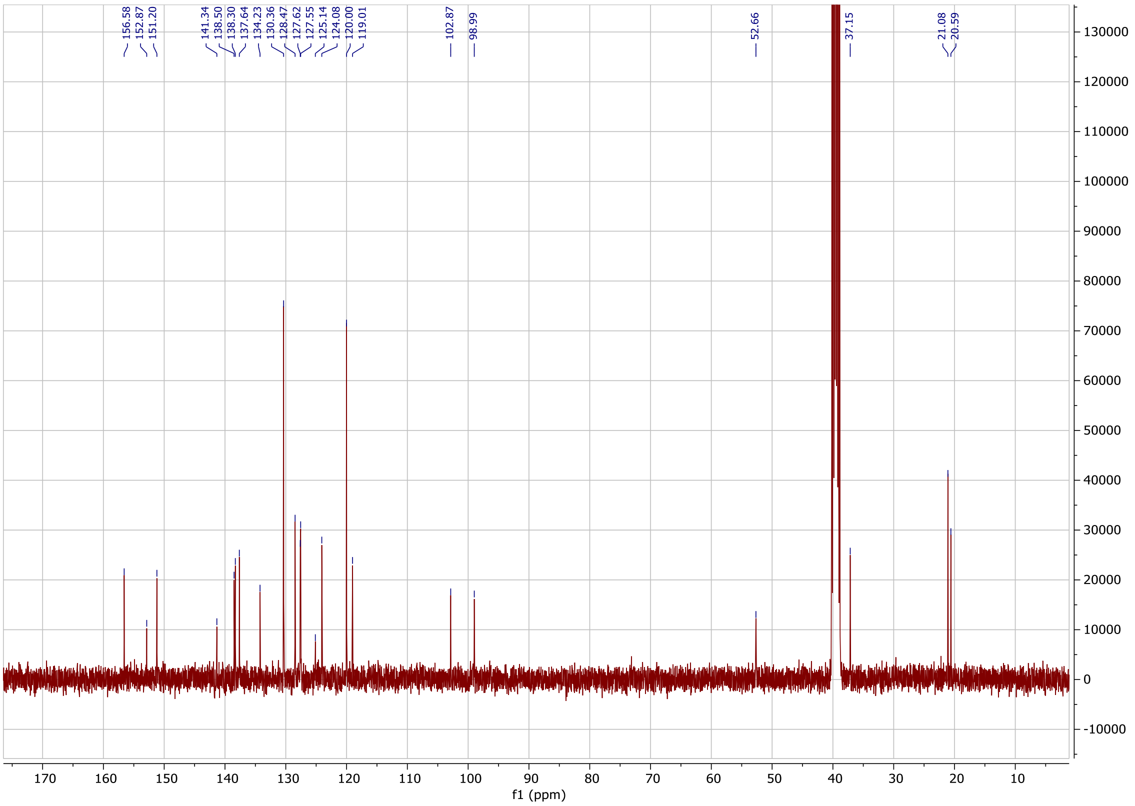

Figure S14. ^{13}C NMR (101 MHz, DMSO-d_6) spectrum of compound **20a**.

Elemental Composition Report **Page 1**

Single Mass Analysis
Tolerance = 2.0 PPM / DBE: min = -1.5, max = 50.0
Element prediction: Off
Number of isotope peaks used for i-FIT = 3

Monoisotopic Mass, Even Electron Ions
789 formula(e) evaluated with 2 results within limits (all results (up to 1000) for each mass)
Elements Used:
C: 1-100 H: 1-150 N: 0-8 O: 0-12 I: 0-1
ReqID3717 59 (0.568) AM2 (Ar,35000.0,0.00,0.00); Cm (57:59)
1: TOF MS ES+

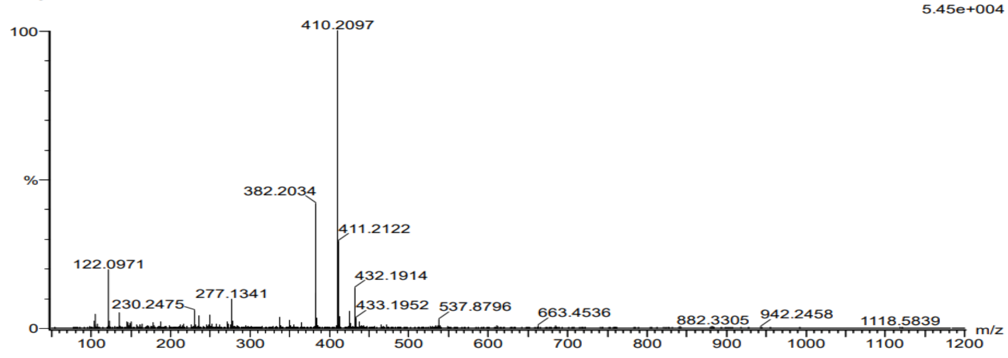

Minimum:				-1.5				
Maximum:		3000.0	2.0	50.0				
Mass	Calc. Mass	mDa	PPM	DBE	i-FIT	Norm	Conf(%)	Formula
410.2097	410.2093	0.4	1.0	16.5	435.6	0.000	100.00	C24 H24 N7
	410.2098	-0.1	-0.2	-1.5	447.6	11.981	0.00	C11 H32 N5 O11

Figure S15. HRMS (ES+, m/z) data of compound **20a**.

Compound 20b

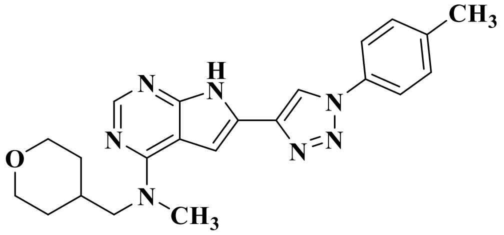

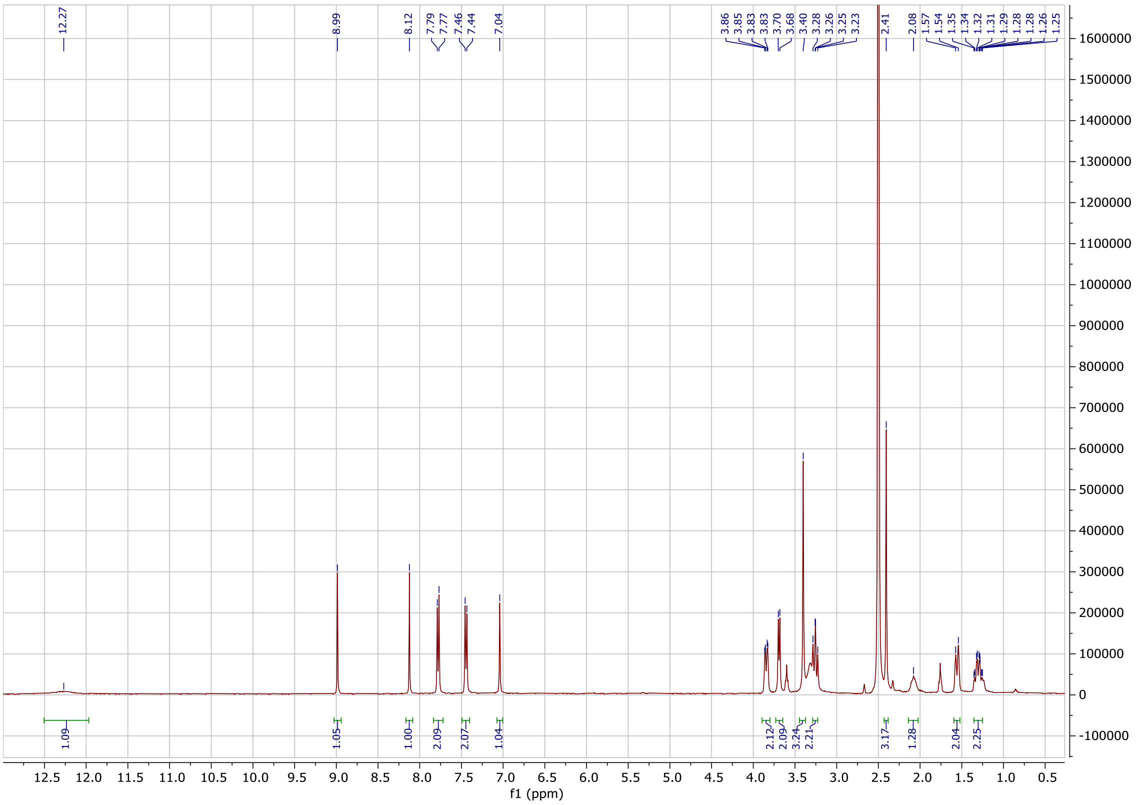

Figure S16. ^{1}H NMR (400 MHz, DMSO-d_6) spectrum of compound **20b**.

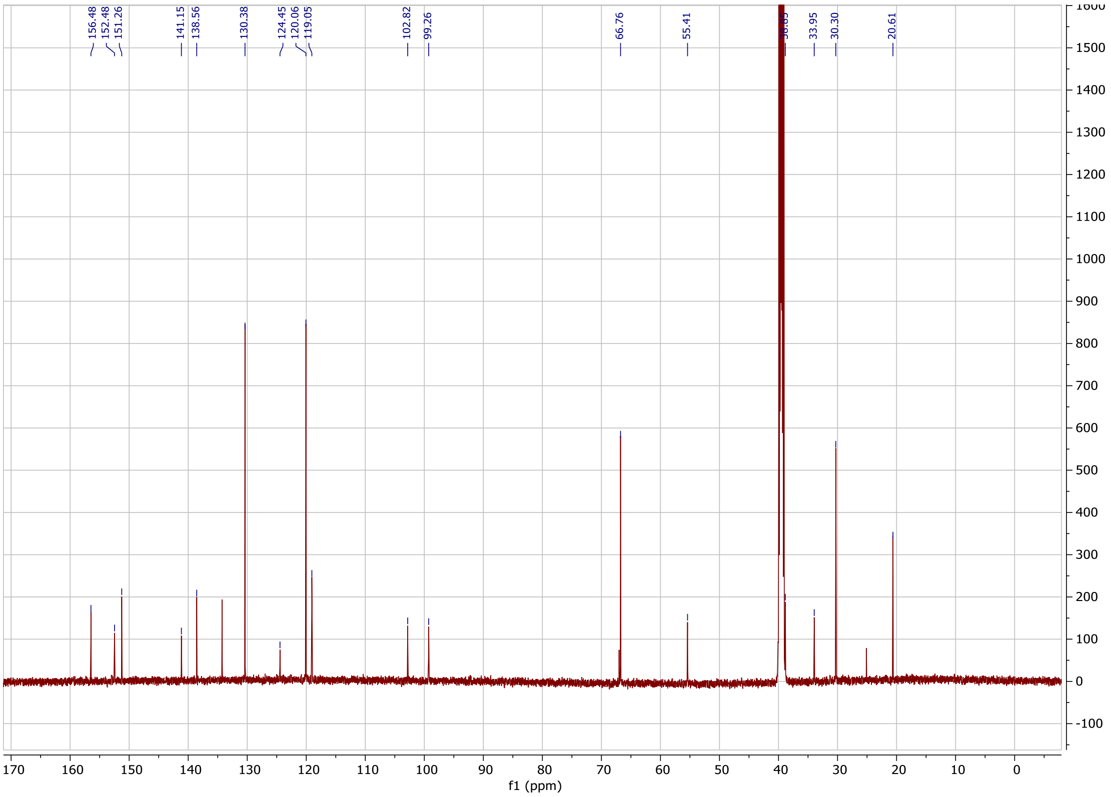

Figure S17. ^{13}C NMR (101 MHz, DMSO-d_6) spectrum of compound **20b**.

Elemental Composition Report **Page 1**

Single Mass Analysis
Tolerance = 2.0 PPM / DBE: min = -1.5, max = 50.0
Element prediction: Off
Number of isotope peaks used for i-FIT = 3

Monoisotopic Mass, Even Electron Ions
764 formula(e) evaluated with 1 results within limits (all results (up to 1000) for each mass)
Elements Used:
C: 1-100 H: 1-150 N: 0-8 O: 0-12 I: 0-1
ReqID3714 60 (0.577) AM2 (Ar,35000.0,0.00,0.00); Cm (56:60)
1: TOF MS ES+

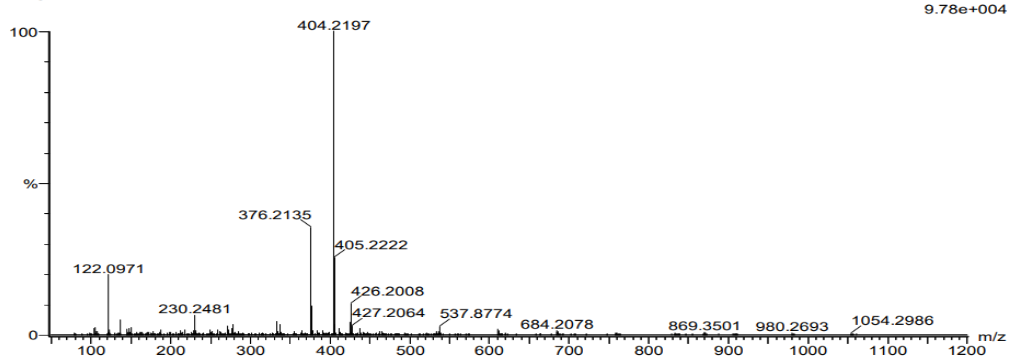

Minimum:				-1.5				
Maximum:		3000.0	2.0	50.0				
Mass	Calc. Mass	mDa	PPM	DBE	i-FIT	Norm	Conf(%)	Formula
404.2197	404.2199	-0.2	-0.5	13.5	500.9	n/a	n/a	C22 H26 N7 O

Figure S18. HRMS (ES+, m/z) data of compound **20b**.

Compound 21a

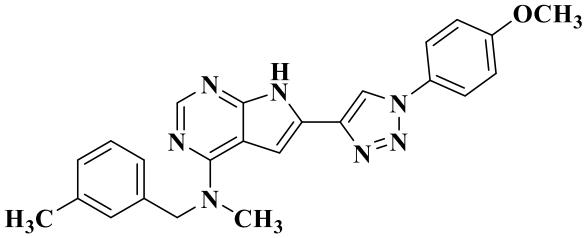

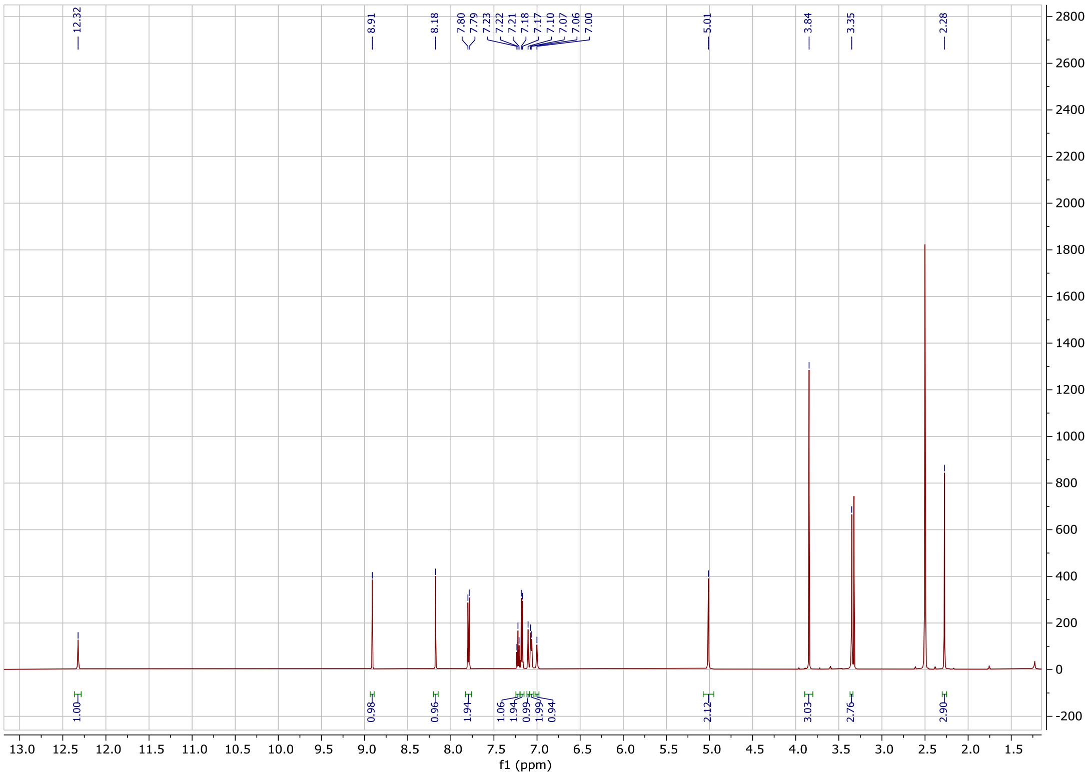

Figure S19. ^{1}H NMR (600 MHz, DMSO-d_6) spectrum of compound **21a**.

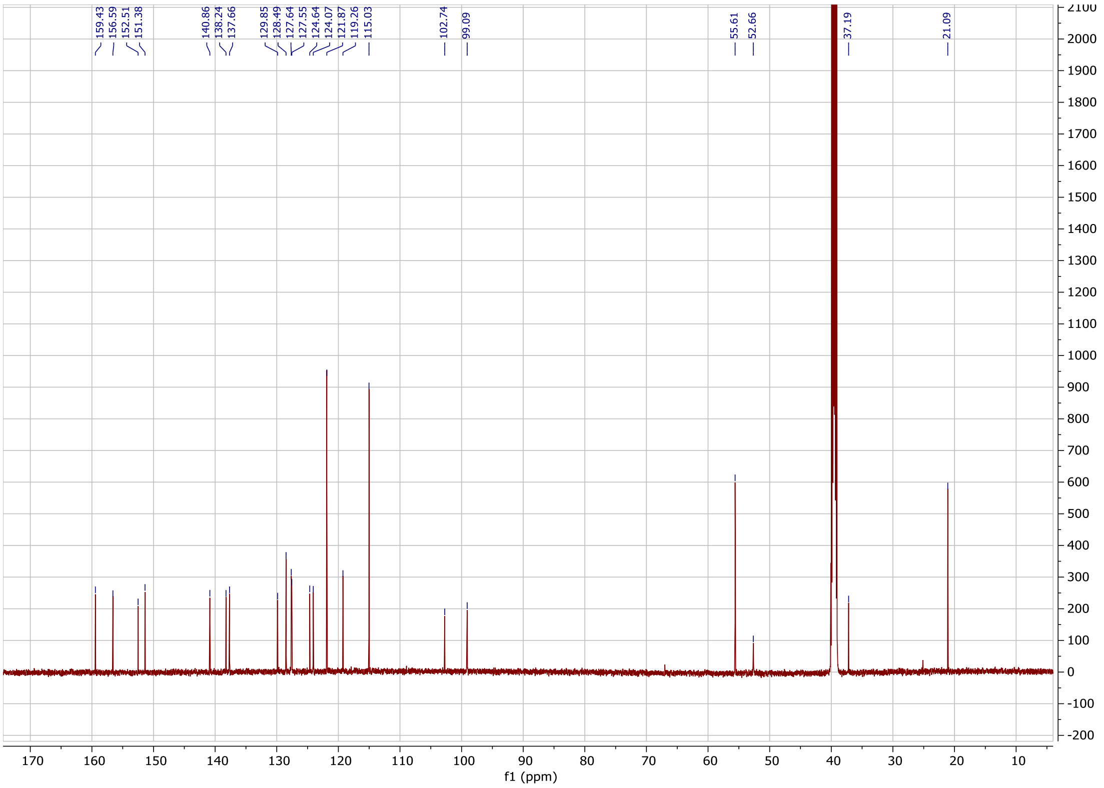

Figure S20. ^{13}C NMR (151 MHz, DMSO-d_6) spectrum of compound **21a**.

Elemental Composition Report **Page 1**

Single Mass Analysis
Tolerance = 2.0 PPM / DBE: min = -1.5, max = 50.0
Element prediction: Off
Number of isotope peaks used for i-FIT = 3

Monoisotopic Mass, Even Electron Ions
836 formula(e) evaluated with 2 results within limits (all results (up to 1000) for each mass)
Elements Used:
C: 1-100 H: 1-150 N: 0-8 O: 0-12 I: 0-1
ReqID3718 58 (0.552) AM2 (Ar,35000.0,0.00,0.00); Cm (55:58)
1: TOF MS ES+

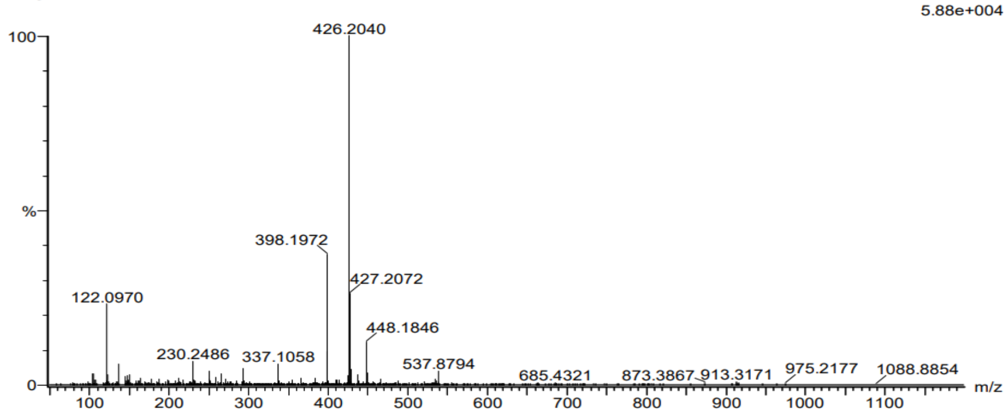

Minimum:				-1.5				
Maximum:		3000.0	2.0	50.0				
Mass	Calc. Mass	mDa	PPM	DBE	i-FIT	Norm	Conf(%)	Formula
426.2040	426.2042	-0.2	-0.5	16.5	438.2	0.000	99.99	C24 H24 N7 O
	426.2047	-0.7	-1.6	-1.5	447.1	8.885	0.01	C11 H32 N5 O12

Figure S21. HRMS (ES+, m/z) data of compound **21a**.

Compound 21b

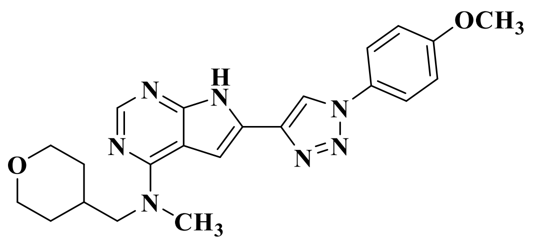

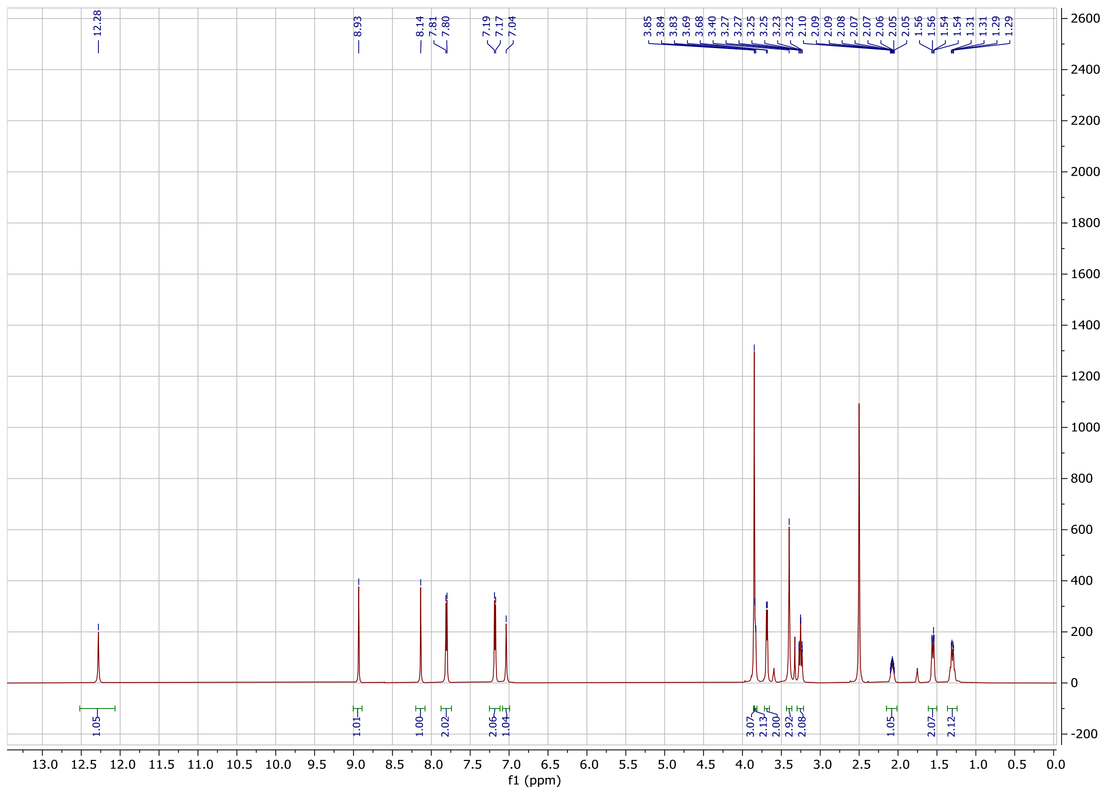

Figure S22. ^{1}H NMR (600 MHz, DMSO-d_6) spectrum of compound **21b**.

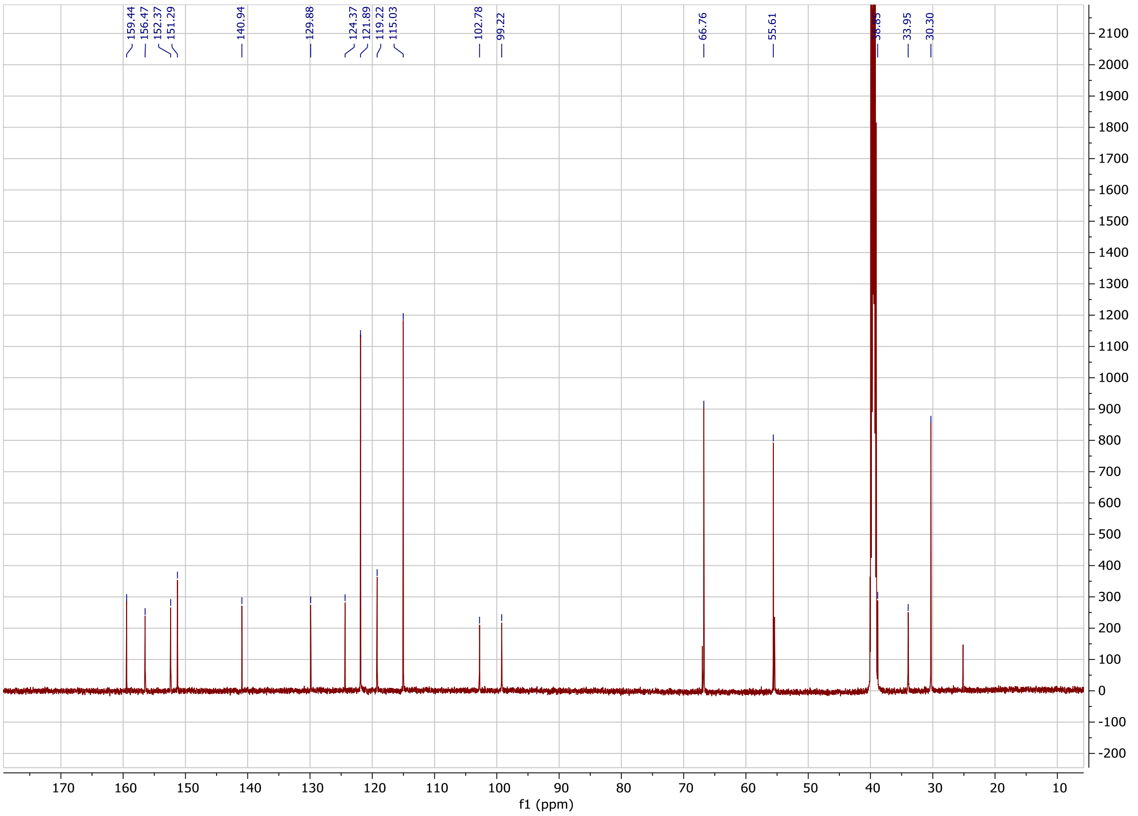

Figure S23. ^{13}C NMR (151 MHz, DMSO-d_6) spectrum of compound **21b**.

Elemental Composition Report **Page 1**

Single Mass Analysis
Tolerance = 2.0 PPM / DBE: min = -1.5, max = 50.0
Element prediction: Off
Number of isotope peaks used for i-FIT = 3

Monoisotopic Mass, Even Electron Ions
816 formula(e) evaluated with 1 results within limits (all results (up to 1000) for each mass)
Elements Used:
C: 1-100 H: 1-150 N: 0-8 O: 0-12 I: 0-1
ReqID3710 58 (0.552) AM2 (Ar,35000.0,0.00,0.00); Cm (56:58)
1: TOF MS ES+

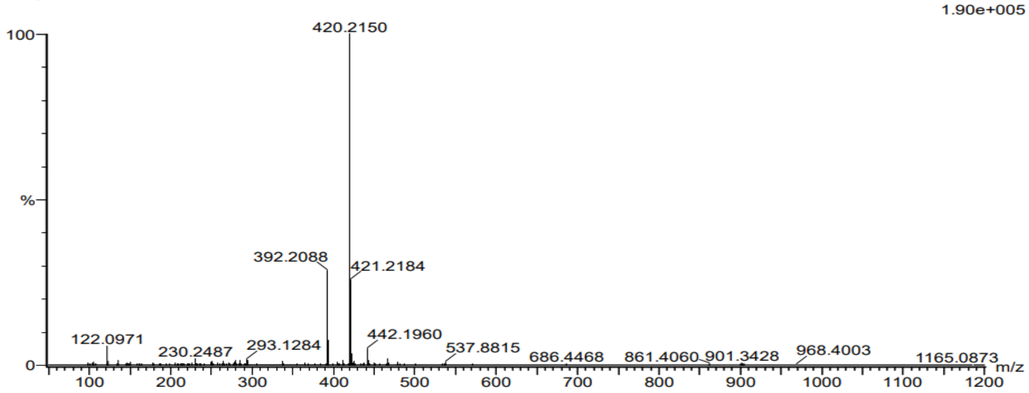

	Mass	Calc. Mass	mDa	PPM	DBE	i-FIT	Norm	Conf(%)	Formula
Minimum:					-1.5				
Maximum:			3000.0	2.0	50.0				
	420.2150	420.2148	0.2	0.5	13.5	604.2	n/a	n/a	C22 H26 N7 O2

Figure S24. HRMS (ES+, m/z) data of compound **21b**.

Compound 22a

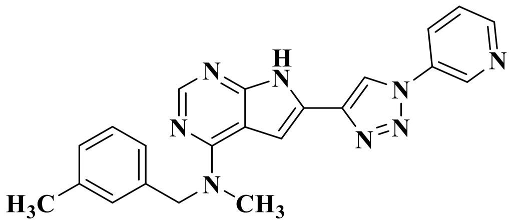

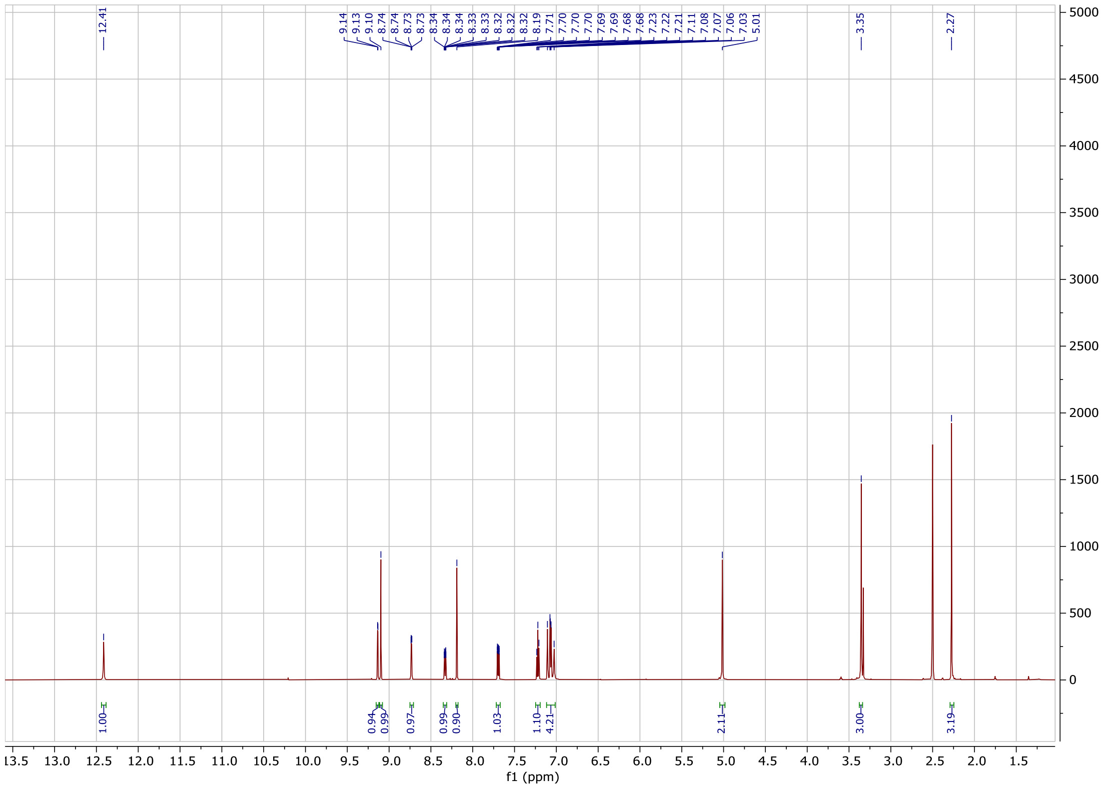

Figure S25. ^{1}H NMR (600 MHz, DMSO-d_6) spectrum of compound **22a**.

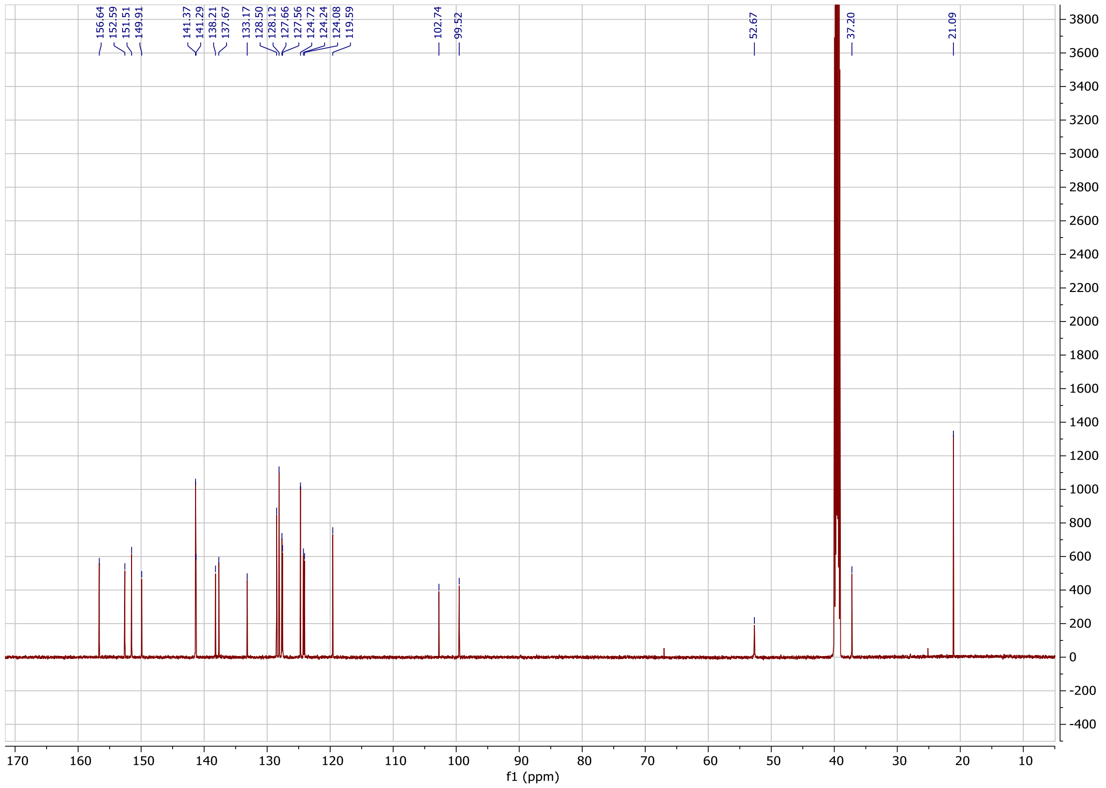

Figure S26. ^{13}C NMR (151 MHz, DMSO-d_6) spectrum of compound **22a**.

Elemental Composition Report Page 1

Single Mass Analysis
Tolerance = 2.0 PPM / DBE: min = -1.5, max = 50.0
Element prediction: Off
Number of isotope peaks used for i-FIT = 3

Monoisotopic Mass, Even Electron Ions
751 formula(e) evaluated with 2 results within limits (all results (up to 1000) for each mass)
Elements Used:
C: 1-100 H: 1-150 N: 0-8 O: 0-12 I: 0-1
ReqID3711 61 (0.586) AM2 (Ar,35000.0,0.00,0.00); Cm (58:61)
1: TOF MS ES+

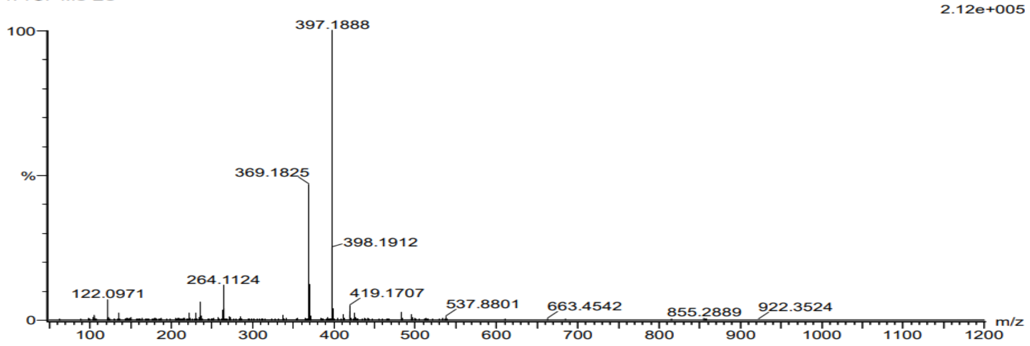

Minimum:				-1.5				
Maximum:		3000.0	2.0	50.0				
Mass	Calc. Mass	mDa	PPM	DBE	i-FIT	Norm	Conf(%)	Formula
397.1888	397.1889	-0.1	-0.3	16.5	692.2	0.000	99.96	C22 H21 N8
	397.1894	-0.6	-1.5	-1.5	700.0	7.805	0.04	C9 H29 N6 O11

Figure S27. HRMS (ES+, m/z) data of compound **22a**.

Compound 22b

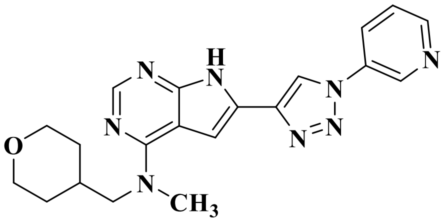

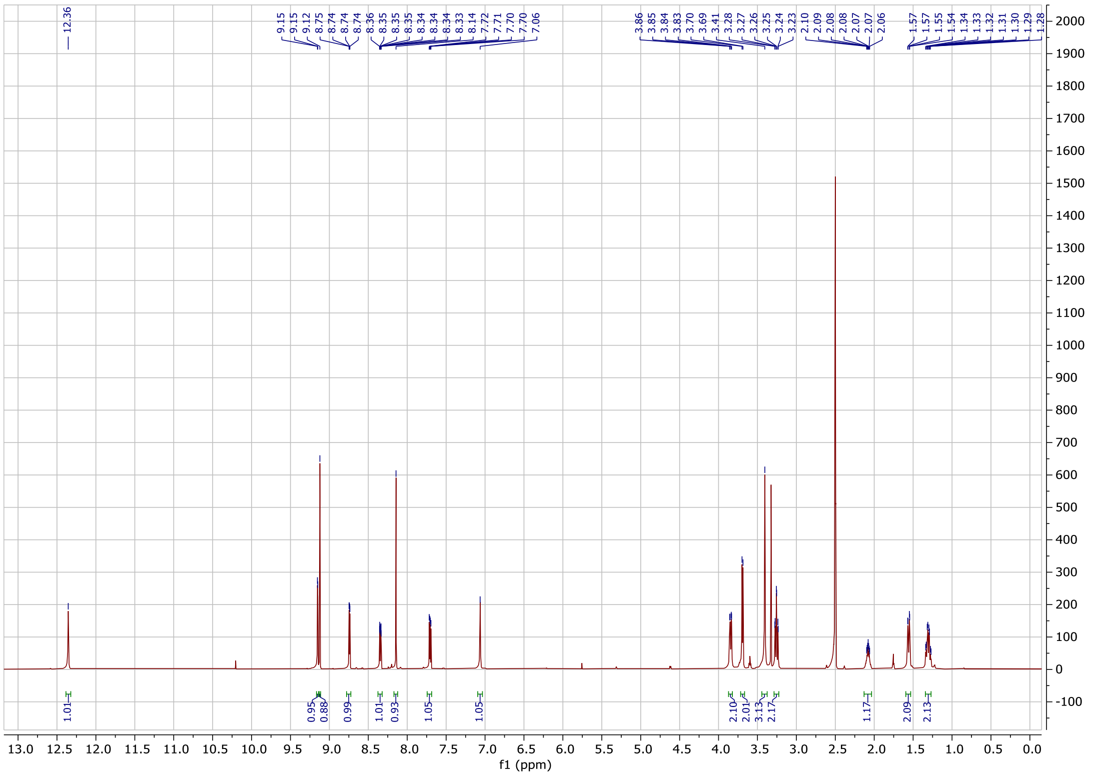

Figure S28. ^{1}H NMR (600 MHz, DMSO-d_6) spectrum of compound **22b**.

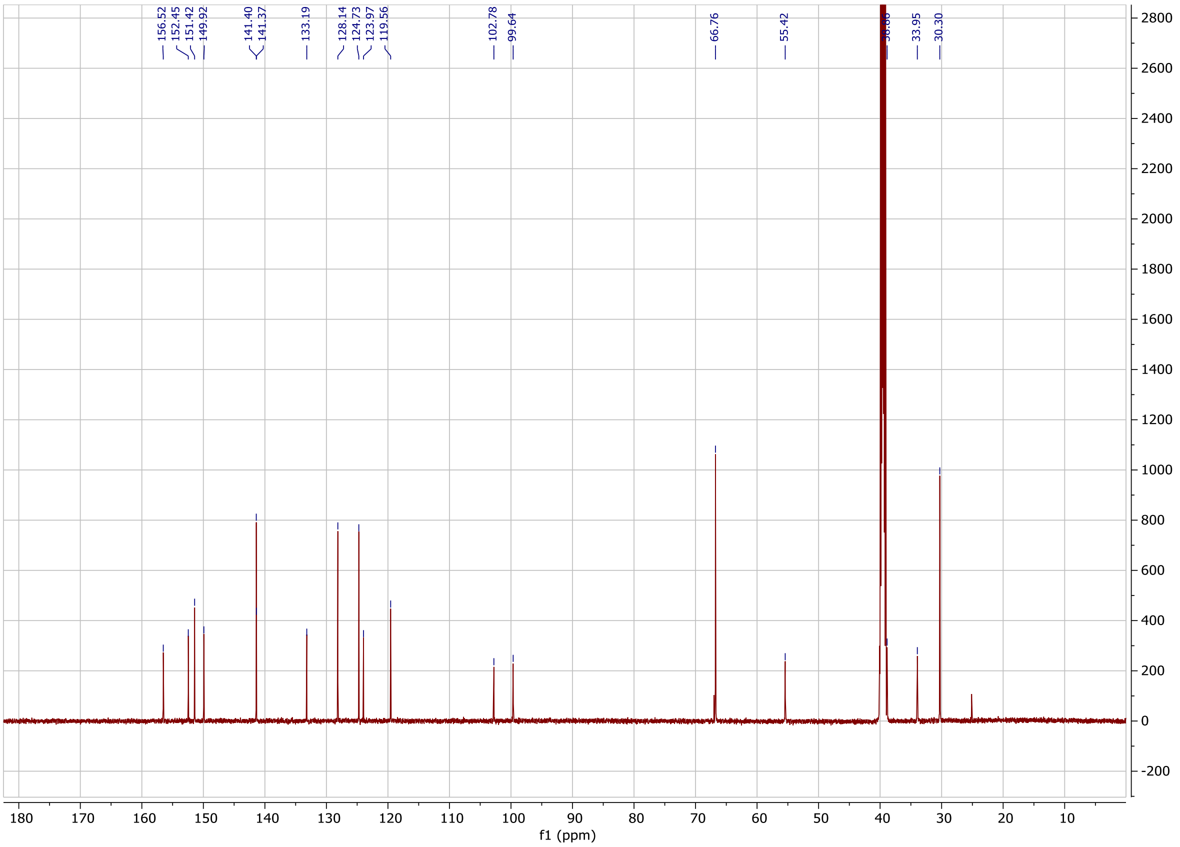

Figure S29. ^{13}C NMR (151 MHz, DMSO-d_6) spectrum of compound **22b**.

Elemental Composition Report **Page 1**

Single Mass Analysis
Tolerance = 2.0 PPM / DBE: min = -1.5, max = 50.0
Element prediction: Off
Number of isotope peaks used for i-FIT = 3

Monoisotopic Mass, Even Electron Ions
726 formula(e) evaluated with 1 results within limits (all results (up to 1000) for each mass)
Elements Used:
C: 1-100 H: 1-150 N: 0-8 O: 0-12 I: 0-1
ReqID3712 65 (0.622) AM2 (Ar,35000.0,0.00,0.00); Cm (62:65)
1: TOF MS ES+

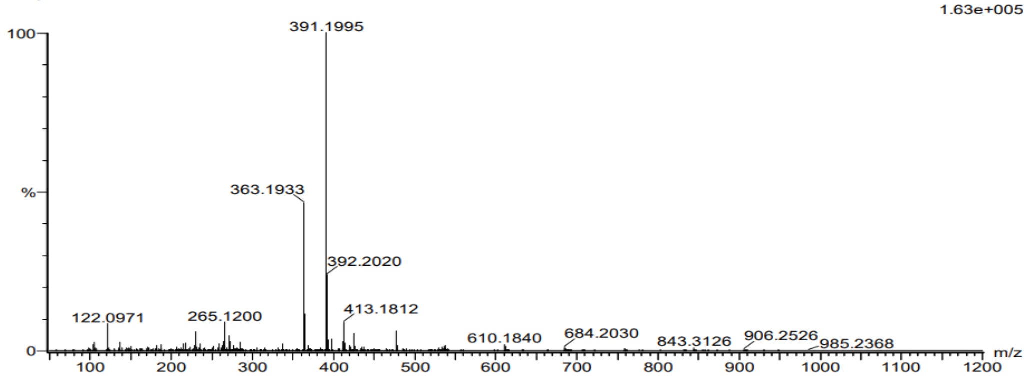

Minimum:				-1.5				
Maximum:		3000.0	2.0	50.0				
Mass	Calc. Mass	mDa	PPM	DBE	i-FIT	Norm	Conf(%)	Formula
391.1995	391.1995	0.0	0.0	13.5	637.7	n/a	n/a	C20 H23 N8 O

Figure S30. HRMS (ES+, m/z) data of compound **22b**.

Compound 23a

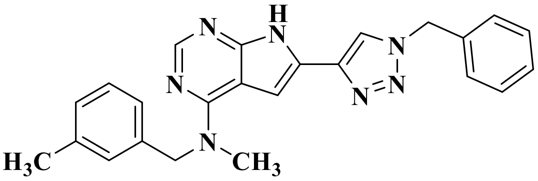

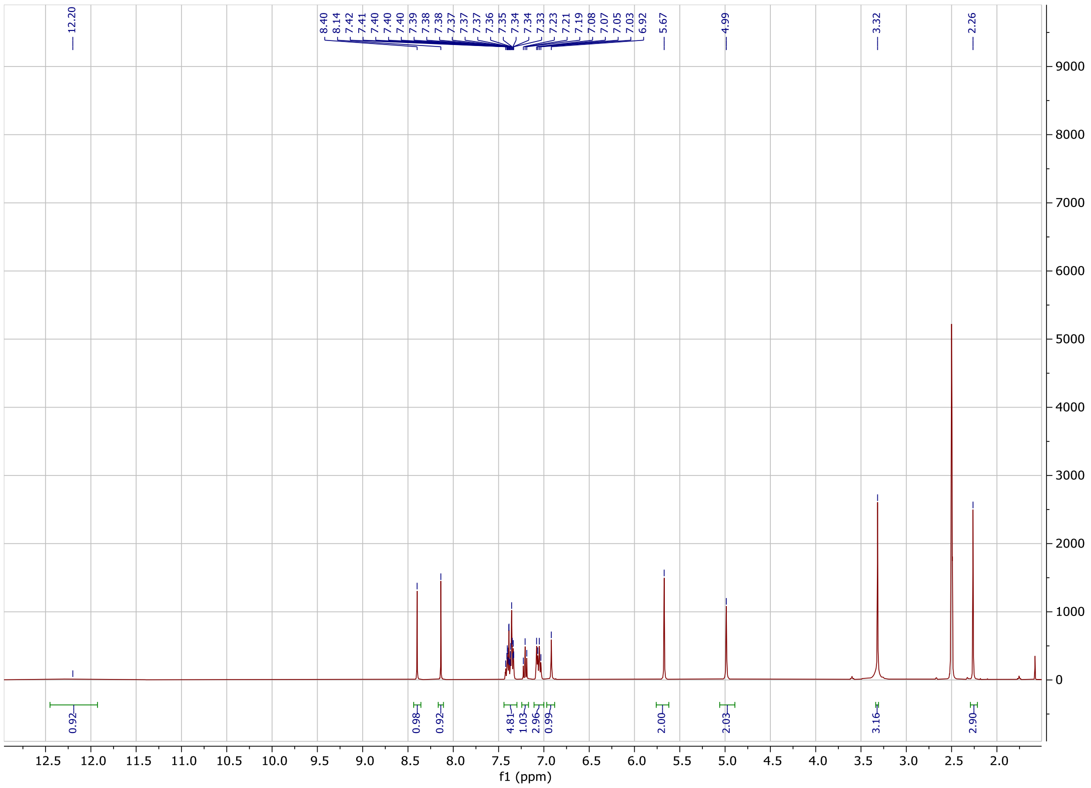

Figure S31. ^{1}H NMR (400 MHz, DMSO-d_6) spectrum of compound **23a**.

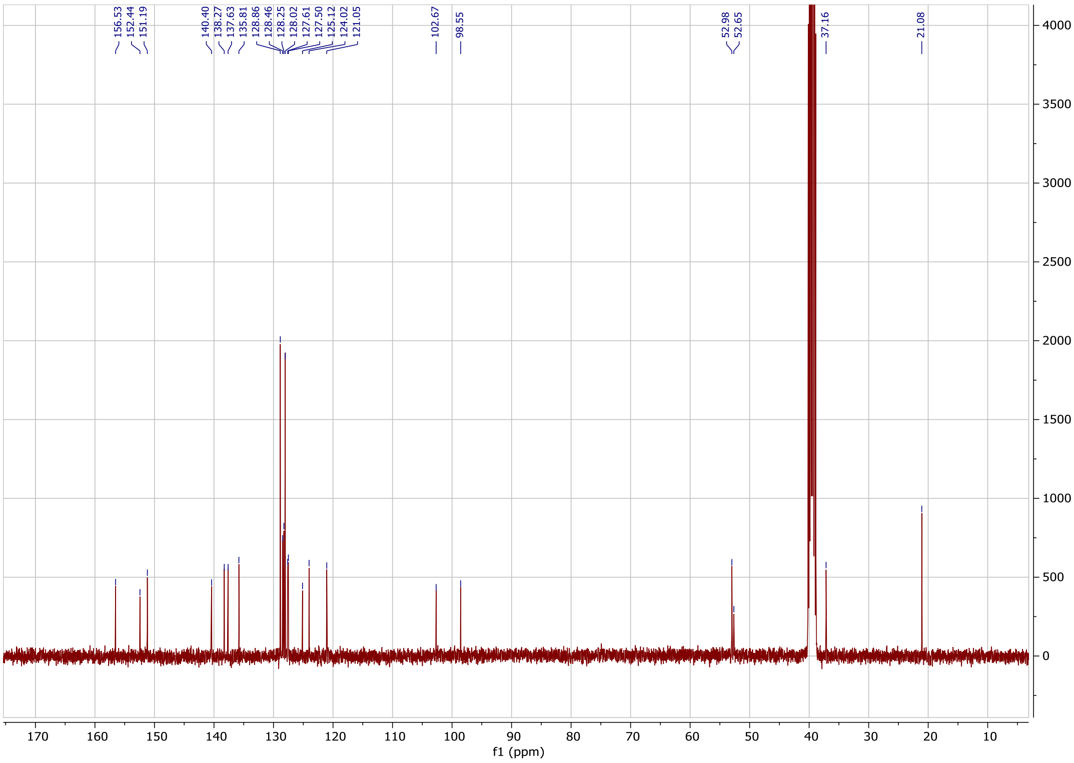

Figure S32. ^{13}C NMR (101 MHz, DMSO-d_6) spectrum of compound **23a**.

Elemental Composition Report **Page 1**

Single Mass Analysis
Tolerance = 2.0 PPM / DBE: min = -1.5, max = 50.0
Element prediction: Off
Number of isotope peaks used for i-FIT = 3

Monoisotopic Mass, Even Electron Ions
789 formula(e) evaluated with 2 results within limits (all results (up to 1000) for each mass)
Elements Used:
C: 1-100 H: 1-150 N: 0-8 O: 0-12 I: 0-1
ReqID3715 58 (0.552) AM2 (Ar,35000.0,0.00,0.00); Cm (54:58)
1: TOF MS ES+

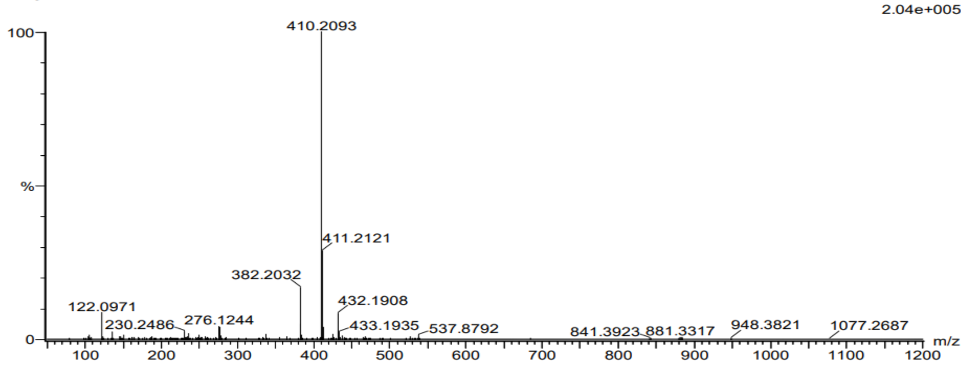

Minimum:				-1.5				
Maximum:		3000.0	2.0	50.0				
Mass	Calc. Mass	mDa	PPM	DBE	i-FIT	Norm	Conf(%)	Formula
410.2093	410.2093	0.0	0.0	16.5	637.6	0.000	100.00	C24 H24 N7
	410.2098	-0.5	-1.2	-1.5	652.2	14.572	0.00	C11 H32 N5 O11

Figure S33. HRMS (ES+, m/z) data of compound **23a**.

Compound 23b

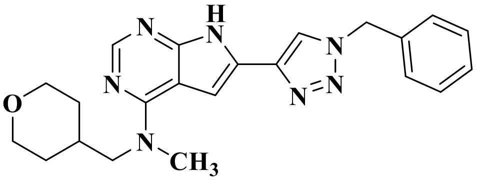

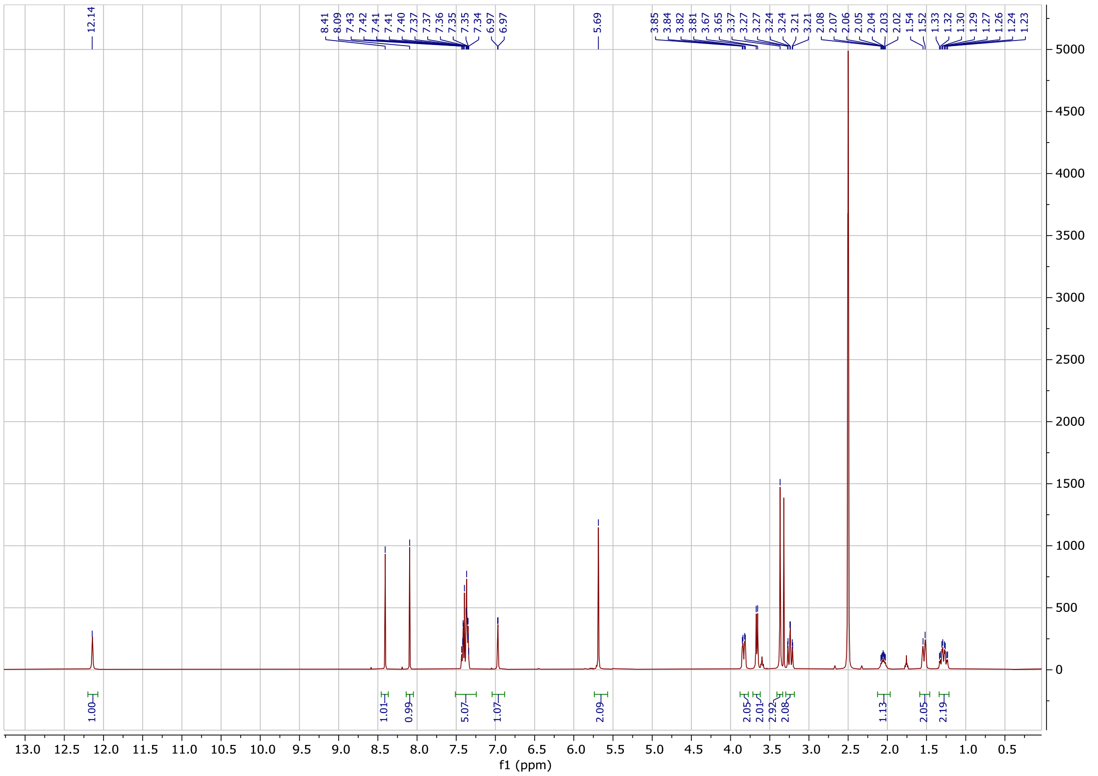

Figure S34. ^1^H NMR (400 MHz, DMSO-d_6) spectrum of compound **23b**.

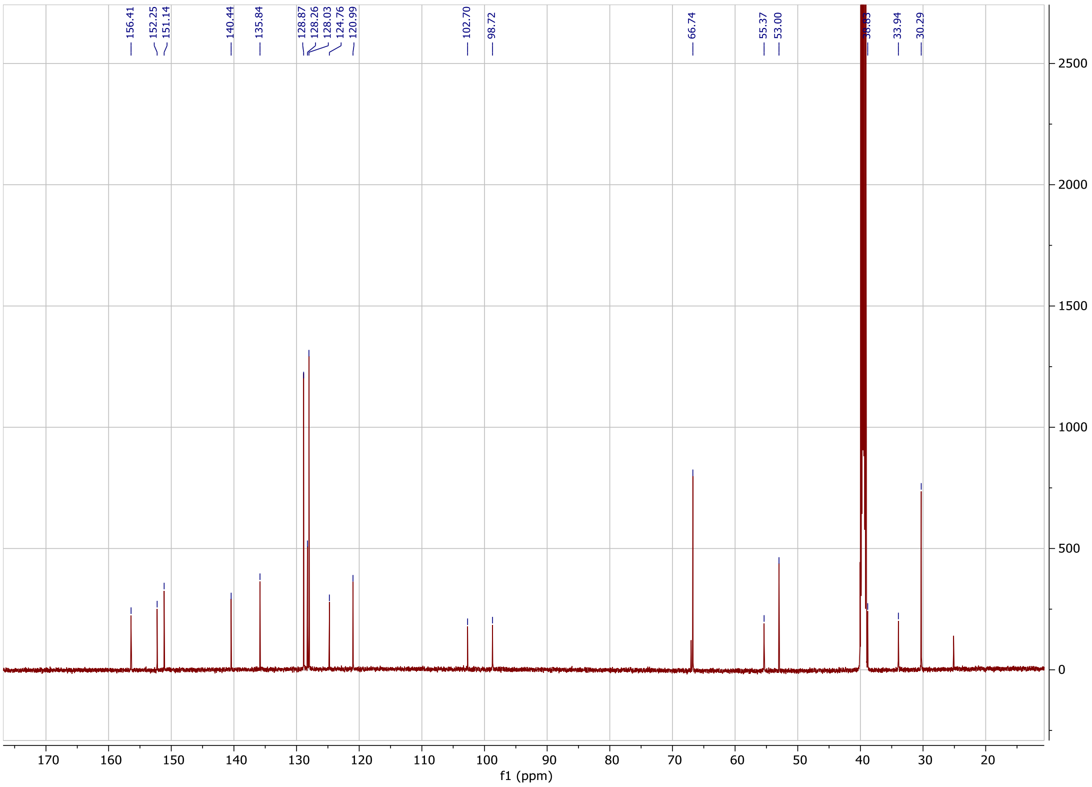

Figure S35. ^{13}C NMR (101 MHz, DMSO-d_6) spectrum of compound **23b**.

Elemental Composition Report **Page 1**

Single Mass Analysis
Tolerance = 2.0 PPM / DBE: min = -1.5, max = 50.0
Element prediction: Off
Number of isotope peaks used for i-FIT = 3

Monoisotopic Mass, Even Electron Ions
764 formula(e) evaluated with 1 results within limits (all results (up to 1000) for each mass)
Elements Used:
C: 1-100 H: 1-150 N: 0-8 O: 0-12 I: 0-1

ReqID3732 60 (0.577) AM2 (Ar,35000.0,0.00,0.00); Cm (59:60)
1: TOF MS ES+

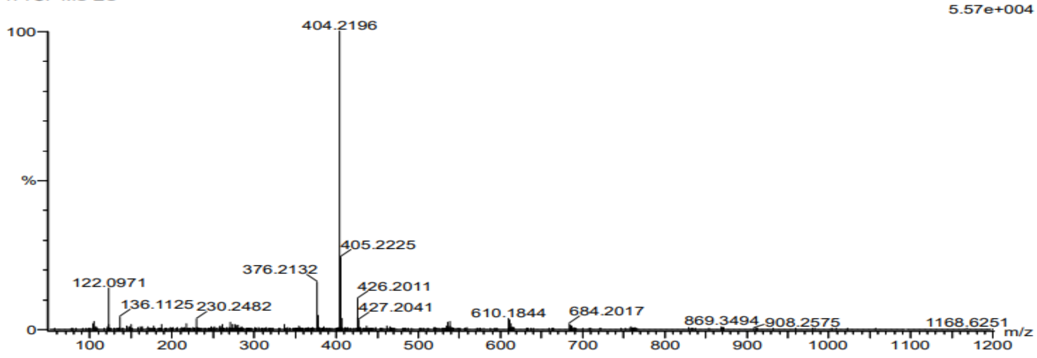

Minimum:				-1.5				
Maximum:		3000.0	2.0	50.0				
Mass	Calc. Mass	mDa	PPM	DBE	i-FIT	Norm	Conf(%)	Formula
404.2196	404.2199	-0.3	-0.7	13.5	419.3	n/a	n/a	C22 H26 N7 O

Figure S36. HRMS (ES+, m/z) data of compound **23b**.

Compound 24a

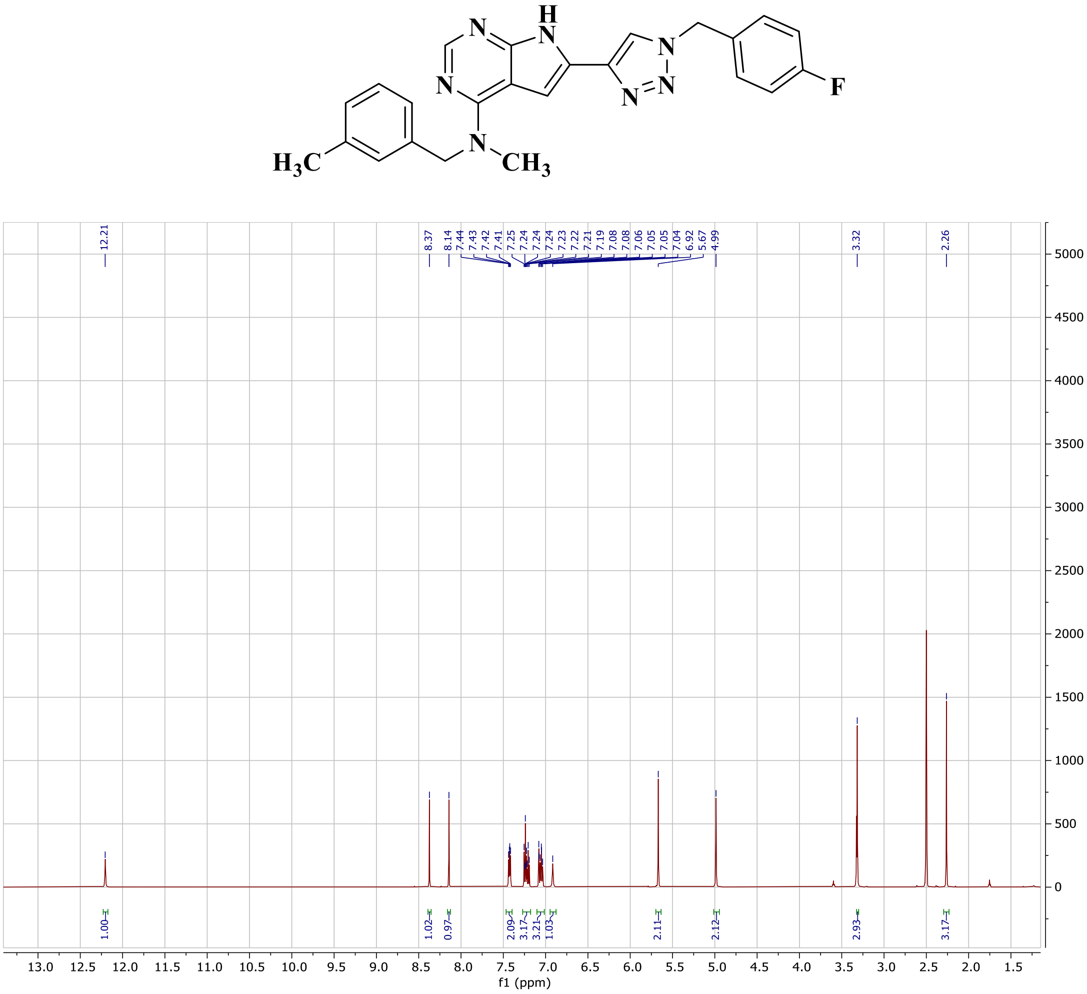

Figure S37. ^{1}H NMR (600 MHz, DMSO-d_6) spectrum of compound **24a**.

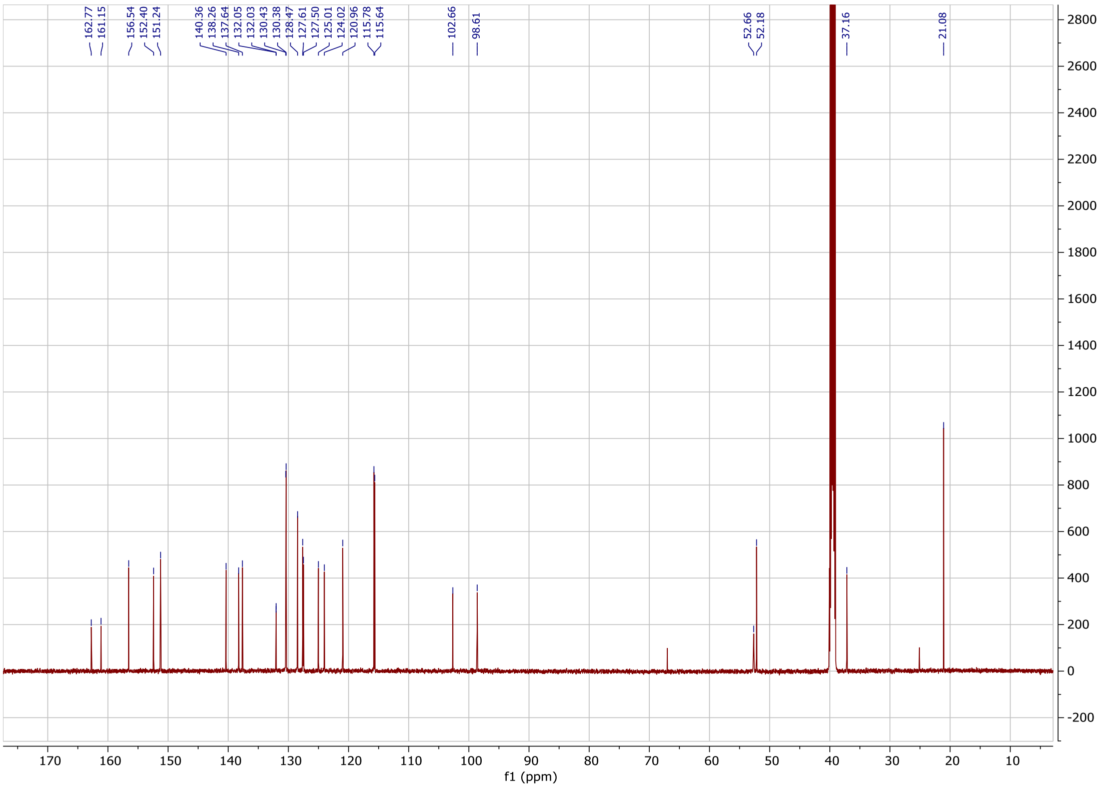

Figure S38. ^{13}C NMR (151 MHz, DMSO-d_6) spectrum of compound **24a**.

Elemental Composition Report **Page 1**

Single Mass Analysis
Tolerance = 2.0 PPM / DBE: min = -1.5, max = 50.0
Element prediction: Off
Number of isotope peaks used for i-FIT = 3

Monoisotopic Mass, Even Electron Ions
2917 formula(e) evaluated with 6 results within limits (all results (up to 1000) for each mass)
Elements Used:
C: 1-100 H: 1-150 N: 0-8 O: 0-12 F: 0-3 I: 0-1

ReqID3730 58 (0.552) AM2 (Ar,35000.0,0.00,0.00); Cm (56:58)
1: TOF MS ES+

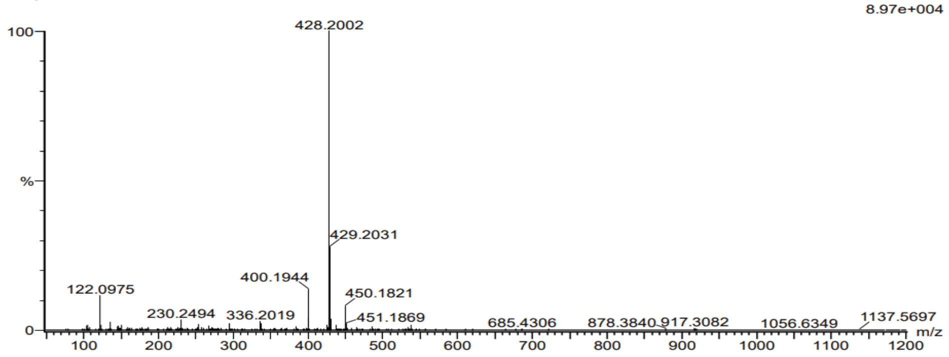

Mass	Calc. Mass	mDa	PPM	DBE	i-FIT	Norm	Conf(%)	Formula
Minimum:				-1.5				
Maximum:		3000.0	2.0	50.0				
428.2002	428.1999	0.3	0.7	16.5	484.5	0.065	93.72	C24 H23 N7 F
	428.2010	-0.8	-1.9	12.5	487.4	2.939	5.29	C21 H24 N7 O F2
	428.1997	0.5	1.2	7.5	489.1	4.662	0.95	C20 H28 N3 O5 F2
	428.2008	-0.6	-1.4	3.5	492.3	7.897	0.04	C17 H29 N3 O6 F3
	428.1999	0.3	0.7	0.5	494.0	9.558	0.01	C14 H35 N7 I
	428.2004	-0.2	-0.5	-1.5	494.9	10.506	0.00	C11 H31 N5 O11 F

Figure S39. HRMS (ES+, m/z) data of compound **24a**.

Compound 24b

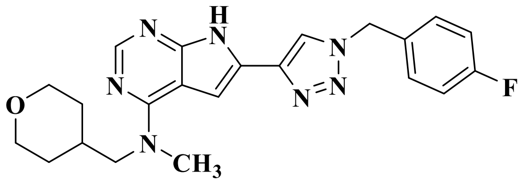

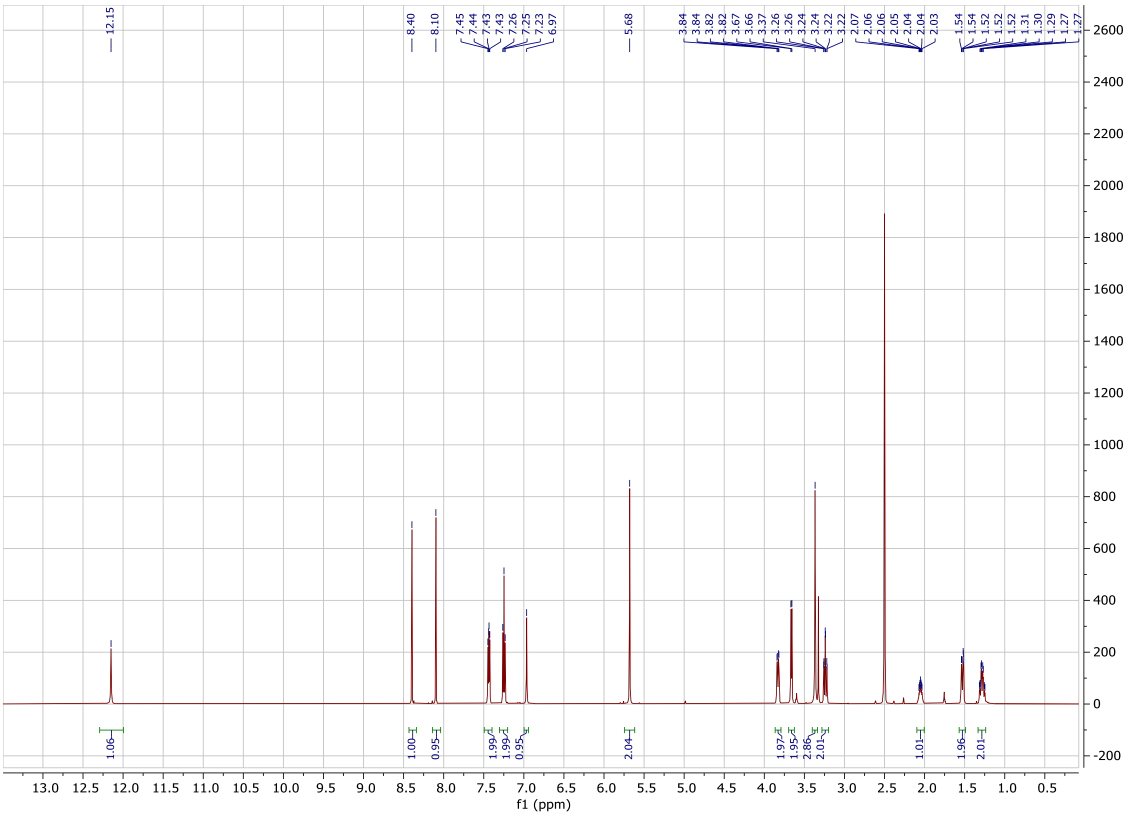

Figure S40. ^{1}H NMR (600 MHz, DMSO-d_6) spectrum of compound **24b**.

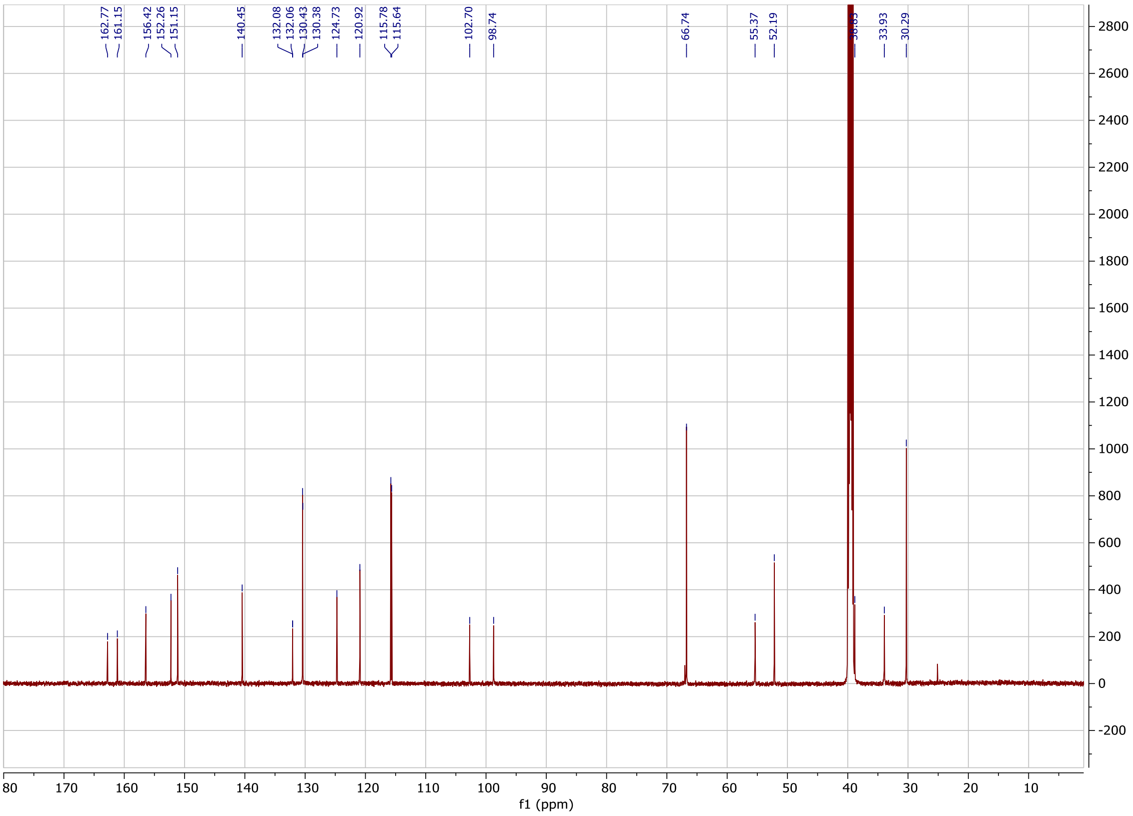

Figure S41. ^{13}C NMR (151 MHz, DMSO-d_6) spectrum of compound **24b**.

Elemental Composition Report **Page 1**

Single Mass Analysis
Tolerance = 2.0 PPM / DBE: min = -1.5, max = 50.0
Element prediction: Off
Number of isotope peaks used for i-FIT = 3

Monoisotopic Mass, Even Electron Ions
2840 formula(e) evaluated with 4 results within limits (all results (up to 1000) for each mass)
Elements Used:
C: 1-100 H: 1-150 N: 0-8 O: 0-12 F: 0-3 I: 0-1
ReqID3725 60 (0.577) AM2 (Ar,35000.0,0.00,0.00); Cm (59:60)
1: TOF MS ES+

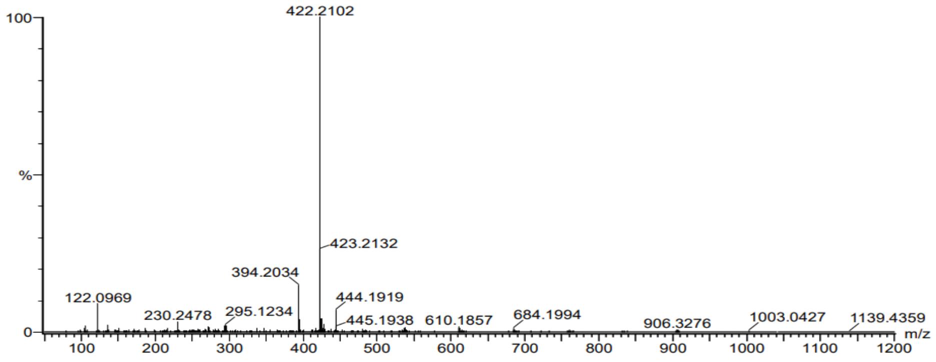

Minimum:				-1.5				
Maximum:		3000.0	2.0	50.0				
Mass	Calc. Mass	mDa	PPM	DBE	i-FIT	Norm	Conf(%)	Formula
422.2102	422.2105	-0.3	-0.7	13.5	479.7	0.091	91.33	C22 H25 N7 O F
	422.2096	0.6	1.4	13.5	482.0	2.469	8.47	C27 H27 N F3
	422.2103	-0.1	-0.2	4.5	485.8	6.226	0.20	C18 H30 N3 O6 F2
	422.2098	0.4	0.9	-0.5	491.0	11.407	0.00	C12 H32 N5 O11

Figure S42. HRMS (ES+, m/z) data of compound **24b**.

Compound 25a

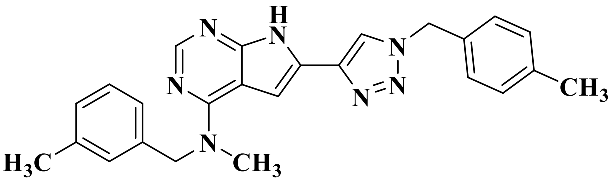

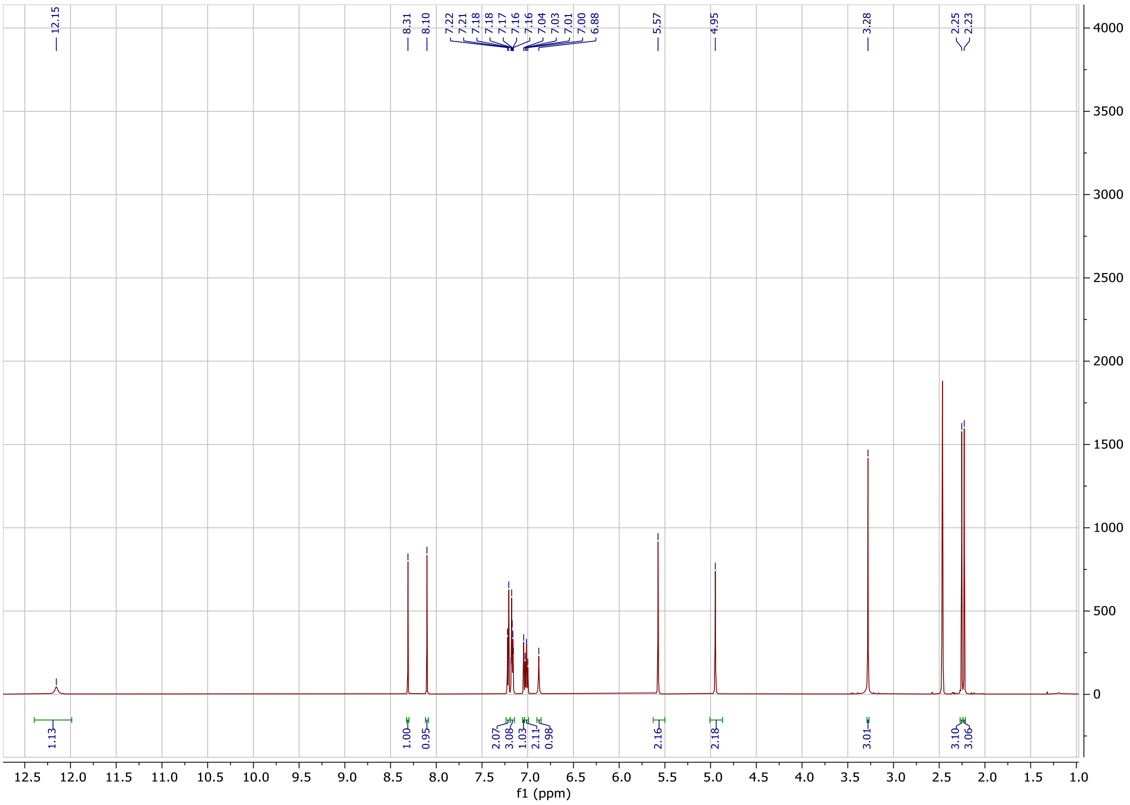

Figure S43. ^{1}H NMR (600 MHz, DMSO-d_6) spectrum of compound **25a**.

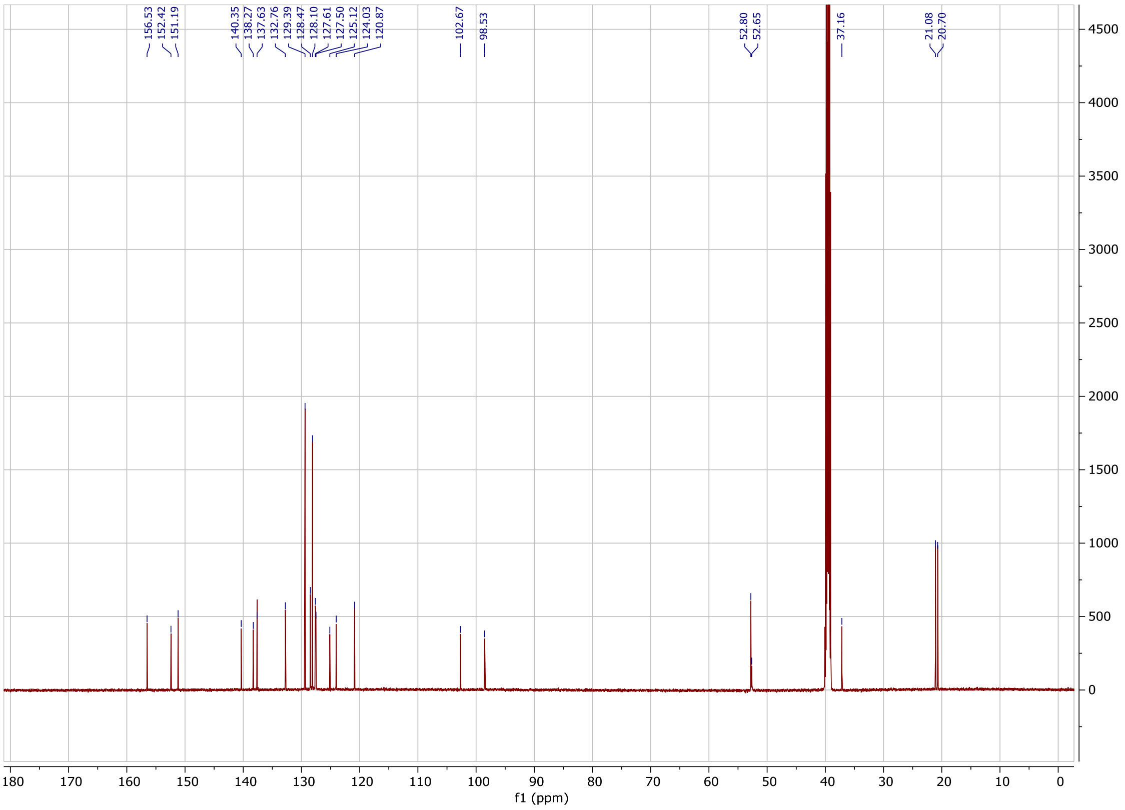

Figure S44. ^{13}C NMR (151 MHz, DMSO-d_6) spectrum of compound **25a**.

Elemental Composition Report **Page 1**

Single Mass Analysis
Tolerance = 2.0 PPM / DBE: min = -1.5, max = 50.0
Element prediction: Off
Number of isotope peaks used for i-FIT = 3

Monoisotopic Mass, Even Electron Ions
831 formula(e) evaluated with 2 results within limits (all results (up to 1000) for each mass)
Elements Used:
C: 1-100 H: 1-150 N: 0-8 O: 0-12 I: 0-1
ReqID3726 59 (0.568) AM2 (Ar,35000.0,0.00,0.00); Cm (57:59)
1: TOF MS ES+

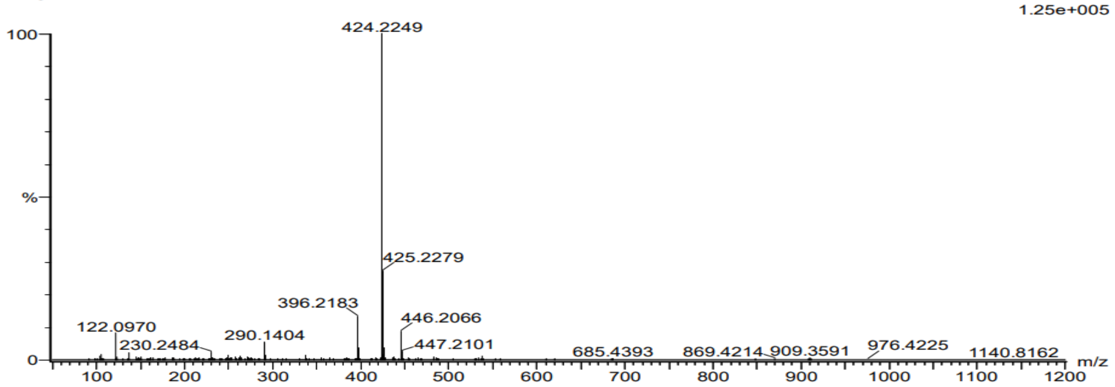

Minimum:				-1.5				
Maximum:		3000.0	2.0	50.0				
Mass	Calc. Mass	mDa	PPM	DBE	i-FIT	Norm	Conf(%)	Formula
424.2249	424.2250	-0.1	-0.2	16.5	557.2	0.006	99.40	C25 H26 N7
	424.2255	-0.6	-1.4	-1.5	562.3	5.113	0.60	C12 H34 N5 O11

Figure S45. HRMS (ES+, m/z) data of compound **25a**.

Compound 25b

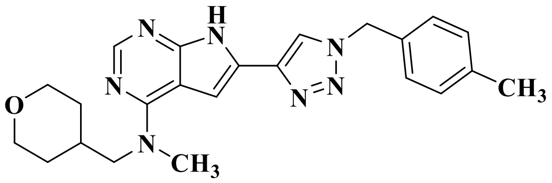

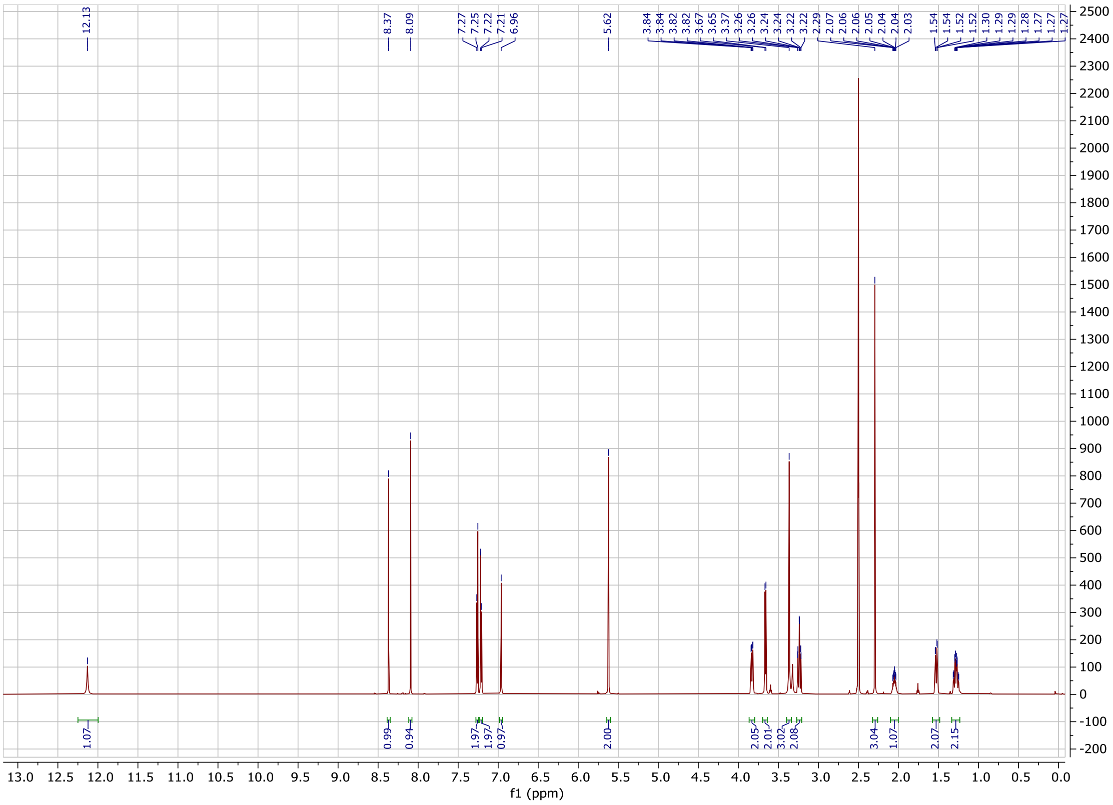

Figure S46. ^{1}H NMR (600 MHz, DMSO-d_6) spectrum of compound **25b**.

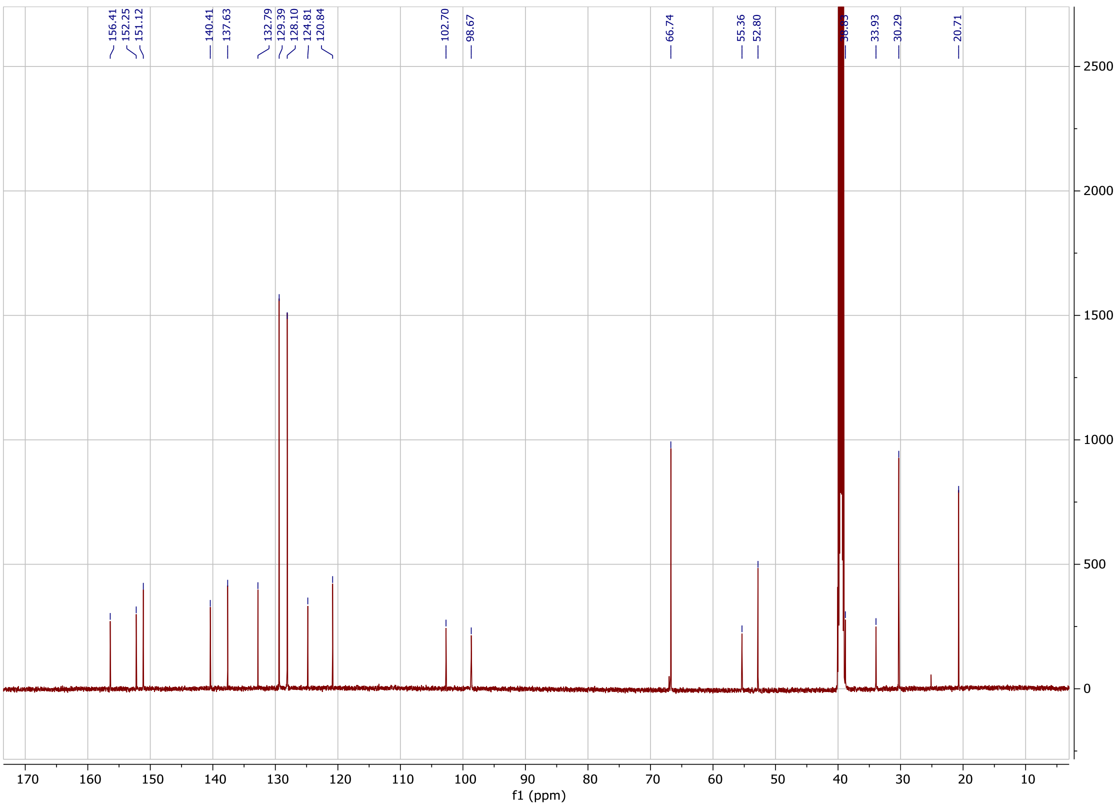

Figure S47. ^{13}C NMR (151 MHz, DMSO-d_6) spectrum of compound **25b**.

Elemental Composition Report **Page 1**

Single Mass Analysis
Tolerance = 2.0 PPM / DBE: min = -1.5, max = 50.0
Element prediction: Off
Number of isotope peaks used for i-FIT = 3

Monoisotopic Mass, Even Electron Ions
805 formula(e) evaluated with 1 results within limits (all results (up to 1000) for each mass)
Elements Used:
C: 1-100 H: 1-150 N: 0-8 O: 0-12 I: 0-1
ReqID3707 63 (0.603) AM2 (Ar,35000.0,0.00,0.00); Cm (62:63)
1: TOF MS ES+

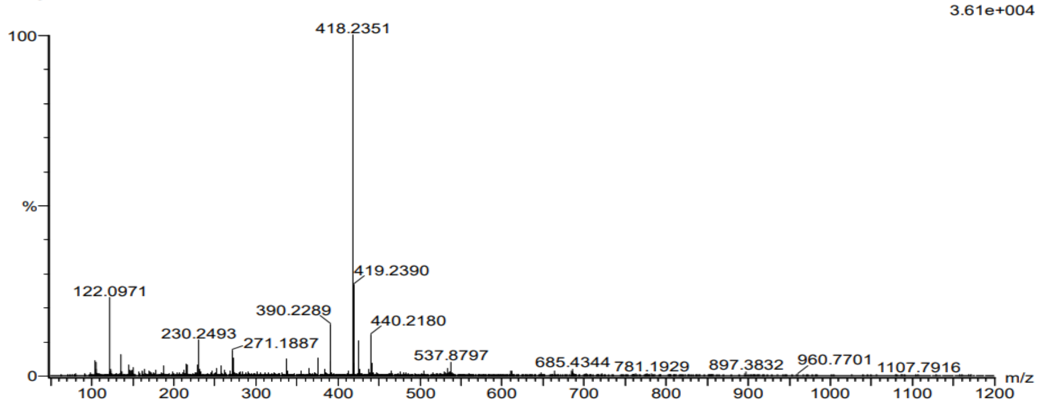

Minimum:				-1.5				
Maximum:		3000.0	2.0	50.0				
Mass	Calc. Mass	mDa	PPM	DBE	i-FIT	Norm	Conf(%)	Formula
418.2351	418.2355	-0.4	-1.0	13.5	308.5	n/a	n/a	C23 H28 N7 O

Figure S48. HRMS (ES+, m/z) data of compound **25b**.

Compound 26a

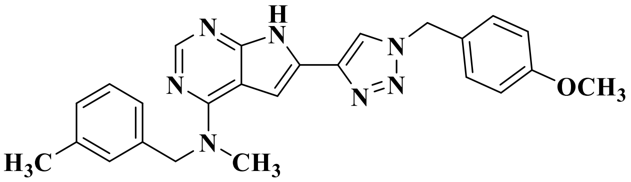

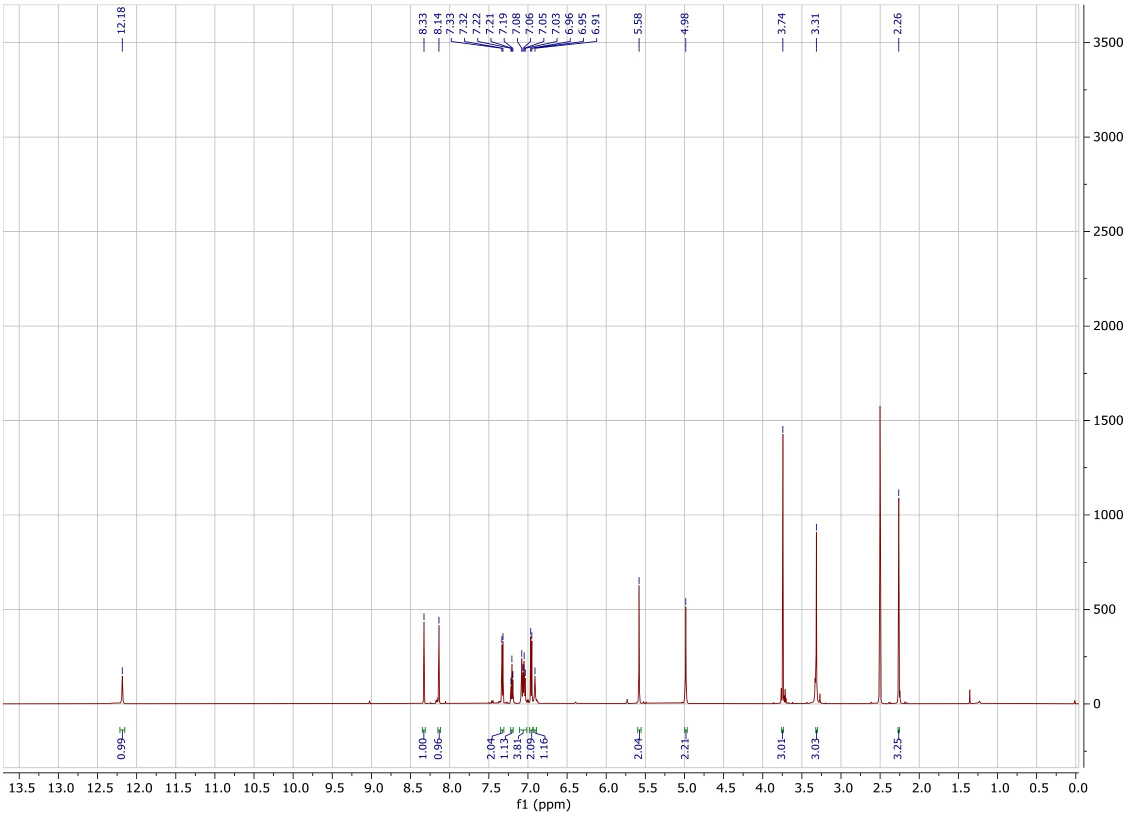

Figure S49. ^{1}H NMR (600 MHz, DMSO-d_6) spectrum of compound **26a**.

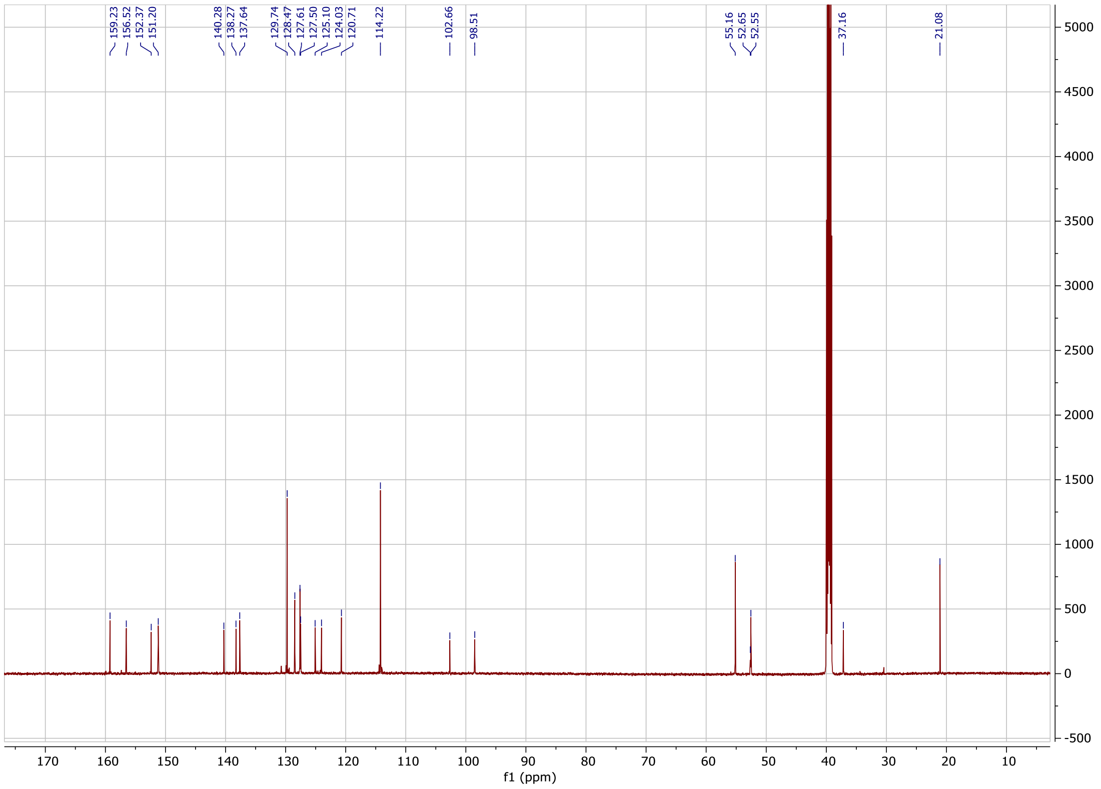

Figure S50. ^{13}C NMR (151 MHz, DMSO-d_6) spectrum of compound **26a**.

Elemental Composition Report **Page 1**

Single Mass Analysis
Tolerance = 2.0 PPM / DBE: min = -1.5, max = 50.0
Element prediction: Off
Number of isotope peaks used for i-FIT = 3

Monoisotopic Mass, Even Electron Ions
877 formula(e) evaluated with 2 results within limits (all results (up to 1000) for each mass)
Elements Used:
C: 1-100 H: 1-150 N: 0-8 O: 0-12 I: 0-1
ReqID3721 58 (0.552) AM2 (Ar,35000.0,0.00,0.00); Cm (57:58)
1: TOF MS ES+

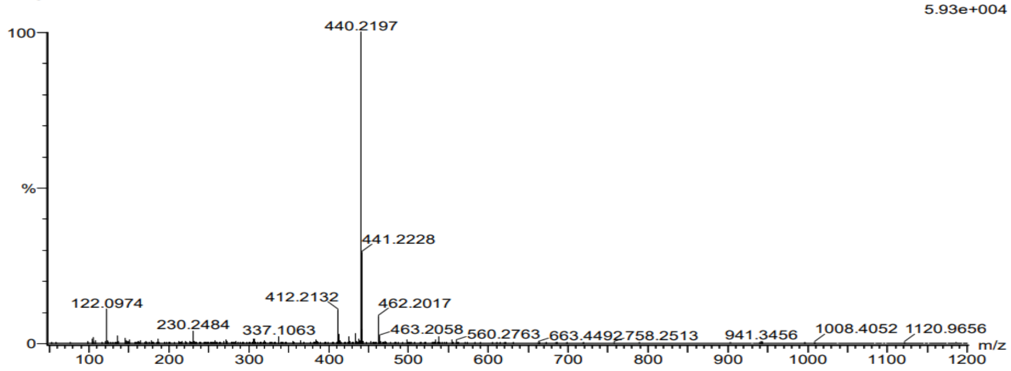

Minimum:				-1.5				
Maximum:		3000.0	2.0	50.0				
Mass	Calc. Mass	mDa	PPM	DBE	i-FIT	Norm	Conf(%)	Formula
440.2197	440.2199	-0.2	-0.5	16.5	431.3	0.000	100.00	C25 H26 N7 O
	440.2204	-0.7	-1.6	-1.5	442.9	11.648	0.00	C12 H34 N5 O12

Figure S51. HRMS (ES+, m/z) data of compound **26a**.

Compound 26b

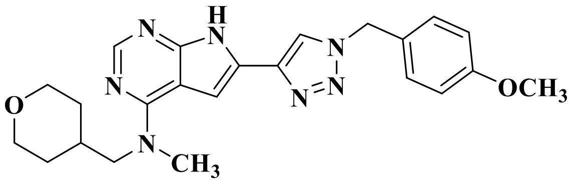

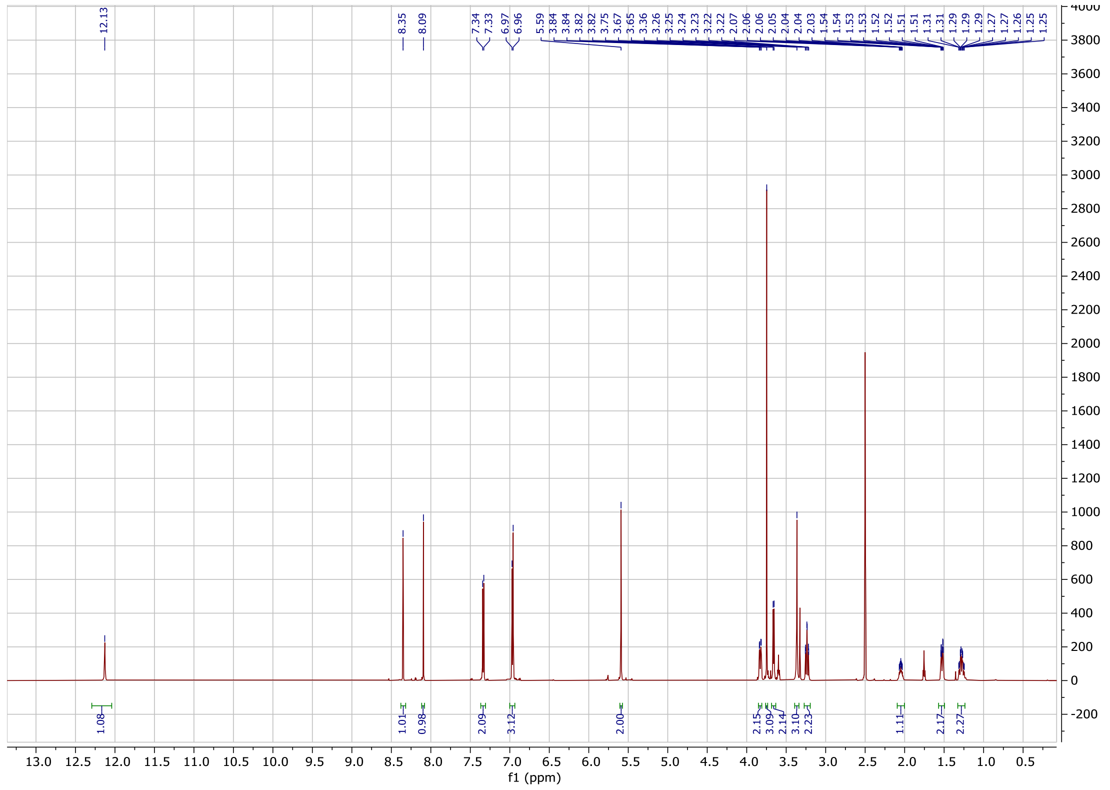

Figure S52. ^{1}H NMR (600 MHz, DMSO-d_6) spectrum of compound **26b**.

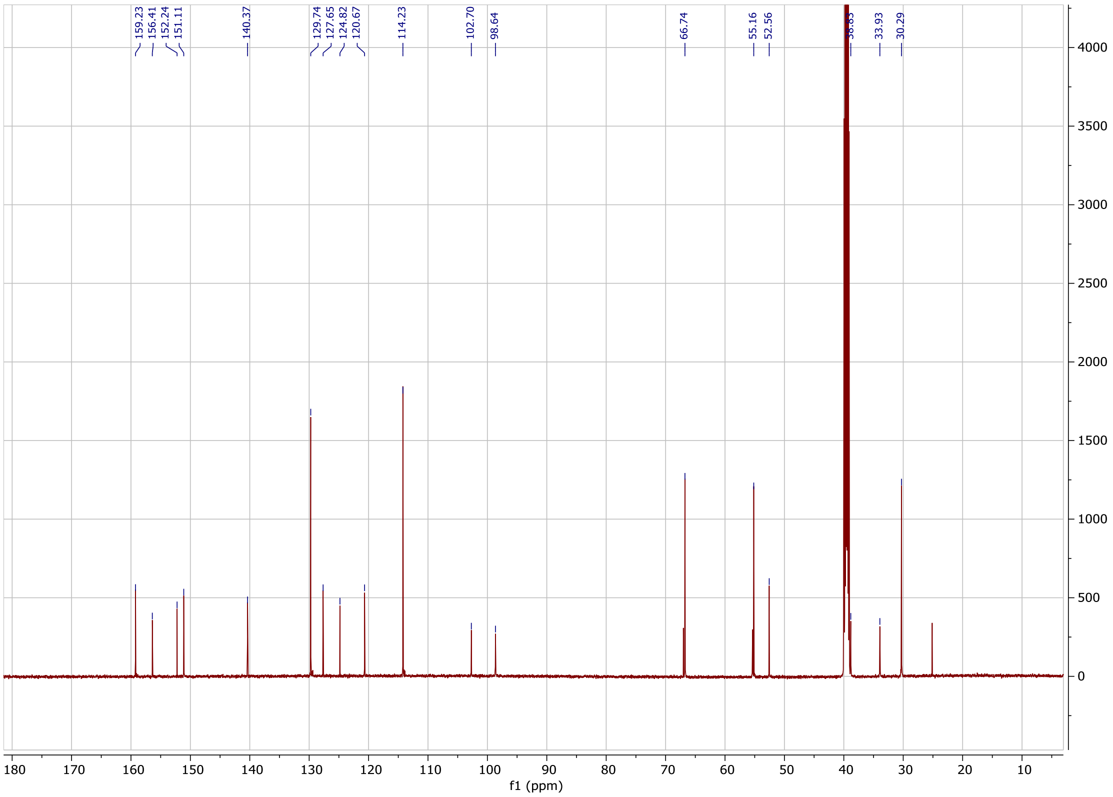

Figure S53. ^{13}C NMR (151 MHz, DMSO-d_6) spectrum of compound **26b**.

Elemental Composition Report **Page 1**

Single Mass Analysis
Tolerance = 2.0 PPM / DBE: min = -1.5, max = 50.0
Element prediction: Off
Number of isotope peaks used for i-FIT = 3

Monoisotopic Mass, Even Electron Ions
856 formula(e) evaluated with 1 results within limits (all results (up to 1000) for each mass)
Elements Used:
C: 1-100 H: 1-150 N: 0-8 O: 0-12 I: 0-1
ReqID3720 60 (0.577) AM2 (Ar,35000.0,0.00,0.00); Cm (58:60)
1: TOF MS ES+

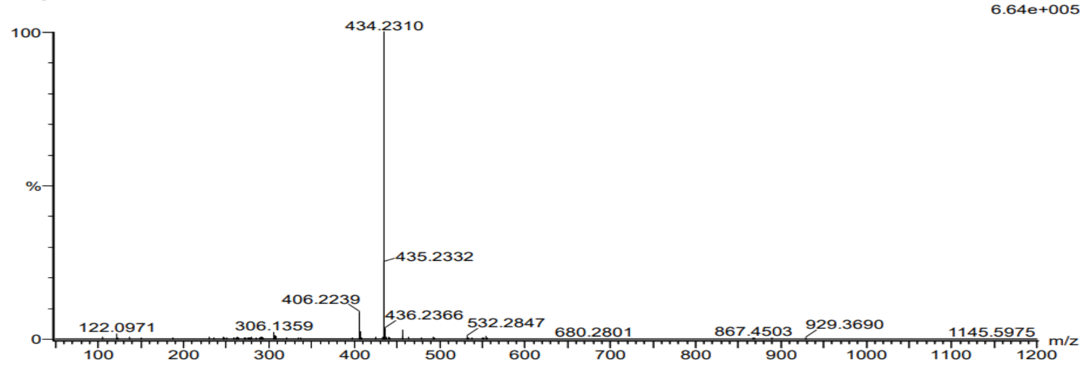

Minimum:				-1.5				
Maximum:		3000.0	2.0	50.0				
Mass	Calc. Mass	mDa	PPM	DBE	i-FIT	Norm	Conf(%)	Formula
434.2310	434.2304	0.6	1.4	13.5	766.6	n/a	n/a	C23 H28 N7 O2

Figure S54. HRMS (ES+, m/z) data of compound **26b**.

Compound 27a

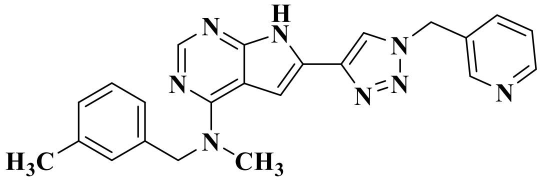

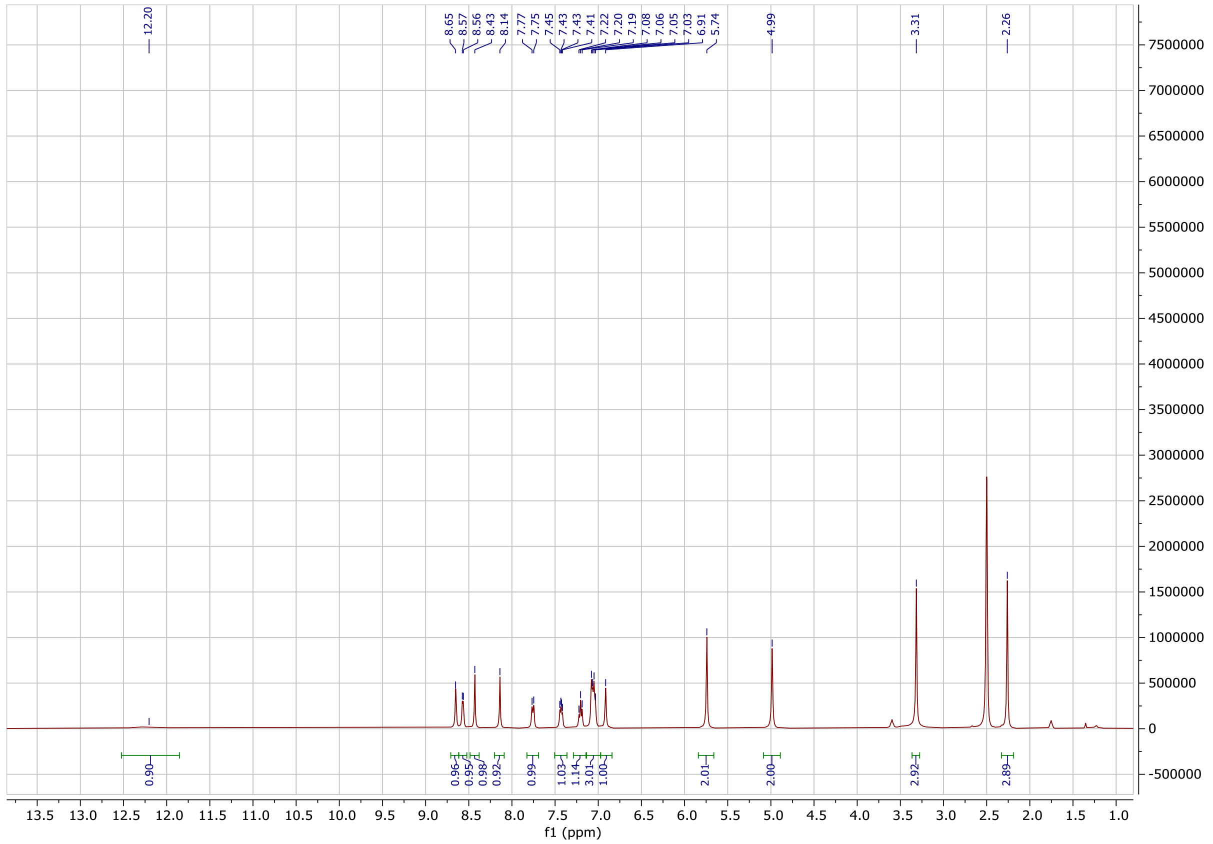

Figure S55. ^{1}H NMR (400 MHz, DMSO-d_6) spectrum of compound **27a**.

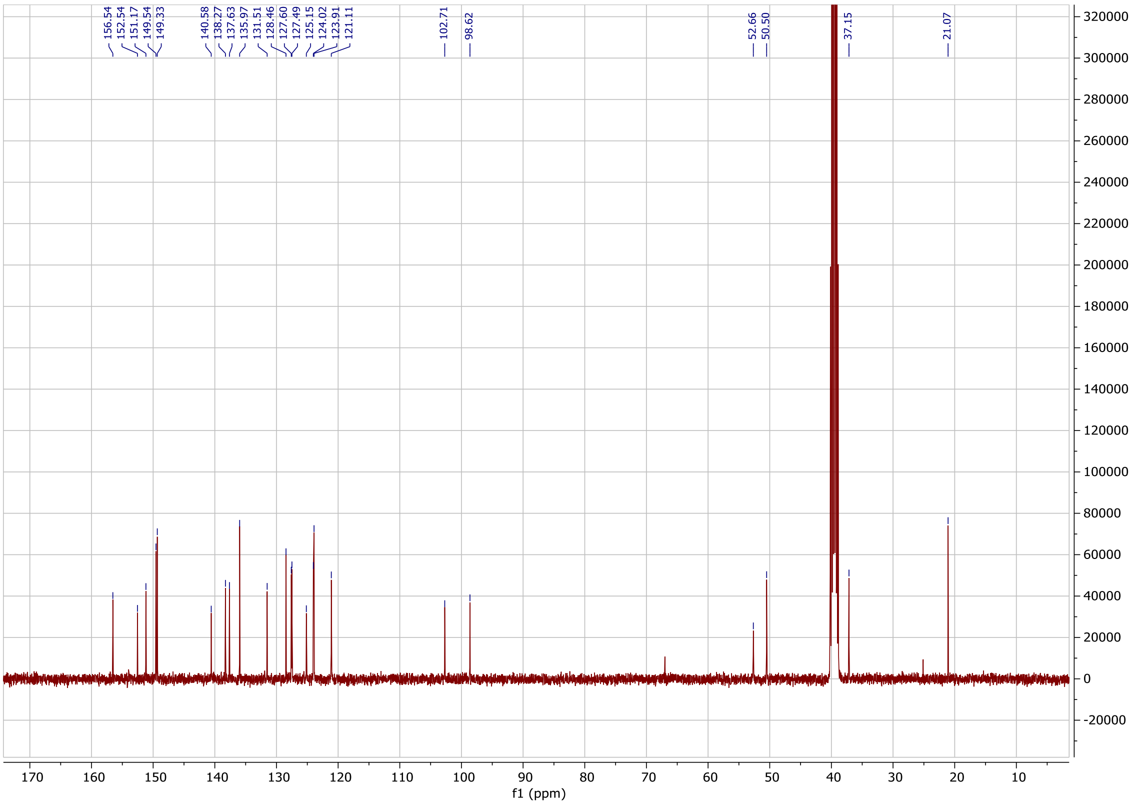

Figure S56. ^{13}C NMR (101 MHz, DMSO-d_6) spectrum of compound **27a**.

Elemental Composition Report **Page 1**

Single Mass Analysis
Tolerance = 2.0 PPM / DBE: min = -1.5, max = 50.0
Element prediction: Off
Number of isotope peaks used for i-FIT = 3

Monoisotopic Mass, Even Electron Ions
790 formula(e) evaluated with 2 results within limits (all results (up to 1000) for each mass)
Elements Used:
C: 1-100 H: 1-150 N: 0-8 O: 0-12 I: 0-1
ReqID3723 74 (0.708) AM2 (Ar,35000.0,0.00,0.00); Cm (72:74)
1: TOF MS ES+

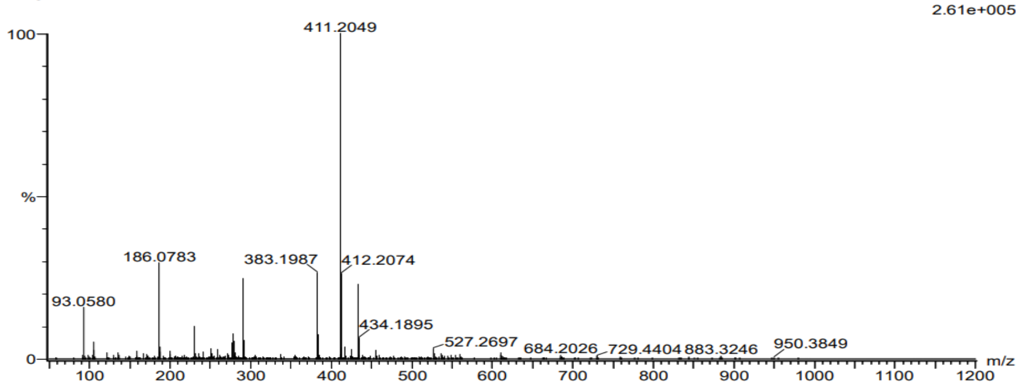

Minimum:				-1.5				
Maximum:		3000.0	2.0	50.0				
Mass	Calc. Mass	mDa	PPM	DBE	i-FIT	Norm	Conf(%)	Formula
411.2049	411.2046	0.3	0.7	16.5	721.0	0.000	99.99	C23 H23 N8
	411.2051	-0.2	-0.5	-1.5	730.2	9.153	0.01	C10 H31 N6 O11

Figure S57. HRMS (ES+, m/z) data of compound **27a**.

Compound 27b

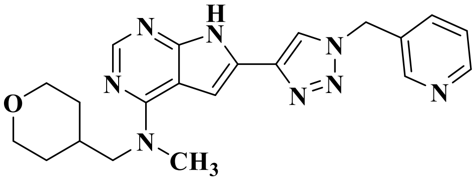

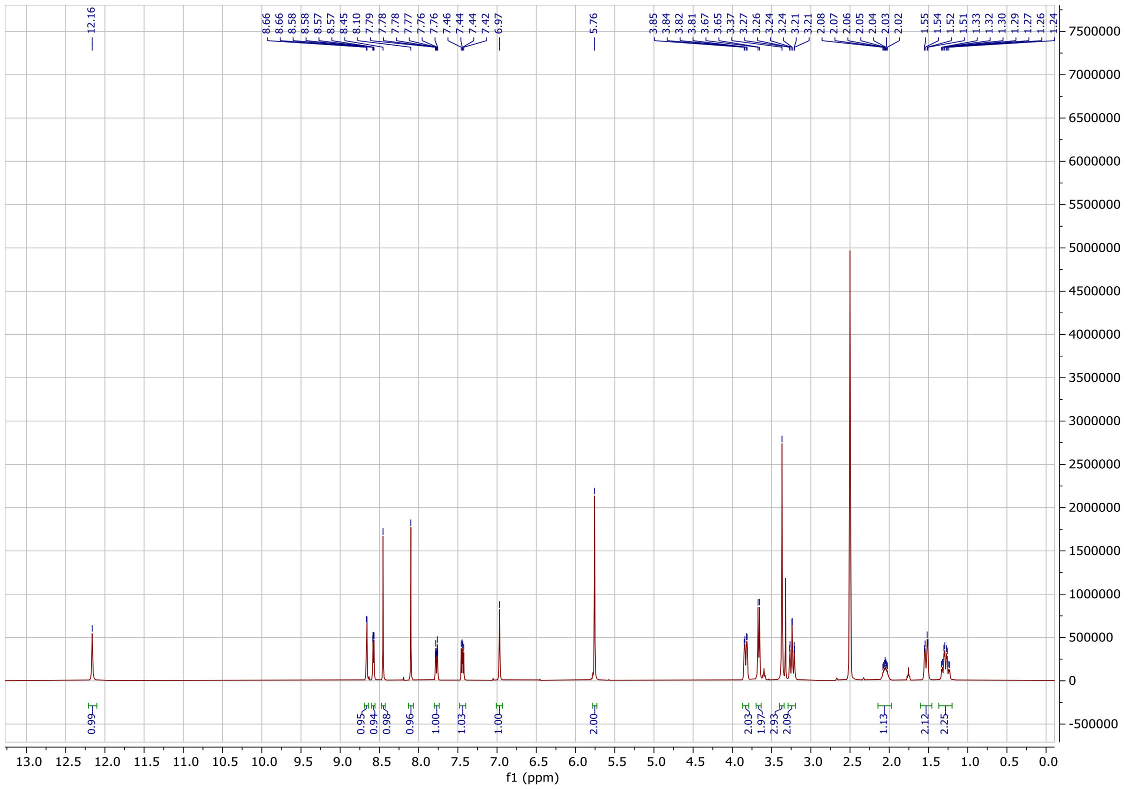

Figure S58.^{1}H NMR (400 MHz, DMSO-d_6) spectrum of compound **27b**.

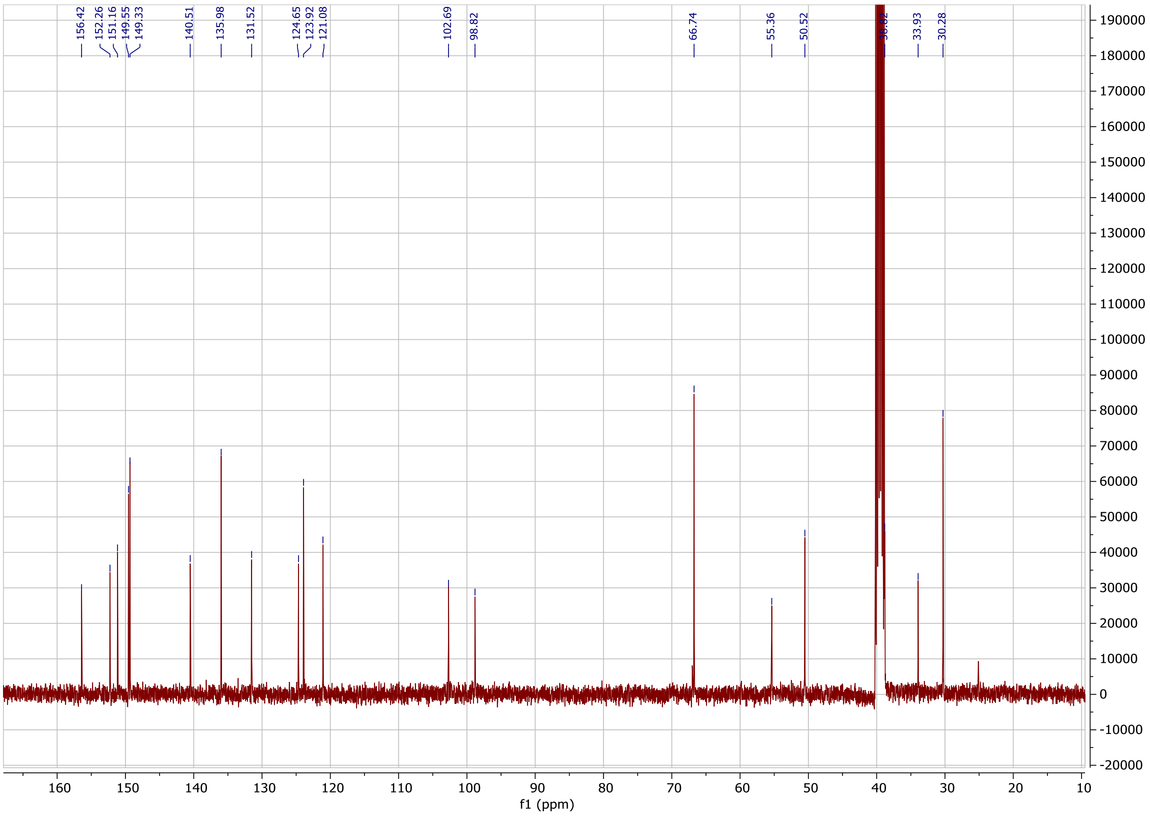

Figure S59. ^{13}C NMR (101 MHz, DMSO-d_6) spectrum of compound **27b**.

Elemental Composition Report **Page 1**

Single Mass Analysis
Tolerance = 2.0 PPM / DBE: min = -1.5, max = 50.0
Element prediction: Off
Number of isotope peaks used for i-FIT = 3

Monoisotopic Mass, Even Electron Ions
771 formula(e) evaluated with 1 results within limits (all results (up to 1000) for each mass)
Elements Used:
C: 1-100 H: 1-150 N: 0-8 O: 0-12 I: 0-1
ReqID3709 130 (1.222) AM2 (Ar,35000.0,0.00,0.00); Cm (130:131)
1: TOF MS ES+

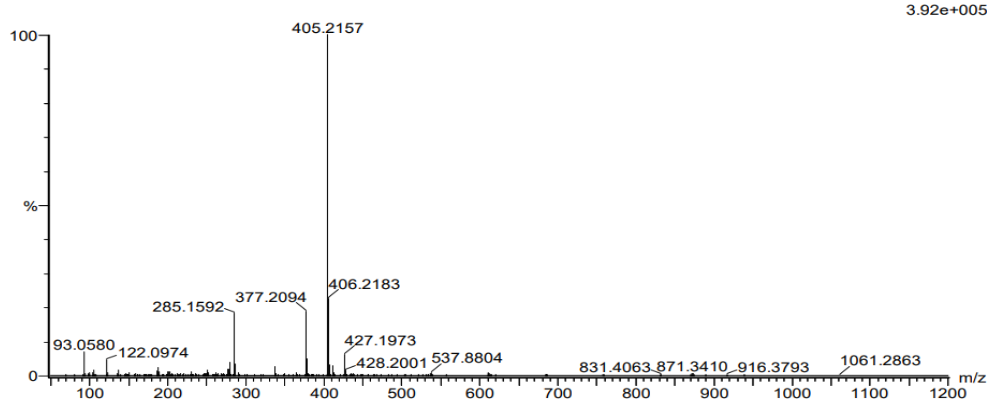

Minimum:				-1.5				
Maximum:		3000.0	2.0	50.0				
Mass	Calc. Mass	mDa	PPM	DBE	i-FIT	Norm	Conf(%)	Formula
405.2157	405.2151	0.6	1.5	13.5	749.7	n/a	n/a	C21 H25 N8 O

Figure S60. HRMS (ES+, m/z) data of compound **27b**.

Compound 28a

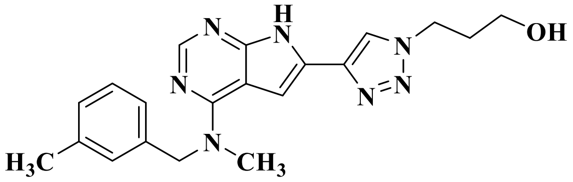

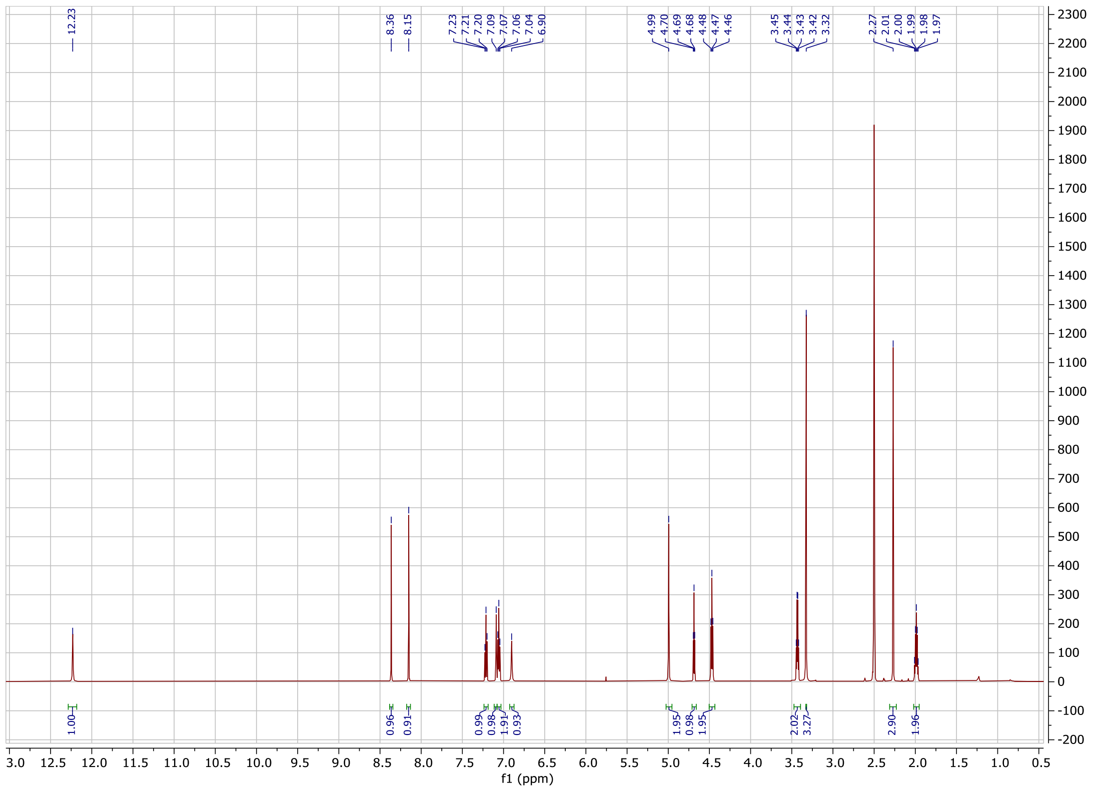

Figure S61. ^{1}H NMR (600 MHz, DMSO-d_6) spectrum of compound **28a**.

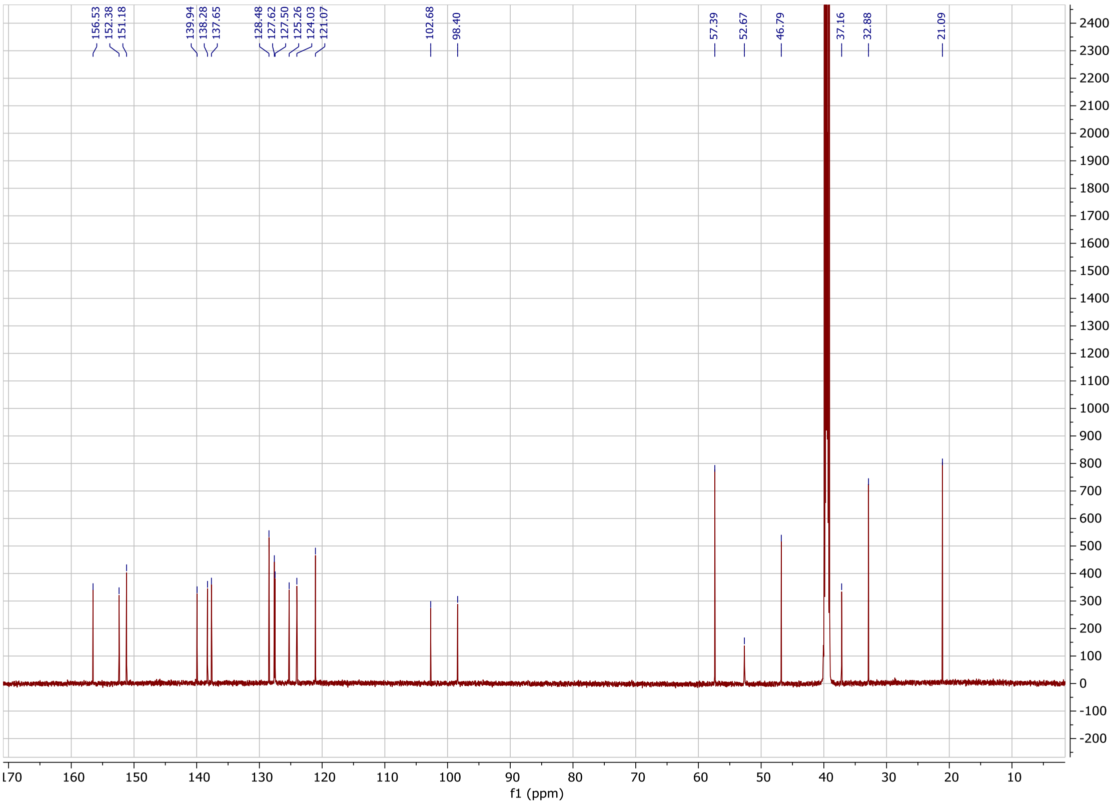

Figure S62. ^{13}C NMR (151 MHz, DMSO-d_6) spectrum of compound **28a**.

Elemental Composition Report **Page 1**

Single Mass Analysis
Tolerance = 2.0 PPM / DBE: min = -1.5, max = 50.0
Element prediction: Off
Number of isotope peaks used for i-FIT = 3

Monoisotopic Mass, Even Electron Ions
2310 formula(e) evaluated with 3 results within limits (all results (up to 1000) for each mass)
Elements Used:
C: 1-100 H: 1-150 N: 0-8 O: 0-12 F: 0-3 I: 0-1

ReqID3731 62 (0.594) AM2 (Ar,35000.0,0.00,0.00); Cm (59:62)
1: TOF MS ES+

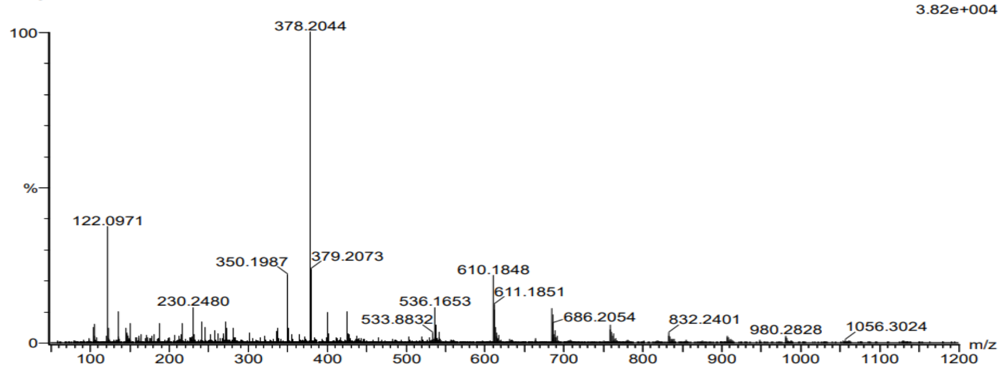

Minimum:				-1.5				
Maximum:		3000.0	2.0	50.0				
Mass	Calc. Mass	mDa	PPM	DBE	i-FIT	Norm	Conf(%)	Formula
378.2044	378.2042	0.2	0.5	12.5	489.0	0.134	87.44	C20 H24 N7 O
	378.2045	-0.1	-0.3	8.5	491.3	2.409	8.99	C22 H27 N O F3
	378.2040	0.4	1.1	3.5	492.2	3.333	3.57	C16 H29 N3 O6 F

Figure S63. HRMS (ES+, m/z) data of compound **28a**.

Compound 28b

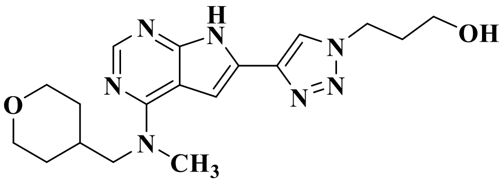

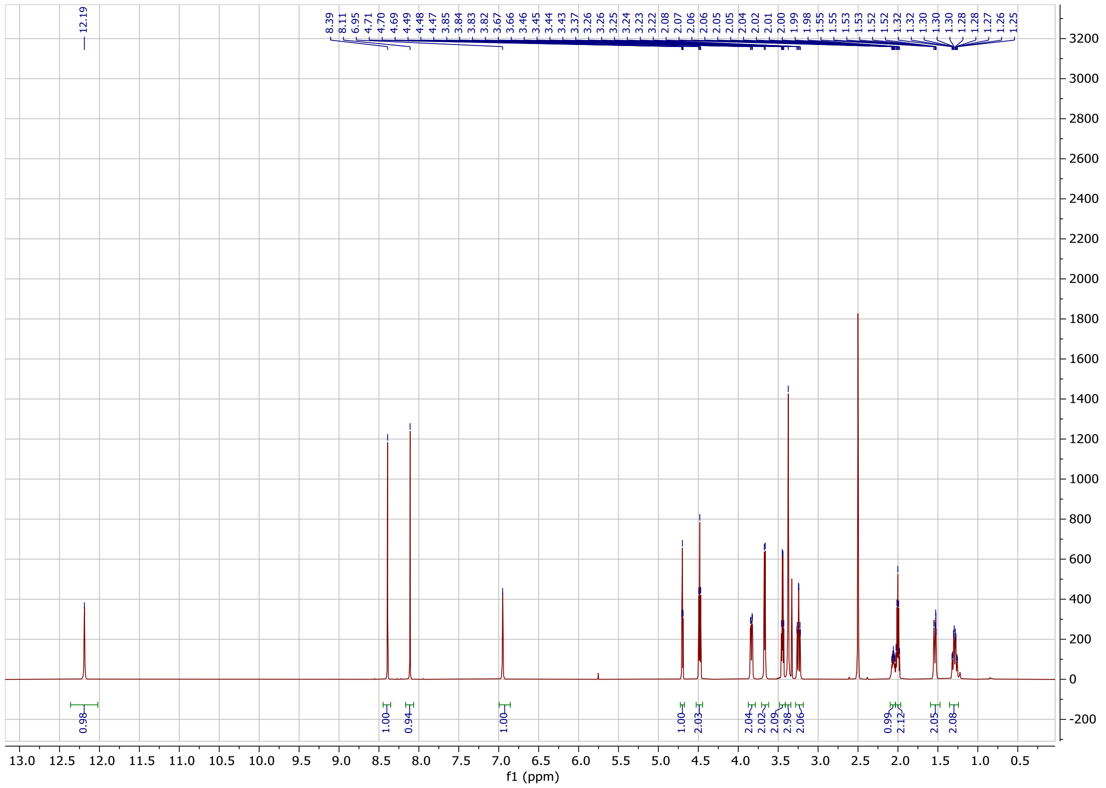

Figure S64. ^{1}H NMR (600 MHz, DMSO-d_6) spectrum of compound **28b**.

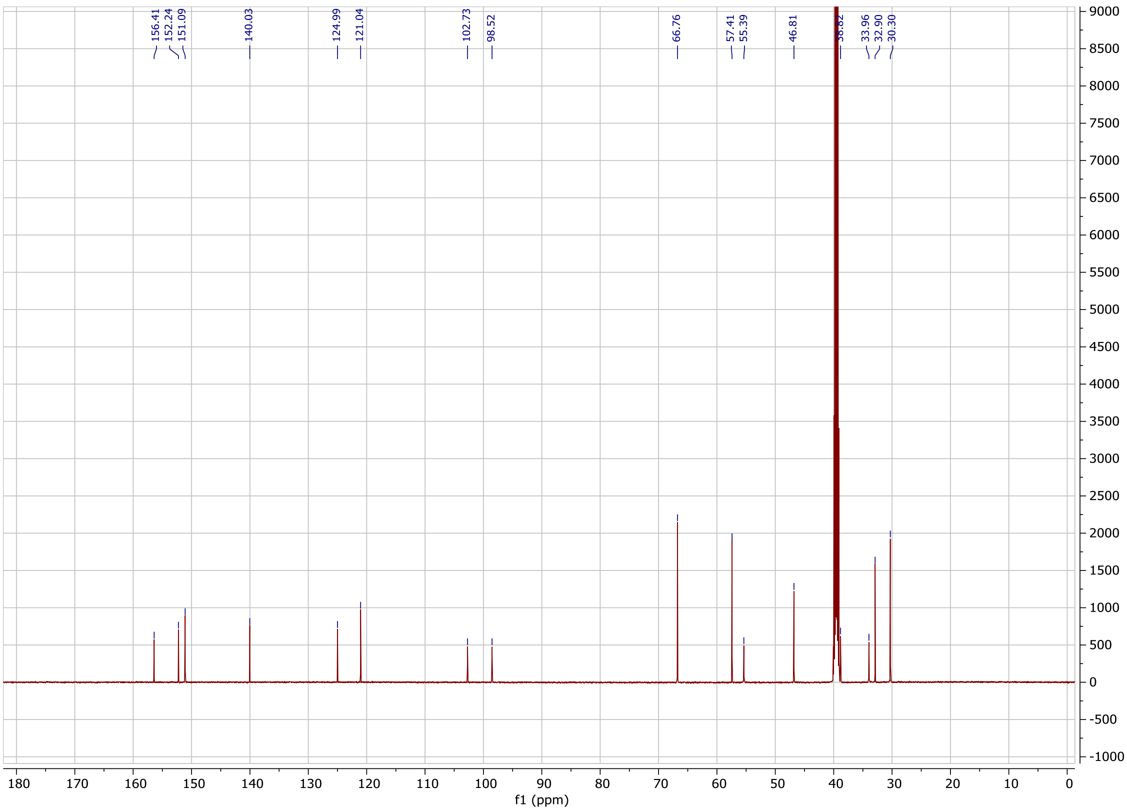

Figure S65. ^{13}C NMR (151 MHz, DMSO-d_6) spectrum of compound **28b**.

Elemental Composition Report **Page 1**

Single Mass Analysis
Tolerance = 2.0 PPM / DBE: min = -1.5, max = 50.0
Element prediction: Off
Number of isotope peaks used for i-FIT = 3

Monoisotopic Mass, Even Electron Ions
662 formula(e) evaluated with 1 results within limits (all results (up to 1000) for each mass)
Elements Used:
C: 1-100 H: 1-150 N: 0-8 O: 0-12 I: 0-1
ReqID3727 65 (0.621) AM2 (Ar,35000.0,0.00,0.00); Cm (64:65)
1: TOF MS ES+

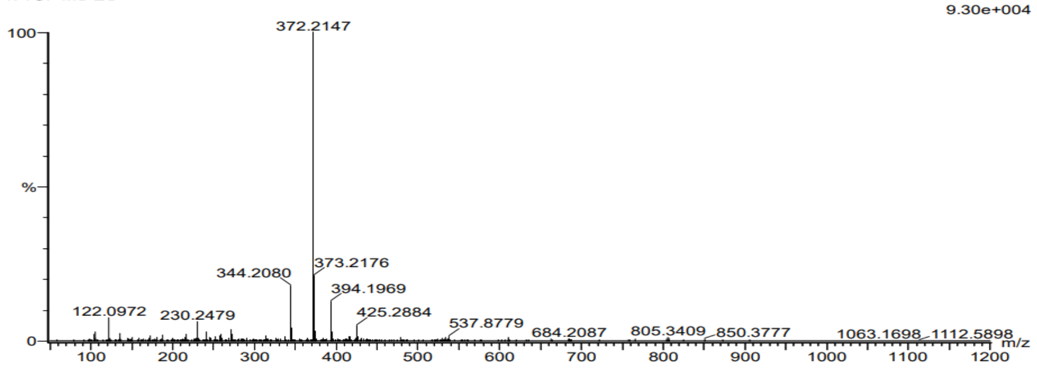

Minimum:				-1.5				
Maximum:		3000.0	2.0	50.0				
Mass	Calc. Mass	mDa	PPM	DBE	i-FIT	Norm	Conf(%)	Formula
372.2147	372.2148	-0.1	-0.3	9.5	488.8	n/a	n/a	C18 H26 N7 O2

Figure S66. HRMS (ES+, m/z) data of compound **28b**.

Compound 29a

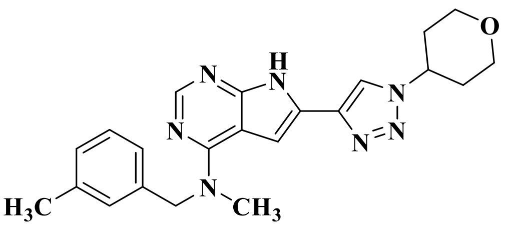

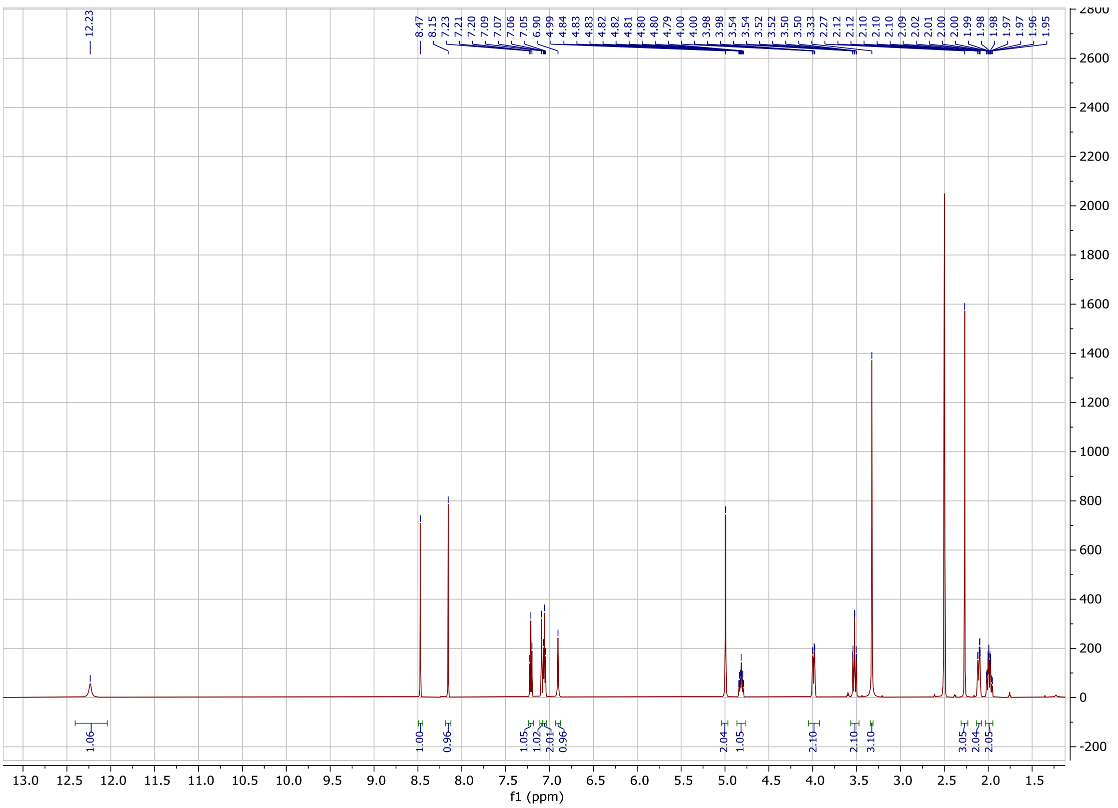

Figure S67. ^{1}H NMR (600 MHz, DMSO-d_6) spectrum of compound **29a**.

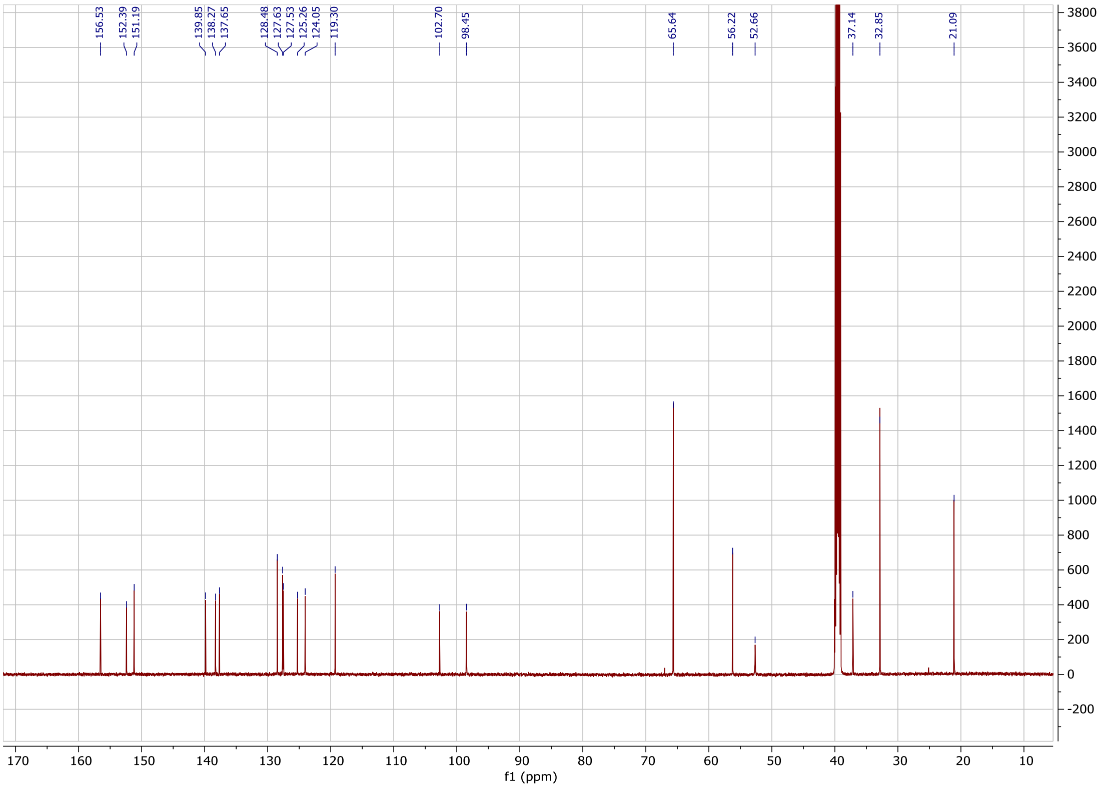

Figure S68. ^{13}C NMR (151 MHz, DMSO-d_6) spectrum of compound **29a**.

Elemental Composition Report **Page 1**

Single Mass Analysis
Tolerance = 2.0 PPM / DBE: min = -1.5, max = 50.0
Element prediction: Off
Number of isotope peaks used for i-FIT = 3

Monoisotopic Mass, Even Electron Ions
764 formula(e) evaluated with 1 results within limits (all results (up to 1000) for each mass)
Elements Used:
C: 1-100 H: 1-150 N: 0-8 O: 0-12 I: 0-1
ReqID3700 57 (0.543) AM2 (Ar,35000.0,0.00,0.00); Cm (55:57)
1: TOF MS ES+

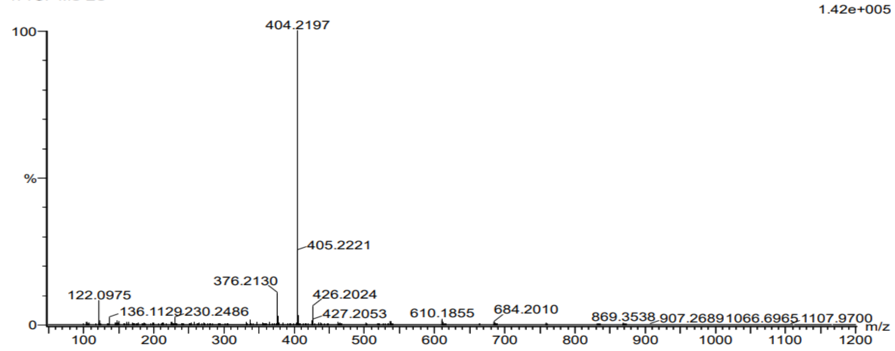

Minimum:				-1.5				
Maximum:		3000.0	2.0	50.0				
Mass	Calc. Mass	mDa	PPM	DBE	i-FIT	Norm	Conf(%)	Formula
404.2197	404.2199	-0.2	-0.5	13.5	544.5	n/a	n/a	C22 H26 N7 O

Figure S69. HRMS (ES+, m/z) data of compound **29a**.

Compound 29b

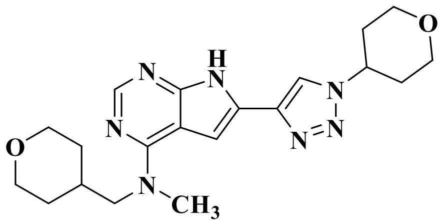

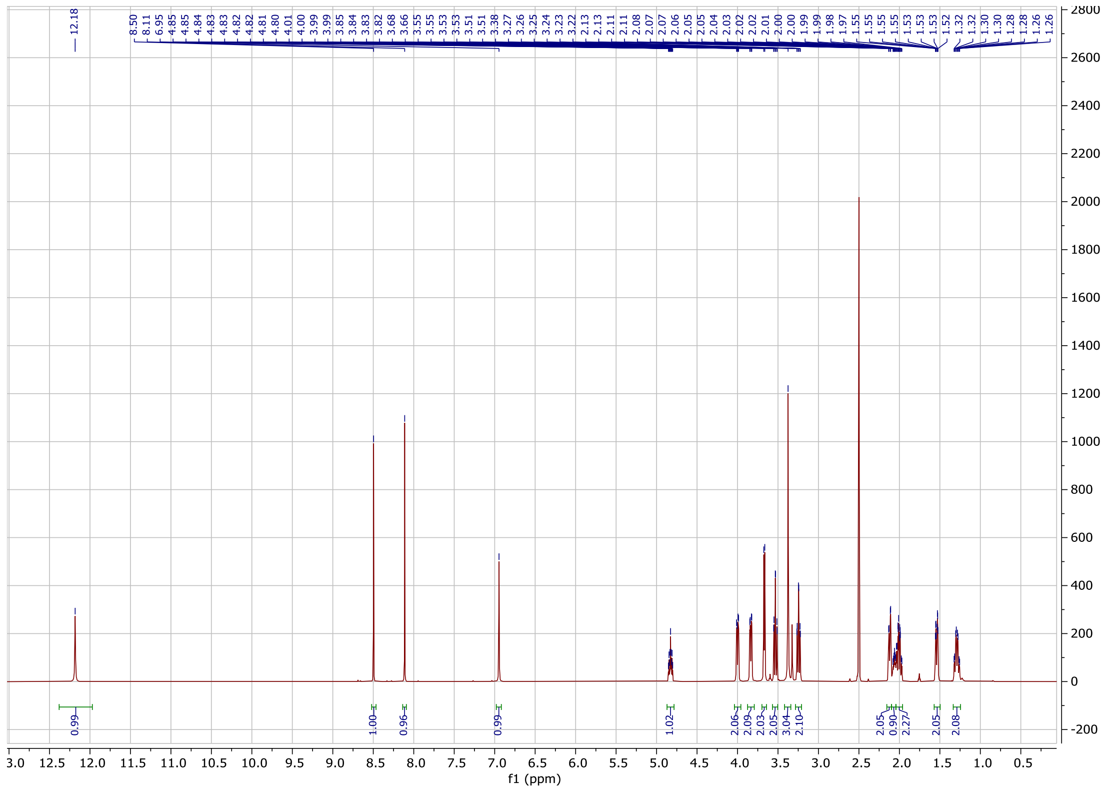

Figure S70. ^{1}H NMR (600 MHz, DMSO-d_6) spectrum of compound **29b**.

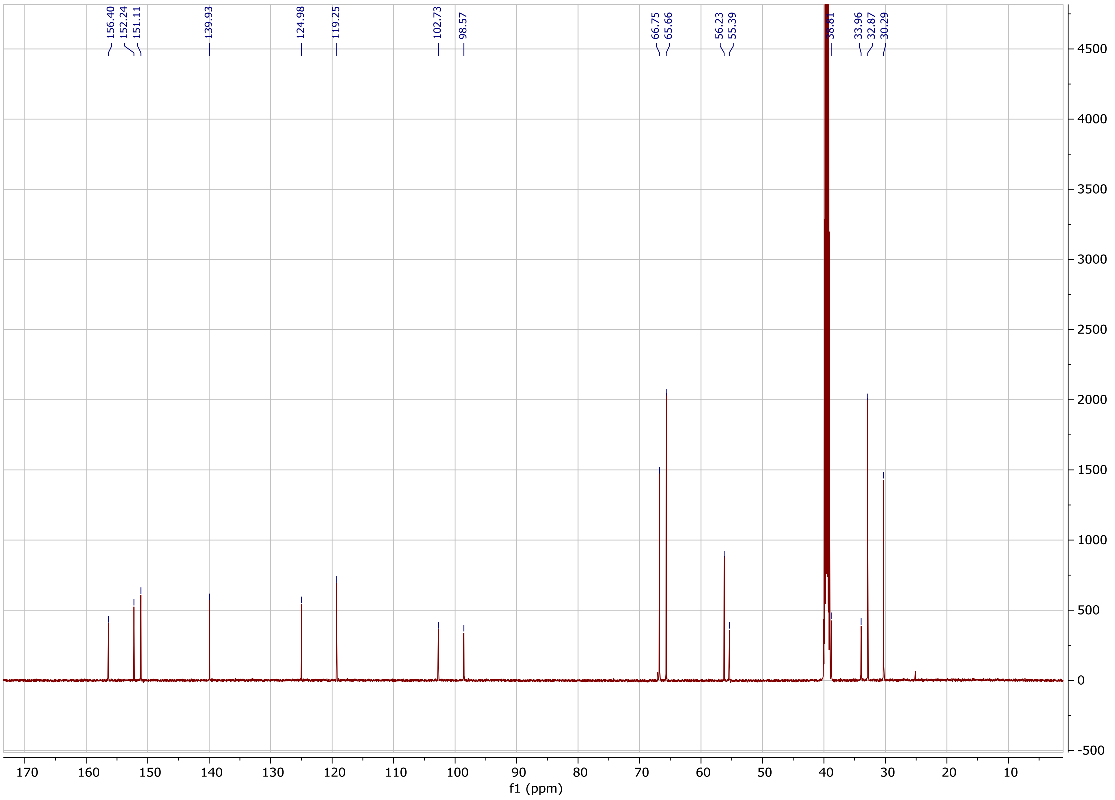

Figure S71. ^{13}C NMR (151 MHz, DMSO-d_6) spectrum of compound **29b**.

Elemental Composition Report **Page 1**

Single Mass Analysis
Tolerance = 2.0 PPM / DBE: min = -1.5, max = 50.0
Element prediction: Off
Number of isotope peaks used for i-FIT = 3

Monoisotopic Mass, Even Electron Ions
748 formula(e) evaluated with 1 results within limits (all results (up to 1000) for each mass)
Elements Used:
C: 1-100 H: 1-150 N: 0-8 O: 0-12 I: 0-1
ReqID3719 60 (0.577) AM2 (Ar,35000.0,0.00,0.00); Cm (59:60)
1: TOF MS ES+

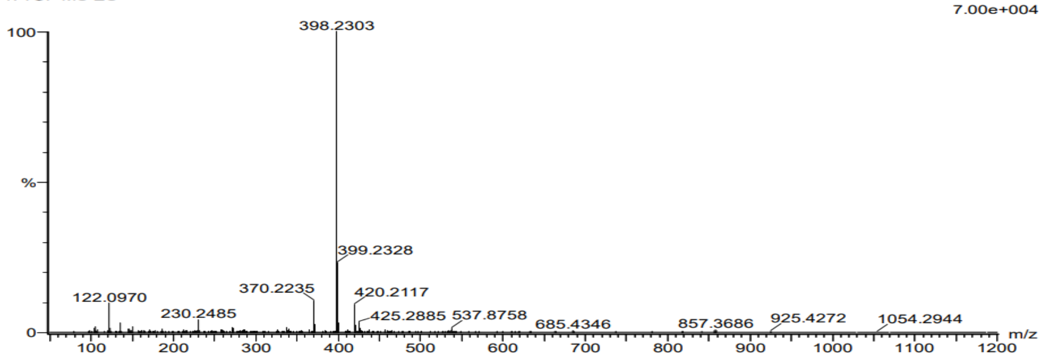

Minimum:				-1.5				
Maximum:		3000.0	2.0	50.0				
Mass	Calc. Mass	mDa	PPM	DBE	i-FIT	Norm	Conf(%)	Formula
398.2303	398.2304	-0.1	-0.3	10.5	486.0	n/a	n/a	C20 H28 N7 O2

Figure S72. HRMS (ES+, m/z) data of compound **29b**.

Compound 30a

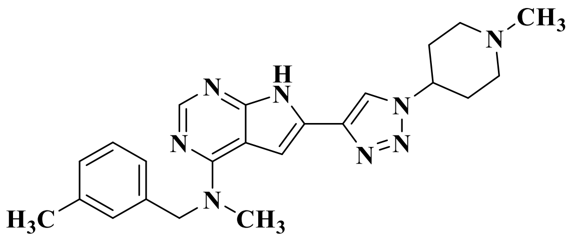

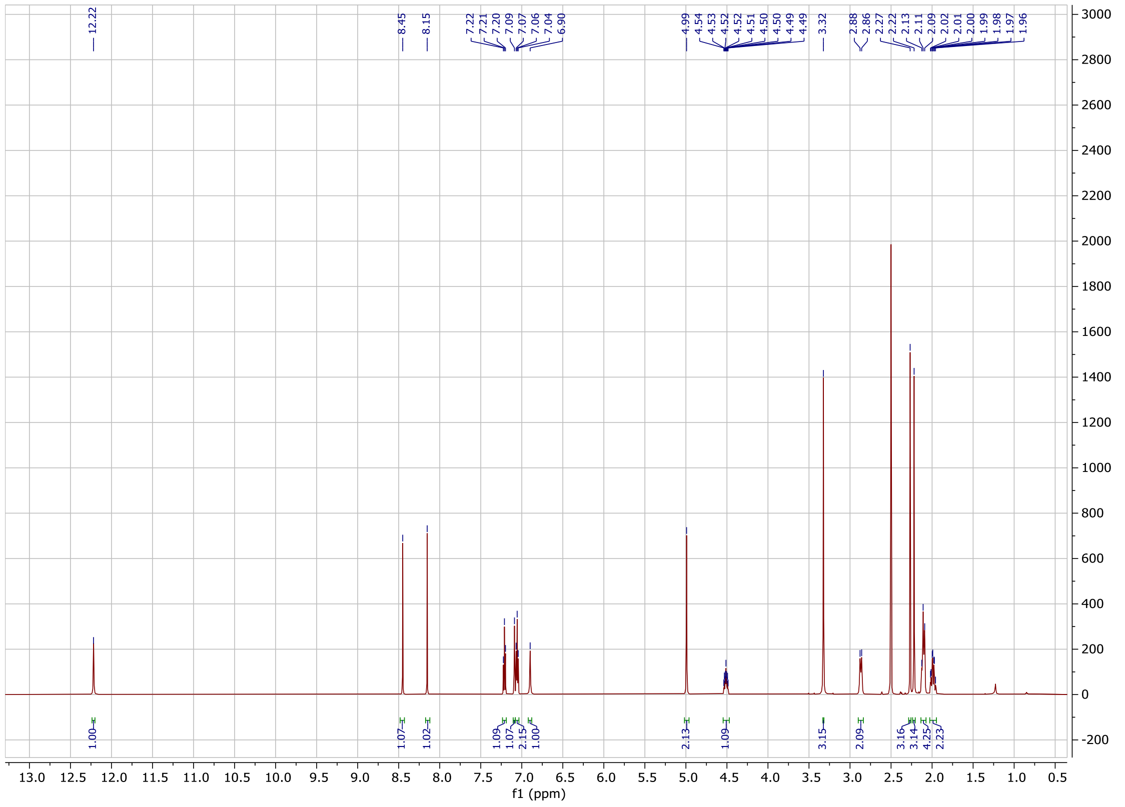

Figure S73. ^{1}H NMR (600 MHz, DMSO-d_6) spectrum of compound **30a**.

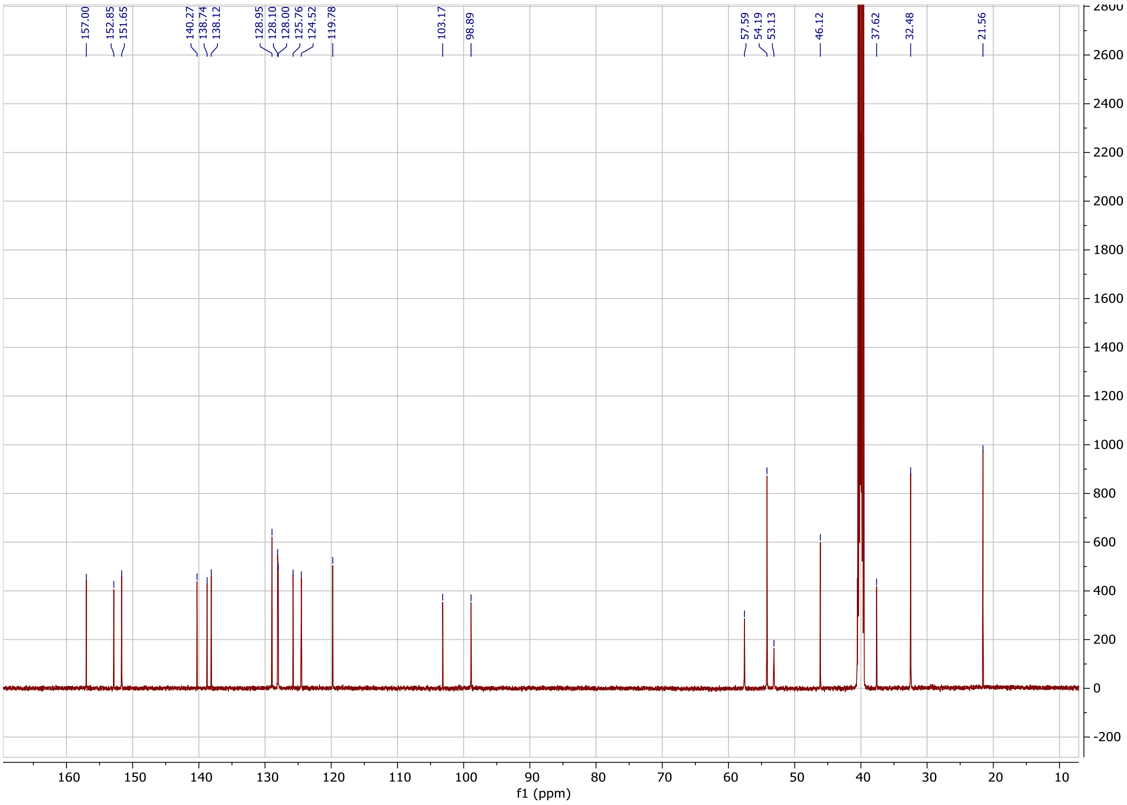

Figure S74. ^{13}C NMR (151 MHz, DMSO-d_6) spectrum of compound **30a**.

Elemental Composition Report **Page 1**

Single Mass Analysis
Tolerance = 2.0 PPM / DBE: min = -1.5, max = 50.0
Element prediction: Off
Number of isotope peaks used for i-FIT = 3

Monoisotopic Mass, Even Electron Ions
802 formula(e) evaluated with 1 results within limits (all results (up to 1000) for each mass)
Elements Used:
C: 1-100 H: 1-150 N: 0-8 O: 0-12 I: 0-1
ReqID3722 190 (1.787) AM2 (Ar,35000.0,0.00,0.00); Cm (190:192)
1: TOF MS ES+

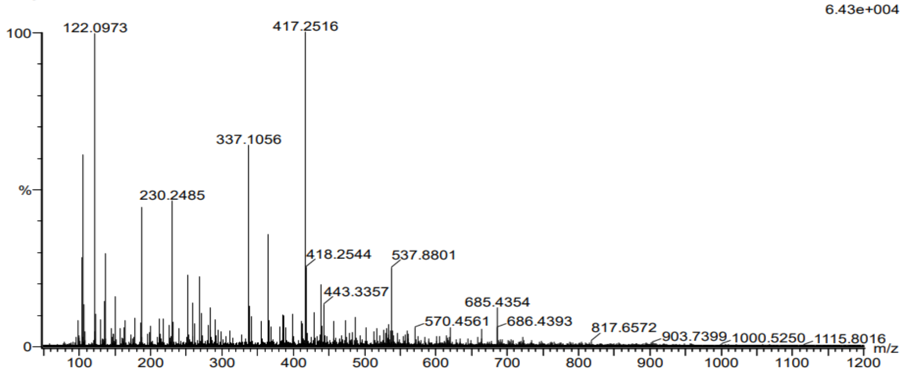

Minimum:				-1.5				
Maximum:		3000.0	2.0	50.0				
Mass	Calc. Mass	mDa	PPM	DBE	i-FIT	Norm	Conf(%)	Formula
417.2516	417.2515	0.1	0.2	13.5	646.2	n/a	n/a	C23 H29 N8

Figure S75. HRMS (ES+, m/z) data of compound **30a**.

Compound 30b

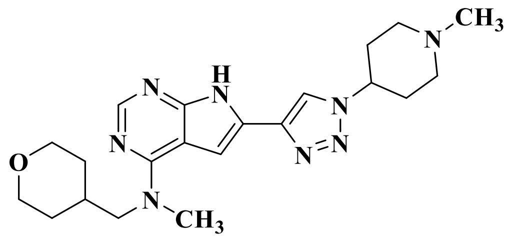

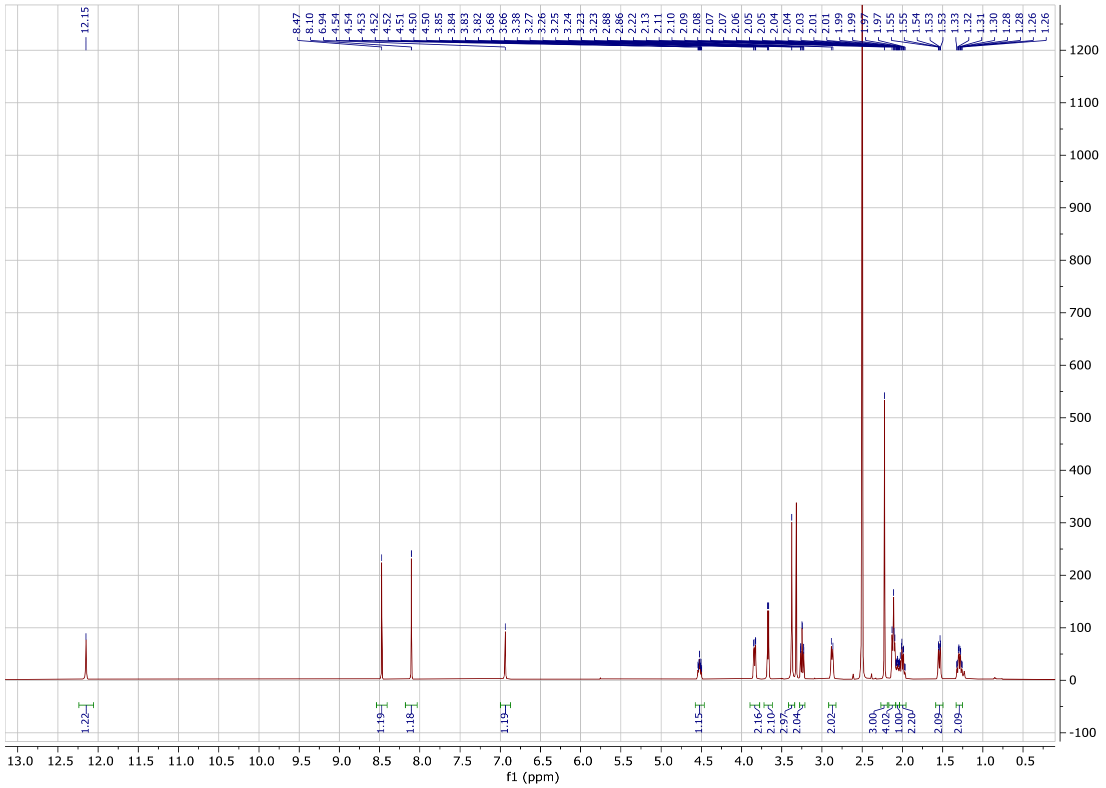

Figure S76. ^{1}H NMR (600 MHz, DMSO-d_6) spectrum of compound **30b**.

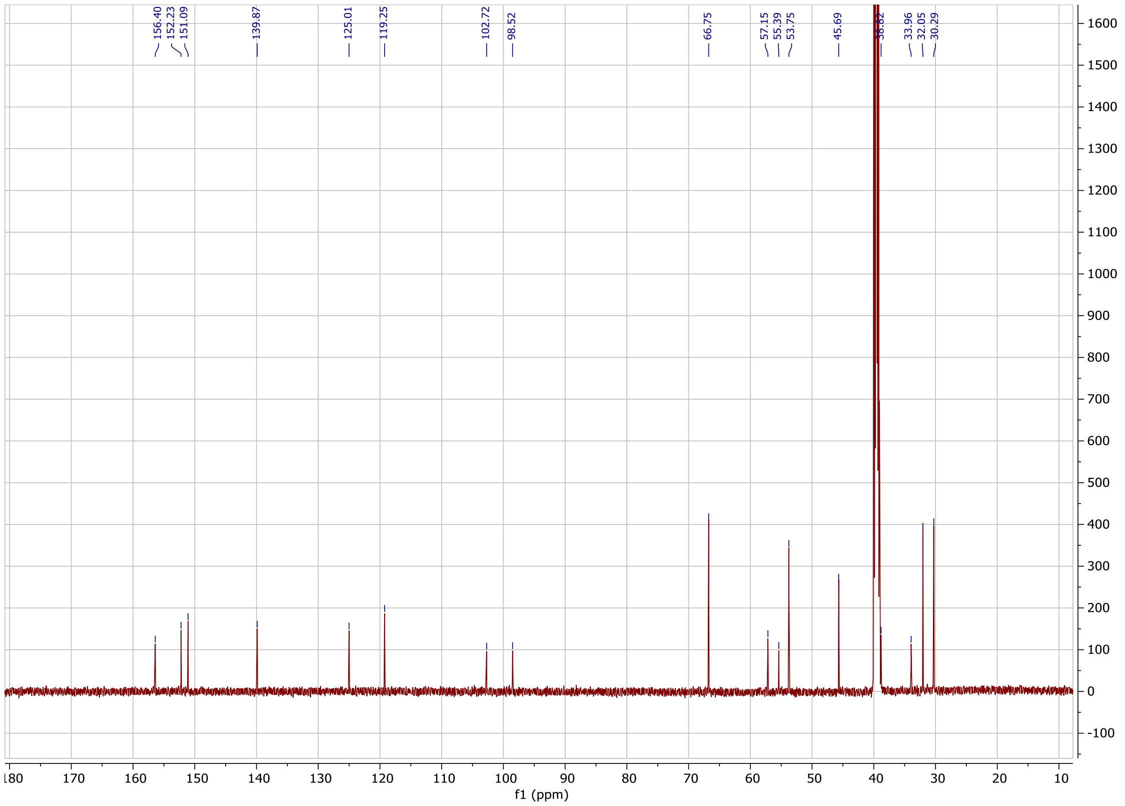

Figure S77. ^{13}C NMR (151 MHz, DMSO-d_6) spectrum of compound **30b**.

Elemental Composition Report **Page 1**

Single Mass Analysis
Tolerance = 2.0 PPM / DBE: min = -1.5, max = 50.0
Element prediction: Off
Number of isotope peaks used for i-FIT = 3

Monoisotopic Mass, Even Electron Ions
788 formula(e) evaluated with 1 results within limits (all results (up to 1000) for each mass)
Elements Used:
C: 1-100 H: 1-150 N: 0-8 O: 0-12 I: 0-1
ReqID3708 190 (1.787) AM2 (Ar,35000.0,0.00,0.00); Cm (190:191)
1: TOF MS ES+

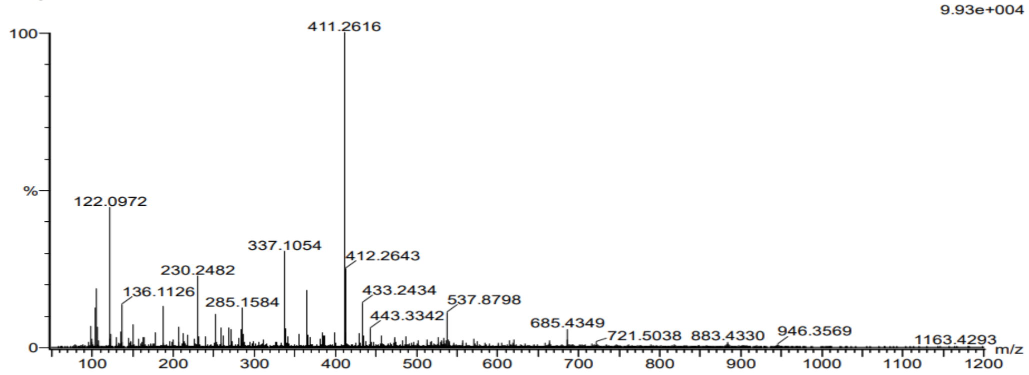

Minimum:				-1.5				
Maximum:		3000.0	2.0	50.0				
Mass	Calc. Mass	mDa	PPM	DBE	i-FIT	Norm	Conf(%)	Formula
411.2616	411.2621	-0.5	-1.2	10.5	707.6	n/a	n/a	C21 H31 N8 O

Figure S78. HRMS (ES+, m/z) data of compound **30b**.

Compound 31a

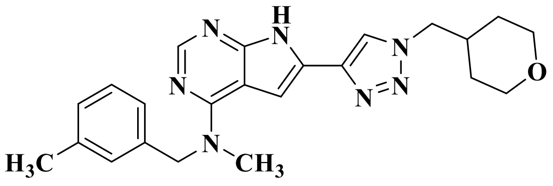

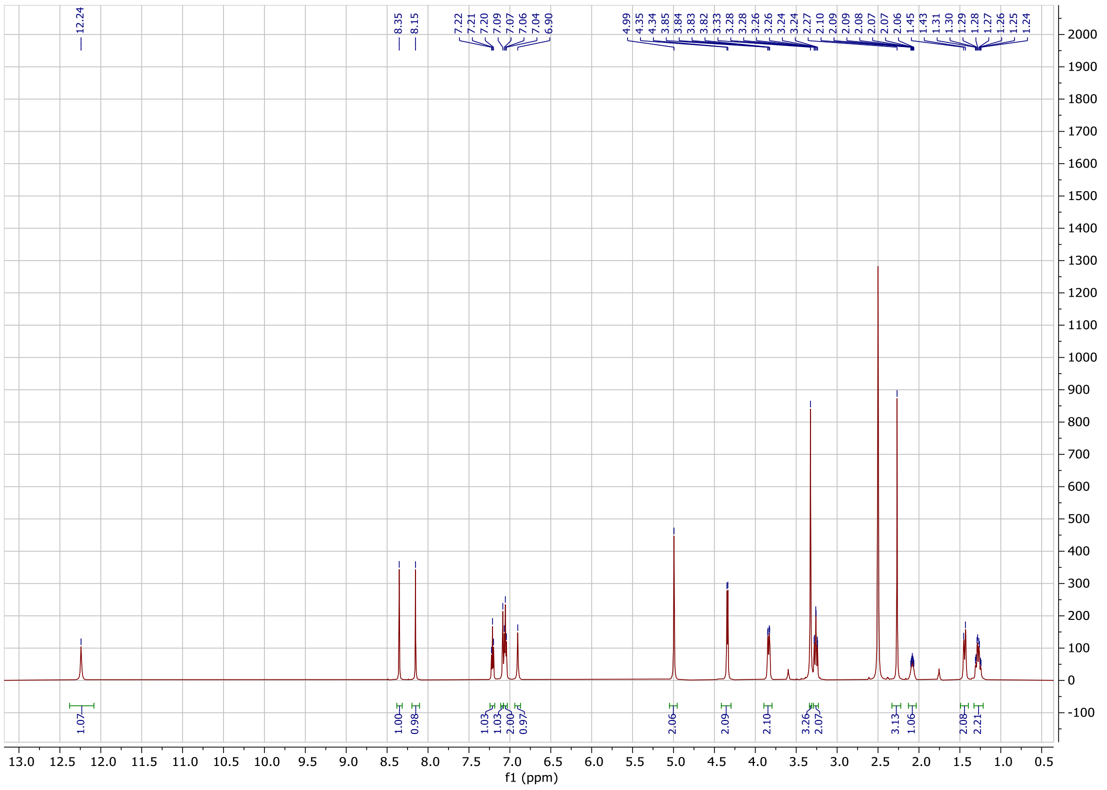

Figure S79. ^{1}H NMR (600 MHz, DMSO-d_6) spectrum of compound **31a**.

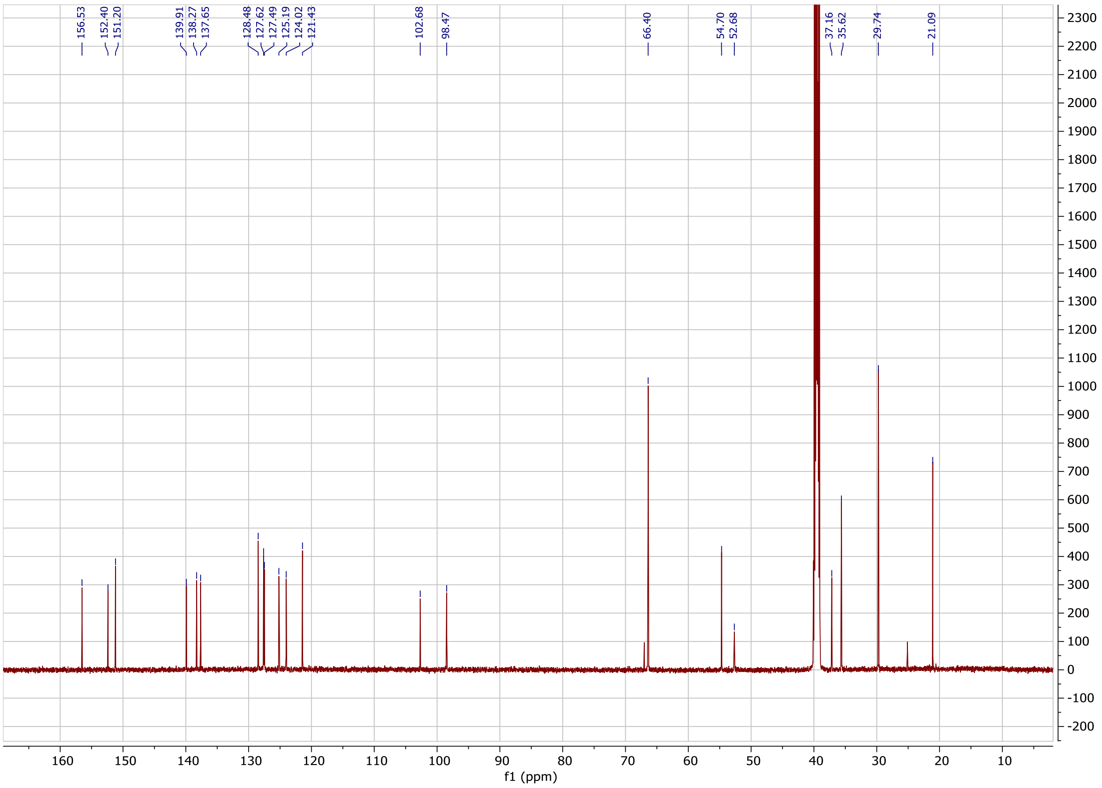

Figure S80. ^{13}C NMR (151 MHz, DMSO-d_6) spectrum of compound **31a**

Elemental Composition Report **Page 1**

Single Mass Analysis
Tolerance = 2.0 PPM / DBE: min = -1.5, max = 50.0
Element prediction: Off
Number of isotope peaks used for i-FIT = 3

Monoisotopic Mass, Even Electron Ions
805 formula(e) evaluated with 1 results within limits (all results (up to 1000) for each mass)
Elements Used:
C: 1-100 H: 1-150 N: 0-8 O: 0-12 I: 0-1

ReqID3733 57 (0.543) AM2 (Ar,35000.0,0.00,0.00); Cm (55:57)
1: TOF MS ES+

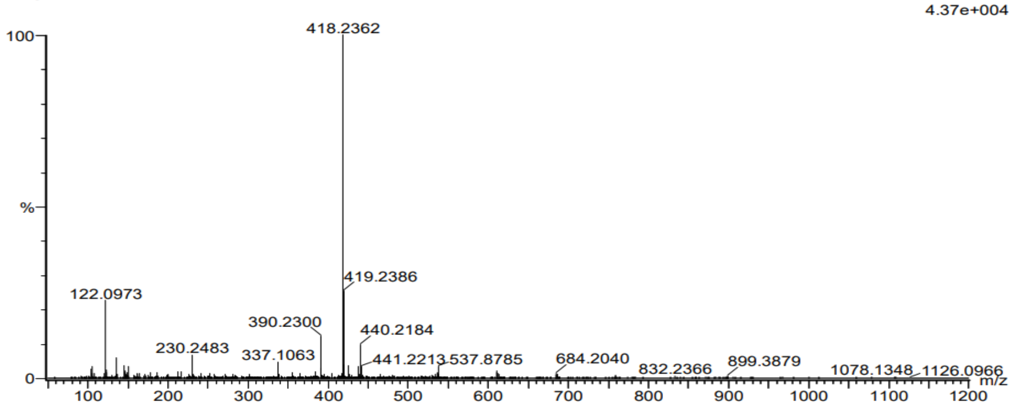

Minimum:				-1.5				
Maximum:		3000.0	2.0	50.0				
Mass	Calc. Mass	mDa	PPM	DBE	i-FIT	Norm	Conf(%)	Formula
418.2362	418.2355	0.7	1.7	13.5	383.6	n/a	n/a	C23 H28 N7 O

Figure S81. HRMS (ES+, m/z) data of compound **31a**.

Compound 31b

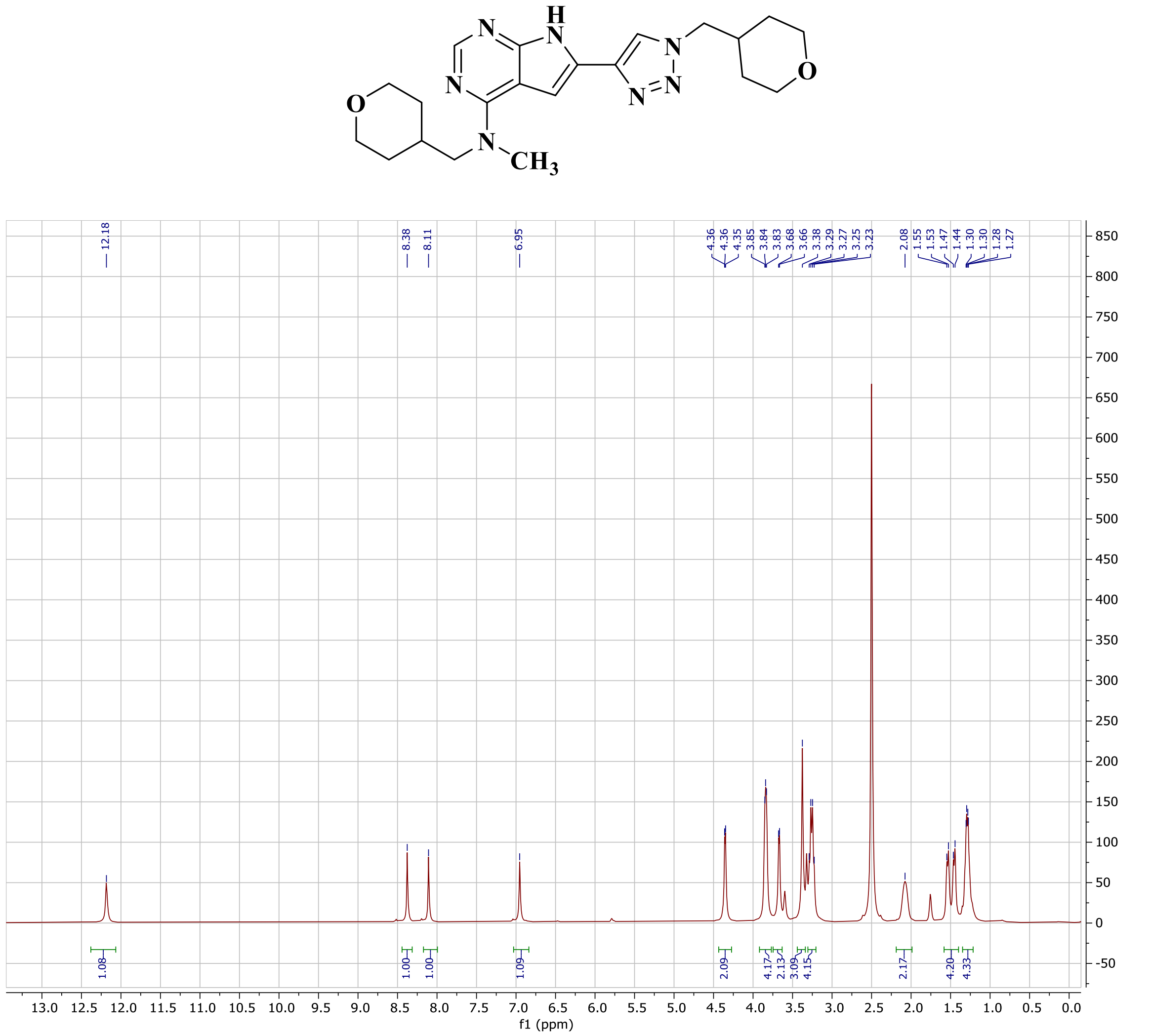

Figure S82. ^{1}H NMR (600 MHz, DMSO-d_6) spectrum of compound **31b**.

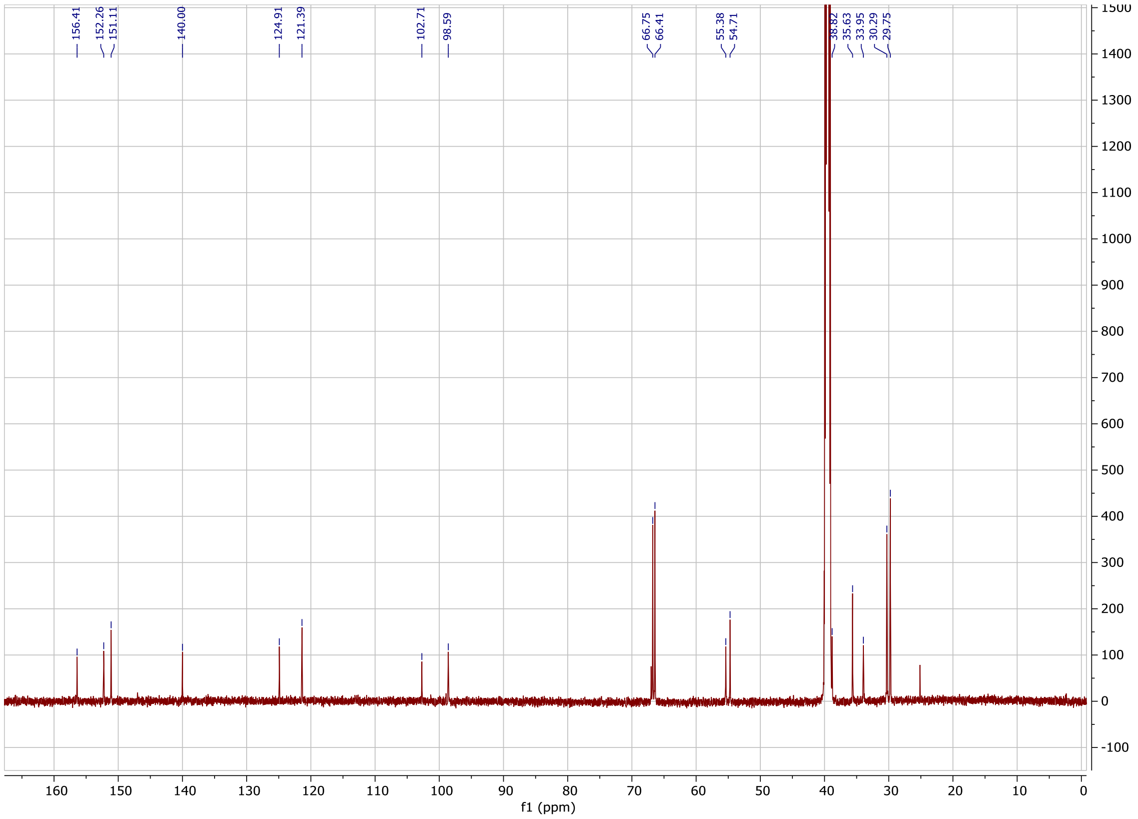

Figure S83. ^{13}C NMR (151 MHz, DMSO-d_6) spectrum of compound **31b**.

Elemental Composition Report **Page 1**

Single Mass Analysis
Tolerance = 2.0 PPM / DBE: min = -1.5, max = 50.0
Element prediction: Off
Number of isotope peaks used for i-FIT = 3

Monoisotopic Mass, Even Electron Ions
791 formula(e) evaluated with 1 results within limits (all results (up to 1000) for each mass)
Elements Used:
C: 1-100 H: 1-150 N: 0-8 O: 0-12 I: 0-1
ReqID3702 68 (0.647) AM2 (Ar,35000.0,0.00,0.00); Cm (65:68)
1: TOF MS ES+

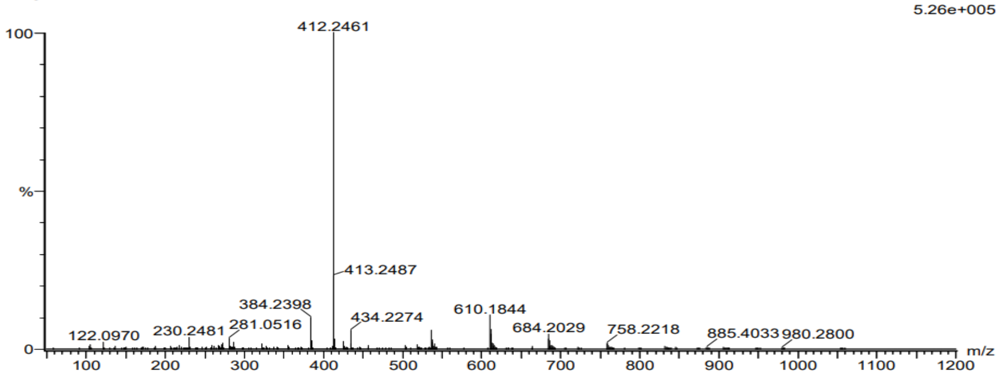

Minimum:				-1.5				
Maximum:		3000.0	2.0	50.0				
Mass	Calc. Mass	mDa	PPM	DBE	i-FIT	Norm	Conf(%)	Formula
412.2461	412.2461	0.0	0.0	10.5	801.7	n/a	n/a	C21 H30 N7 O2

Figure S84. HRMS (ES+, m/z) data of compound **31b.**

5. Molecular docking

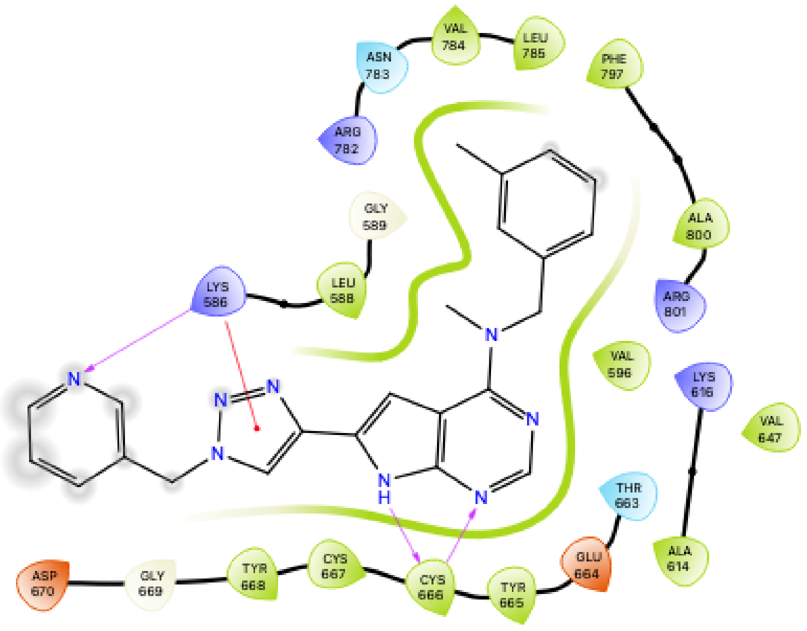

Figure S85. Ligand interaction diagram for docking of **27a** into CSF1R contruct 8CGC.pdb.

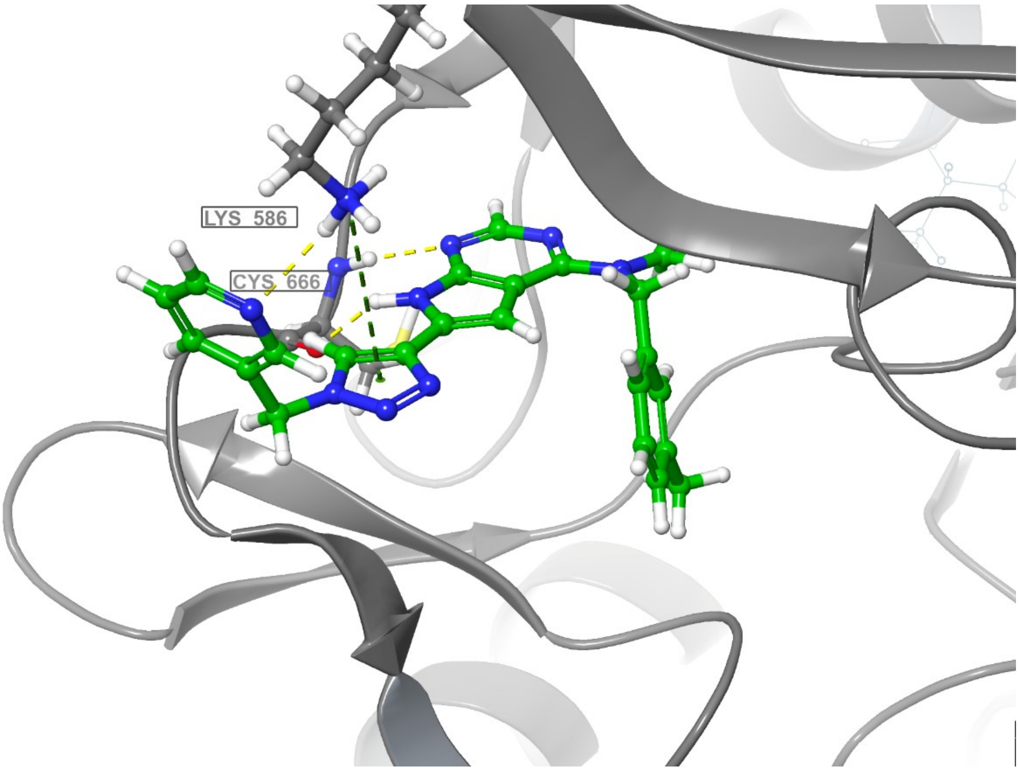

Figure S86. Docking of 27a into CSF1R contruct 8CGC.pdb

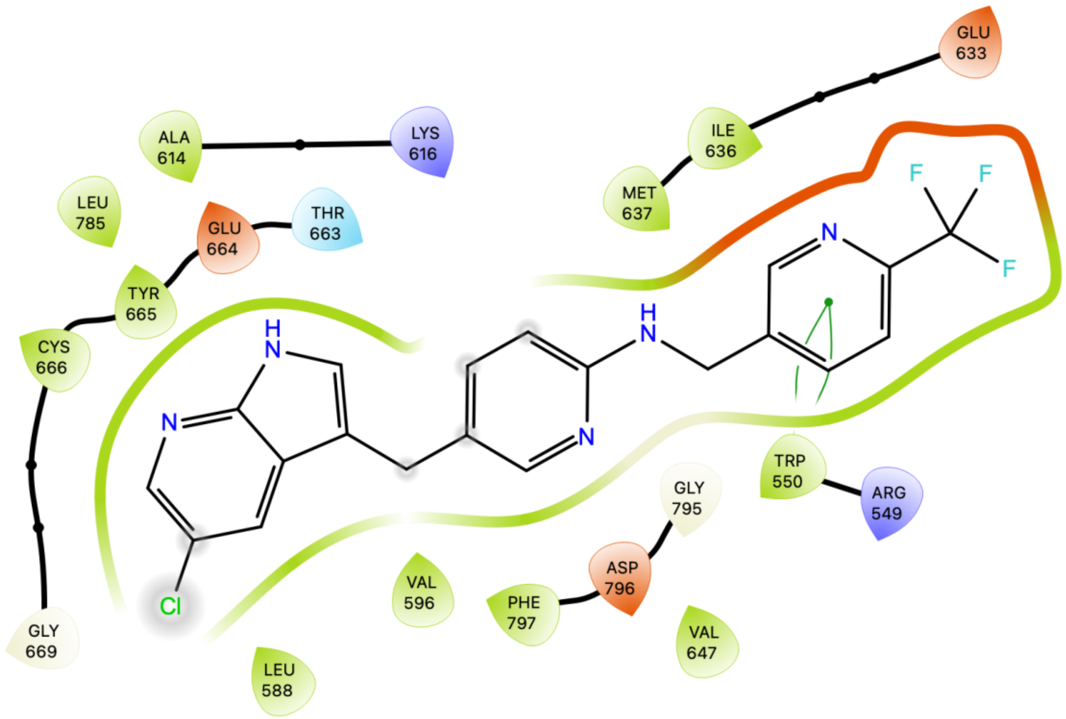

Figure S87. Ligand interaction diagram for PLX-3397 co-crystal with CSF1R contruct 4R7H.pdb.

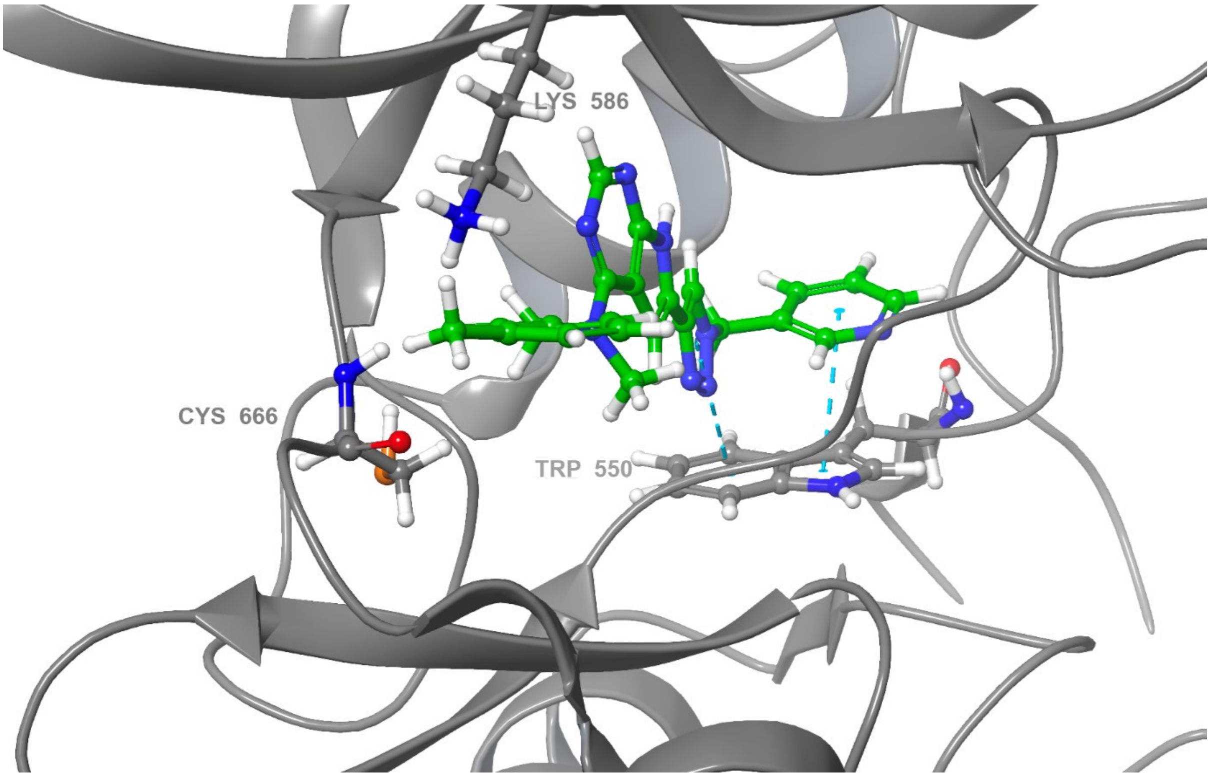

Figure S88. Docking of **27a** into CSF1R contruct 4R7H.pdb.

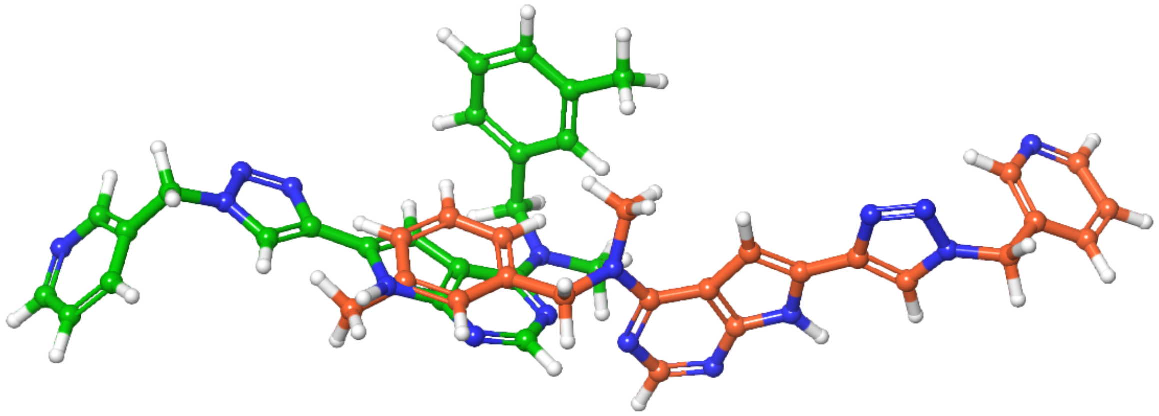

Figure S89. Overlay of **the docked structure of 27a** into CSF1R contruct 4R7H.pdb (orange structure) and 8CGC.pdb (green structure). Nitrogen atoms are coloured blue.

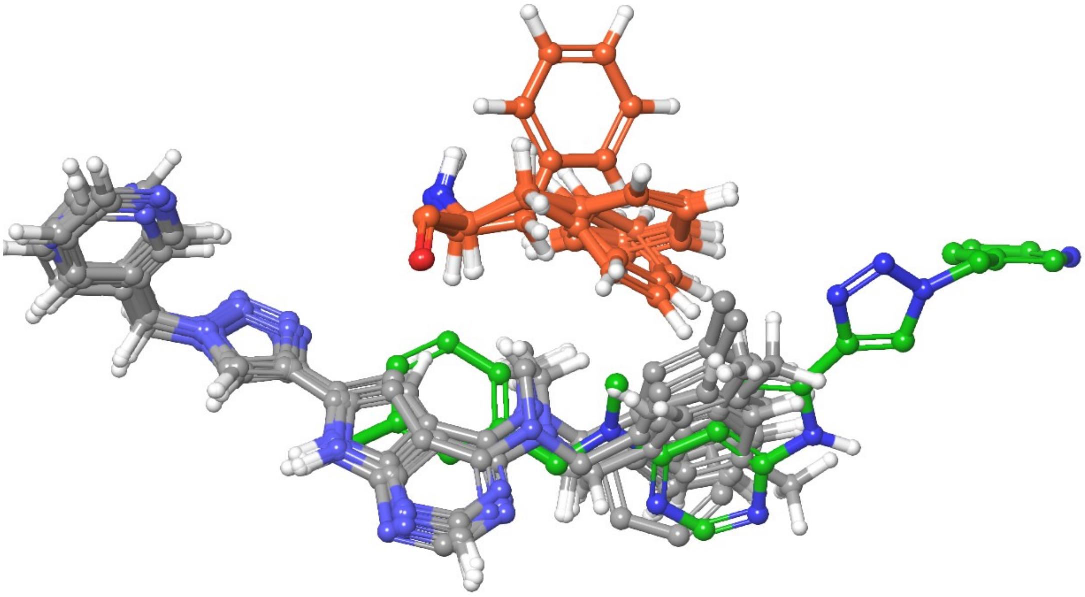

Figure S90. Induced-fit docking of of 27a into CSF1R contruct 4R7H. Seven low energy poses (grey) and one high energy pose with “inverted” binding pose (green) were found. Variation in the position of Phe-797 is shown in orange. The largest flip in Phe-797 is not connected to the “inverted” binding mode.
